# Novel CDK2/4/6 inhibitor culmerciclib (TQB3616) plus fulvestrant in previously treated, HR-positive, HER2-negative advanced breast cancer: a randomized, double-blind, phase 3 trial

**DOI:** 10.1038/s41392-025-02475-6

**Published:** 2025-12-18

**Authors:** Yongmei Yin, Qingyuan Zhang, Tao Sun, Chunfang Hao, Zhihong Wang, Jin Yang, Yongsheng Wang, Yanxia Shi, Jing Sun, Quchang Ouyang, Haichuan Su, Jinsheng Wu, Lu Gan, Meng Han, Liming Gao, Xiaojia Wang, Bing Zhao, Hui Li, Jiuda Zhao, Hongwei Yang, Fangling Ning, Fuguo Tian, Juliang Zhang, Hongmei Sun, Zhaofeng Niu, Hong Zong, Aimin Zang, Xinshuai Wang, Xinyu Qian, Shikai Wu, Jianyun Nie, Lijia He, Ying Cheng, Yanrong Hao, Yi Zhai, Huiping Li, Jingfen Wang, Shihong Wei, Man Li, Yunjiang Liu, Hongqiang Guo, Qun Hu, Lina Liu, Xinghua Han, Ruizhen Luo, Mingli Ni, Xianjun Tang, Zhenhua Zhai, Meiqian Ding, Haibo Wang, Peng Shen, Xian Wang, Lian Liu, Wenyan Chen, Gang Liu, Zhengwen Cai, Zefei Jiang

**Affiliations:** 1 Department of Oncology, The First Affiliated Hospital with Nanjing Medical University, Nanjing, Jiangsu China; 2 Breast Internal Medicine Ward 1, Harbin Medical University Cancer Hospital, Harbin, Heilongjiang China; 3 Department of Breast Internal Medicine, Liaoning Cancer Hospital & Institute, Shenyang, Liaoning China; 4 Department of Breast Oncology, Tianjin Medical University Cancer Institute & Hospital/Tianjin Cancer Hospital Airport Hospital, Tianjin, China; 5 Department of Breast and Thyroid Surgery, the Affiliated Cancer Hospital of Guizhou Medical University, Guiyang, Guizhou China; 6 Department of Oncology, the First Affiliated Hospital of Xi’an Jiaotong University, Xi’an, Shaanxi China; 7 Breast Surgery Ward One, Shandong Cancer Hospital, Jinan, Shandong China; 8 Department of Medical Oncology, Sun Yat-sen University Cancer Center, Guangzhou, Guangdong China; 9 Department of Oncology, Anyang Tumor Hospital, Anyang, Henan China; 10 Department of Breast Internal Medicine, Hunan Cancer Hospital, Changsha, Hunan China; 11 Department of Oncology, the Second Affiliated Hospital of Chinese PLA Air Force Medical University, Xi’an, Shaanxi China; 12 Department of Radiation Oncology, the First Affiliated Hospital of Hainan Medical University, Haikou, Hainan China; 13 Department of Medical Oncology, the First Affiliated Hospital of Chongqing Medical University, Chongqing, China; 14 Department of Breast Surgery, the First hospital of Qinhuangdao, Qinhuangdao, Hebei China; 15 Department of Oncology, the First hospital of Qinhuangdao, Qinhuangdao, Hebei China; 16 Department of Breast Surgery, Zhejiang Cancer Hospital, Hangzhou, Zhejiang China; 17 Department of Breast Internal Medicine, Cancer Hospital of Xinjiang medical University, Urumqi, Xinjiang China; 18 Department of Breast Surgery, Sichuan Cancer Hospital, Chengdu, Sichuan China; 19 Breast Disease Diagnosis and Treatment Center, Qinghai University Affiliated Hospital, Xining, Qinghai China; 20 Department of Breast and Thyroid Surgery, Suining Central Hospital, Suining, Sichuan China; 21 Department of Oncology, Binzhou Medical College Affiliated Hospital, Binzhou, Shandong China; 22 Department of Breast surgery, Shanxi Cancer Hospital, Taiyuan, Shanxi China; 23 Department of Thyroid and breast vascular surgery, The First Affiliated Hospital of Chinese PLA Air Force Medical University, Xi’an, Shaanxi China; 24 Department of Oncology, Jiamusi Cancer Hospital, Jiamusi, Heilongjiang China; 25 Department of Breast, Yuncheng Central Hospital, Yuncheng, Shanxi China; 26 Department of Oncology, the First Affiliated Hospital of Zhengzhou University, Zhengzhou, Henan China; 27 Department of Oncology, Affiliated Hospital of Hebei University, Baoding, Hebei China; 28 Department of Breast Oncology, the First Affiliated Hospital of Henan University of Science & Technology, Luoyang, Henan China; 29 Department of Oncology, Hangzhou Cancer Hospital, Hangzhou, Zhejiang China; 30 Department of Medical Oncology, Peking University First Hospital, Beijing, China; 31 Department of Breast Surgery III, Yunnan Cancer Hospital, Kunming, Yunnan China; 32 Department of Oncology, the Affiliated Hospital of Southwest Medical University, Luzhou, Sichuan China; 33 Department of Oncology, Jilin Cancer Hospital, Jilin, Changchun, China; 34 Department of Oncology, the People’s Hospital of Guangxi Zhuang Autonomous Region, Nanning, Guangxi China; 35 Department of Oncology, Zibo Central Hospital, Zibo, Shandong China; 36 Department of Breast Oncology, Peking University Cancer Hospital & Institute, Beijing, China; 37 Mammary gland, Linyi Cancer Hospital, Linyi, Shandong China; 38 Department of Radiation Oncology II, Gansu Provincial Cancer Hospital, Lanzhou, Gansu China; 39 Department of Breast Oncology, the Second Affiliated Hospital of Dalian Medical University, Dalian, Liaoning China; 40 Breast Center, the Fourth Hospital of Hebei Medical University, Shijiazhuang, Hebei China; 41 Department of Breast lymphatic, Henan Cancer Hospital, Zhengzhou, Henan China; 42 Department of Oncology, the Affiliated Hospital of Mongolia Medical University, Hohhot, Inner Mongolia China; 43 Department of Breast Oncology, Nanyang Second General Hospital, Nanyang, Henan China; 44 Department of Oncology, Anhui Provincial Hospital, Hefei, Anhui China; 45 Department of Breast Oncology, Cangzhou Hospital of Integrated TCM-WM Hebei, Cangzhou, Hebei China; 46 Department of Oncology, Luoyang Central Hospital, Luoyang, Henan China; 47 Breast Tumor Center, Chongqing Cancer Hospital, Chongqing, China; 48 Department of Breast Internal Medicine, The First Affiliated Hospital of Jinzhou Medical University, Jinzhou, Liaoning China; 49 Department of Radiotherapy Oncology, Lu’an Hospital of Traditional Chinese Medicine, Lu’an, Anhui China; 50 Breast Disease Treatment Center, Affiliated Hospital of Qingdao University, Qingdao, Shandong China; 51 Department of Oncology, the First Affiliated Hospital of Zhejiang University School of Medicine, Hangzhou, Zhejiang China; 52 Department of Internal Medicine-Oncology, Shao Yifu Hospital Affiliated to Zhejiang University School of Medicine, Hangzhou, Zhejiang China; 53 Department of Oncology, Qilu Hospital of Shandong University, Jinan, Shandong China; 54 Department of Breast Oncology, the Third Hospital of Nanchang, Nanchang, Jiangxi China; 55 Department of Oncology III, Mudanjiang Cancer Hospital, Mudanjiang, Heilongjiang China; 56 Department of Oncology, the Second Affiliated Hospital of Guangxi Medical University, Nanning, Guangxi China; 57 Department of Oncology, the Fifth Medical Center of Chinese PLA General Hospital, Beijing, China

**Keywords:** Breast cancer, Clinical trials

## Abstract

CDK2 is a principal mediator of CDK4/6 resistance. Concurrent CDK2/4/6 blockade may be effective in treating HR-positive, HER2-negative advanced breast cancer (ABC). This randomized, double-blind, parallel-controlled, phase 3 trial (ClinicalTrials.gov, NCT05375461) assessed the efficacy of culmerciclib, a CDK2/4/6 inhibitor, plus fulvestrant in ABC. Patients with HR-positive, HER2-negative, locally recurrent or metastatic breast cancer were randomized (2:1) to receive culmerciclib plus fulvestrant or matching placebo plus fulvestrant. Between March 18, 2022 and March 3, 2023, 293 pretreated patients (median age 53.0 years; pre- or perimenopausal 42.3%; bone metastasis 65.2%) were randomized to assigned treatments. At this prespecified interim analysis, culmerciclib plus fulvestrant extended the median investigator-assessed progression-free survival (PFS) significantly, the primary endpoint, as compared with placebo plus fulvestrant (16.6 months, 95% CI 13.8 to not evaluable *versus* 7.5 months, 95% CI 5.3 to 11.0; hazard ratio 0.36, 95% CI 0.26–0.51; stratified log rank test *P* < 0.001). Consistent effects were observed across diverse subgroups of patients. At a median follow-up duration of 13.8 months, overall survival was immature. The investigators-assessed objective response rate was 40.2% (95% CI, 33.3–47.5) for culmerciclib compared to 12.1% (95% CI 6.4–20.2) for placebo (stratified Mantel-Haenszel χ^2^ test *P* < 0.001). Diarrhea (87.1%) and neutropenia (80.4%) were the most common toxicities with culmerciclib plus fulvestrant. In conclusion, this randomized clinical trial met its primary outcome. Culmerciclib plus fulvestrant is well tolerated and leads to a significant gain in PFS of pretreated HR-positive HER2-negative ABC patients.

## Introduction

Breast cancer represents the most frequently diagnosed cancer among women around the world, with estimated 2.3 million new cases, and ranks as the fifth leading cause of cancer mortality, with 685,000 deaths in 2020.^1^ The disease poses a serious health concern in China as well, with over 400,000 estimated new cases and ~124,000 deaths in China in 2022.^2^ Particularly, hormone receptor-positive (HR^+^), human epidermal growth factor receptor 2-negative (HER2^-^) subtypes constitute about seventy percent of all breast cancer cases.^3^ Cyclin-dependent kinase 4 and 6 (CDK4/6), along with *D*-type cyclins, phosphorylate the retinoblastoma (Rb) tumor suppressor protein, resulting in the release of Rb-bound E2F, which causes transition from the G1 to S phase of the cell cycle.^4,5^ Aberrant CDK4/6 activation is a principal driver of endocrine resistance in HR^+^/HER2^-^ tumors. Several pivotal trials have also established the efficacy of CDK4/6 inhibitors (palbociclib, ribociclib, abemaciclib, and dalpiciclib) in later line settings, with an approximately fifty percent reduction in the risk of progression or death among patients with HR+, HER2- ABC with disease progression during or after endocrine therapy.^6–9^ Currently, a CDK4/6 inhibitor in combination with endocrine therapy remains the preferred treatment in the context of recurrent or metastatic disease.^10,11^ However, the disease eventually progresses in most patients with the appearance of acquired resistance to CDK4/6 inhibitors.^12,13^

Emerging evidence implicates CDK2 as a critical resistance mediator through compensatory activation following CDK4/6 inhibition,^11,13,14^ that involves multiple mechanisms including modification of the activities of MYC, CCNE1 and CDKN1A.^13,15,16^ CDK2 activation results in Rb hyperphosphorylation, setting up a positive feedback loop that sustains the expression of essential proteins for S phase.^17^ Nevertheless, CDK2 inhibitor monotherapy demonstrates minimal antitumor activity against breast cancer cells.^18^ Targeting of CDK2 by abemaciclib may have contributed to its greater efficacy and fewer hematologic toxicities in breast cancer patients, given the role of CDK2 activation in mediating resistance to CDK2/4/6 inhibitors.^14^ This paradox underscores the necessity for poly-pharmacological strategies combining CDK2/4/6 inhibition. In this context, culmerciclib (TQB3616), a first-in-class orally selective CDK2/4/6 inhibitor, showed promising growth inhibitory activities against breast cancer.^19^ Apart from being a potent CDK4-biased inhibitor over CDK6, culmerciclib showed notable inhibitory activities against CDK2, with an IC_50_ of 2.4 nM *vs*. 14.9 nM for abemaciclib,^20^ a CDK4/6 inhibitor with weak CDK2 inhibition.^6^ Given the functional redundancy of CDKs, simultaneous targeting of CDK2/4/6 and sparing CDK1 enables a polypharmacology approach while mitigating the safety risks of culmerciclib,^20^ suggesting that culmerciclib could be a more effective and safer CDK2/4/6 inhibitor. Several trials evaluated the pharmacokinetics and clinical activities of culmerciclib in HR^+^, HER2^-^ breast cancer (NCT03850873; NCT04796623; Chia Tai Tianqing data on file).

Herein, we present the protocol-specified interim analysis from our phase 3 trial assessing culmerciclib in combination with fulvestrant in ABC patients who had progressive disease after both endocrine therapy and chemotherapy. The study demonstrated that fulvestrant plus culmerciclib, a novel CDK2/4/6 inhibitor with distinct kinase selectivity, significantly improved PFS over fulvestrant plus placebo in Chinese patients with pretreated HR^+^/HER2^-^ ABC. Notably, culmerciclib demonstrated a manageable safety profile. These findings indicate that this combination could offer a promising therapeutic strategy for this population.

## Results

### Patient characteristics

**Fig. 1:**
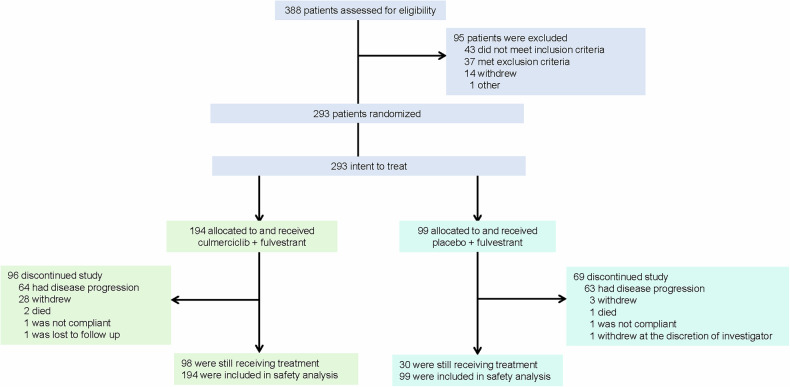
Patient disposition chart. Twenty-eight patients in the culmerciclib group (receiving other antitumor therapy [*n* = 4], voluntary ceasing treatment [*n* = 4], refusing visit [*n* = 6], withdrawal due to personal reasons [*n* = 4], subjective intolerabilities [*n* = 3], intolerable toxicities [*n* = 2], cancer pain [*n* = 5]) and three patients in the placebo group (receiving other antitumor therapy [*n* = 2] and withdrawal due to personal reason [*n* = 1] withdrew from the study

Between March 18, 2022 and March 3, 2023, of 388 screened patients, 293 were eligible; 194 received culmerciclib plus fulvestrant and 99 received placebo plus fulvestrant and constituted the intention-to-treat (ITT) population (Fig. 1). The patients had a median age of 53.0 years (Q1, Q3 47.0, 60.0); 14.0% (41/293) of the patients were aged ≥65 years and 42.3% (124/293) were pre- or perimenopausal. HER2 low expression (1+ or 2+/FISH−) was reported in 202 patients (68.9%). The majority of the patients (86.4% [258/293]) had a measurable lesion and 65.2% (191/293) had bone metastasis, including bone only metastasis in 55 patients (18.8%). In addition, 173 patients (59.0%) had visceral metastases, including lung metastases in 104 (35.5%) and liver metastases in 87 (29.7%). All patients had received 2^nd^ or later line therapy. All patients had received prior endocrine therapy, including aromatase inhibitors in 172 (58.7%), and 23.2% (68/293) had received systemic chemotherapy for recurrent/metastatic disease. Sixty-three patients (21.5%) had insensitivity to previous endocrine therapy, indicating primary endocrine therapy resistance. The two arms were well balanced in demographic and baseline characteristics (Table 1).

**Table 1:** Patient demographic and baseline characteristics

Variables	Culmerciclib plus fulvestrant *N* = 194	Placebo plus fulvestrant *N* = 99
Age, years		
Median (Q1,Q3)	53.0 (47.0, 61.0)	52.0 (46.0, 59.0)
Age distribution		
<65 years	165 (85.1)	87 (87.9)
≥65 years	29 (15.0)	12 (12.1)
Ethnicities		
Han Chinese	178 (91.8)	96 (97.0)
Others	16 (8.3)	3 (3.0)
Menopausal status at study entry^a^		
Postmenopausal	112 (57.7)	57 (57.6)
Premenopausal or perimenopausal	82 (42.3)	42 (42.4)
ECOG performance status score^b^		
0	103 (53.1)	44 (44.4)
1	91 (46.9)	54 (54.6)
Hormone receptor status, *n* (%)		
ER-positive	192 (99.0)	98 (99.0)
PR-positive	152 (78.4)	72 (72.7)
HER2 status^c^		
0	60 (30.9)	31 (31.3)
1+	71 (36.6)	29 (29.3)
2+	63 (32.5)	39 (39.4)
Measurable target lesions		
Yes	168 (86.6)	85 (85.9)
No	26 (13.4)	14 (14.1)
No. of metastatic organs		
<3	127 (65.5)	58 (58.6)
≥3	67 (34.5)	41 (41.4)
Visceral metastasis^a, d^		
No^e^	79 (40.7)	41 (41.4)
Yes	115 (59.3)	58 (58.6)
Lung	70 (36.1)	34 (34.3)
Liver	59 (30.4)	28 (28.3)
Bone metastasis		
No	71 (36.6)	31 (31.3)
Yes	123 (63.4)	68 (68.7)
Bone only	37(19.1)	18(18.2)
Sensitivity to previous endocrine therapies^a,f^		
No	41(21.1)	22(22.2)
Yes	153 (78.9)	77 (77.8)
As (neo)adjuvant endocrine therapy and endocrine therapy for recurrent/metastatic disease	12 (6.2)	11 (11.1)
As (neo)adjuvant endocrine therapy	138 (71.1)	72 (72.7)
As endocrine therapy for recurrent/metastatic disease	44 (22.7)	16 (16.2)
Previous endocrine therapy drugs		
Aromatase inhibitors	115 (59.3)	57 (57.6)
Selective estrogen receptor modulators	60 (31.0)	30 (30.3)
Aromatase inhibitors plus selective estrogen receptor modulators	19 (9.8)	12 (12.1)
Aromatase inhibitors as adjuvant endocrine therapy	96 (49.5)	53 (53.5)
Aromatase inhibitors as rescue endocrine therapy	49 (25.3)	26 (26.3)
Selective estrogen receptor modulators as adjuvant endocrine therapy	72 (37.1)	42 (42.4)
Selective estrogen receptor modulators as rescue endocrine therapy	17 (8.8)	10 (10.1)
Previous chemotherapy for recurrent/metastatic disease		
No	148 (76.3)	77 (77.8)
Yes	46 (23.7)	22 (22.2)

Data are expressed as number (%) unless otherwise specified

^a^Stratification factors

^b^Eastern Cooperative Oncology Group (ECOG) performance-status scores range from 0 (no disability) to 5 (death)

^c^HER2-negative status was defined as 0 or 1+ intensity on immunohistochemical testing, 2+ intensity on immunohistochemical testing and in-situ hybridization-negative, or in-situ hybridization-negative in the absence of immunohistochemical testing. Both HR and HER2 status were assessed centrally. Samples for assessing HR/HER2 status were primary tumors (123 patients), recurrent/metastatic lesions (151 patients) and both primary and recurrent/metastatic lesions (19 patients)

^d^Visceral metastasis refers to metastasis to organs in the thorax, abdomen or pelvis

^e^Includes metastasis to lymph nodes, breast, and cutaneous and soft tissues

^f^Patients were considered sensitive to prior endocrine therapy if they had a relapse after 24 months of adjuvant endocrine therapy or had a relapse after 6 months of first line endocrine therapy

Patient treatment characteristics are described in the Supplementary Information (Supplementary Table 1). At the interim analysis (data cutoff date, January 16, 2024), treatment was ongoing in 98 patients (50.5%) in the culmerciclib arm and 30 (30.3%) in the placebo arm. The median duration of treatment was 10.6 months with culmerciclib and 7.1 months with placebo. The median duration of treatment with fulvestrant was 12.0 months, with a median of 12 cycles (IQR 7, 16), in the culmerciclib arm and 8.0 months in the placebo arm, with a median of 8 cycles (IQR 3, 13). Ninety-six patients (49.5%) in the culmerciclib arm and 69 (69.7%) in the placebo arm discontinued treatment. Disease progression was the main reason for discontinuation and occurred in 64 patients in the culmerciclib arm and 63 in the placebo arm (Fig. 1).

### Efficacy measures

**Fig. 2:**
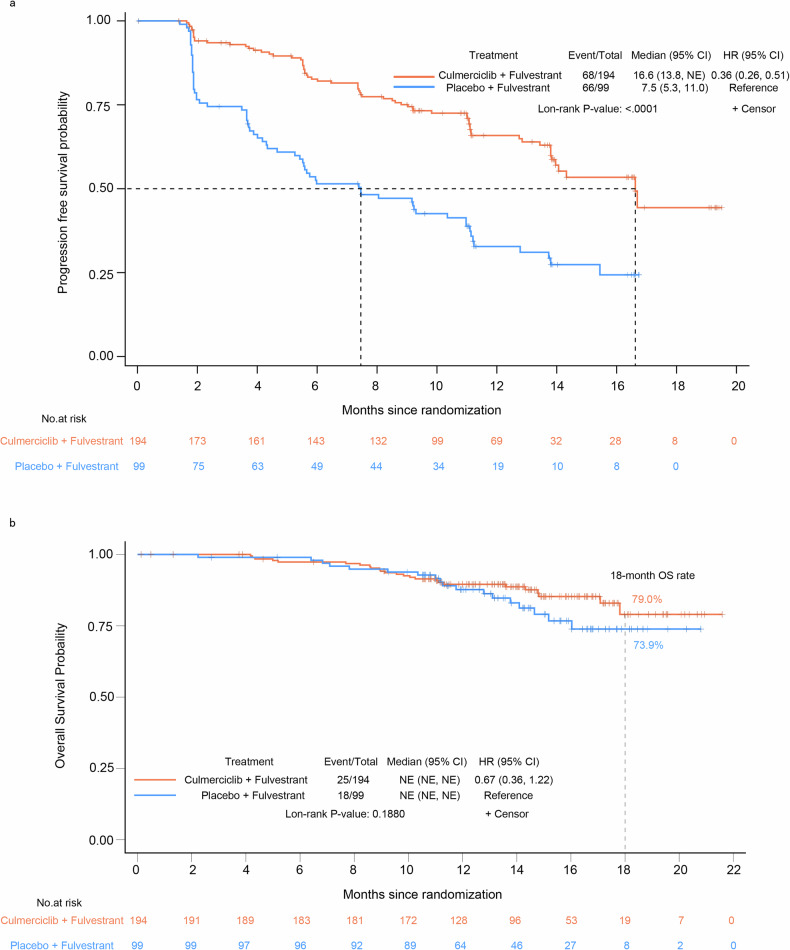
Survival outcomes. **a** Investigator-assessed progression-free survival (PFS) in the overall population. **b** Overall survival (OS) in the overall population. The hazard ratio was estimated with the use of the Cox proportional-hazards model with stratification according to visceral metastatic disease (yes vs. no), menopausal status (pre-, peri- or postmenopausal), and sensitivity to prior endocrine therapy (yes vs. no)

**Fig. 3:**
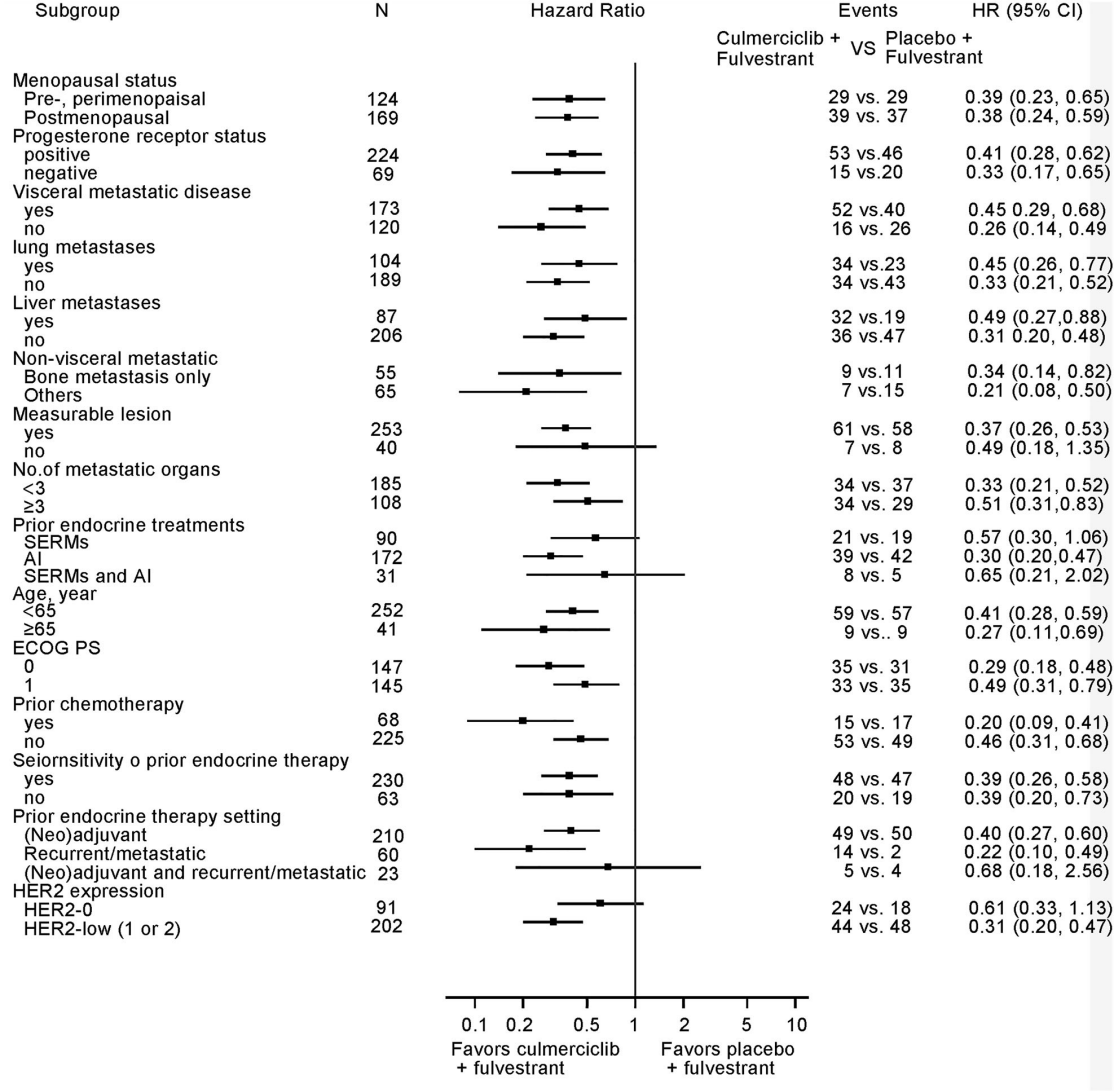
Prespecified subgroup analyses of investigator-assessed PFS in the overall population. Analysis of selected subgroups of interest within the overall population was performed at each subgroup level with the use of a Cox proportional-hazards model

At the interim analysis, 134 progression-free survival (PFS) events (68 with culmerciclib and 66 with placebo) had occurred. The investigators-assessed median PFS was 16.6 months (95% confidence interval [CI], 13.8 to not evaluable [NE]) in the culmerciclib arm *versus* 7.5 months (95% CI, 5.3–11.0) in the placebo arm (Fig. 2a and Table 2). The hazard ratio (HR) was 0.36 (95% CI, 0.26–0.51; stratified log rank test *P* < 0.001), meeting the primary study endpoint. The results of subgroup analyses of investigators-assessed PFS were concordant with the findings in the overall population and consistently favored culmerciclib over placebo across broad subgroups of the overall population (Fig. 3).

**Table 2:** Treatment response and survival outcomes of the overall population-ITT

	Culmerciclib plus fulvestrant *N* = 194	Placebo plus fulvestrant *N* = 99	*P*
Duration of follow-up, median (95% CI), months	13.8 (11.1, 13.8)	13.8 (11.2, 16.4)	
PFS assessed by investigators			
Median (95% CI), months	16.6 (13.8, NE)	7.5 (5.3, 11.0)	<0.001^a^
HR (95% CI)^b^	0.36 (0.26, 0.51)		
6-month PFS rate, % (95% CI)	82.7 (76.3, 87.5)	51.5 (41.1, 60.9)	
12-month PFS rate, % (95% CI)	65.9 (57.8, 72.7)	32.8 (23.1, 42.8)	
18-month PFS rate, % (95% CI)	44.4 (30.2, 57.6)	NE (NE, NE)	
PFS assessed by independent radiological committee			
Median (95% CI), months	NE (16.6, NE)	10.4 (7.4, NE)	
HR (95% CI)	0.42 (0.28, 0.62)		<0.001^a^
Objective response assessed by investigators^c^			
% (95% CI)^c^	40.2 (33.3, 47.5)	12.1 (6.4, 20.2)	
Odds ratio (95% CI)^f^	5.28 (2.66,10.47)		<0.001^h^
Best overall response, *n* (%)			
Complete response	5 (2.6)	0 (0.0)	
Partial response	73 (37.6)	12 (12.1)	
Stable disease	96 (49.5)	60 (60.6)	
Progressive disease	13 (6.7)	26 (26.3)	
Not evaluable^d^	0 (0.0)	0 (0.0)	
No assessment^e^	7 (3.6)	1 (1.0)	
Objective response among patients with a measurable lesion at baseline assessed by investigators^c^			
% (95% CI)^g^	46.4(38.7, 54.3)	14.1 (7.5, 23.4)	
Odds ratio (95% CI)^f^	5.46 (2.74, 10.86)		<0.0001^h^
Best overall response, *n* (%)			
Complete response	5 (3.0)	0 (0.0)	
Partial response	73 (43.5)	12 (14.1)	
Stable disease	76 (45.2)	49 (57.7)	
Progressive disease	10 (6.0)	23 (27.1)	
Not evaluable^d^	0 (0.0)	0 (0.0)	
No assessment^e^	4 (2.4)	1 (1.2)	
Duration of response assessed by investigators, median (95% CI), months	14.8 (12.0, NE)	NE (5.7, NE)	
HR (95% CI)^a^	1.55 (0.35, 6.83)		0.563^b^
Disease control assessed by investigators^c^			
% (95% CI)	89.7 (84.5, 93.6)	72.7 (62.9, 81.2)	
Odds ratio (95% CI)^f^	3.30 (1.73,6.29)		<0.001^h^
Clinical benefit assessed by investigators^c^			
% (95% CI)^g^	76.3 (69.7, 82.1)	50.5 (40.3, 60.7)	
Odds ratio (95% CI)^f^	3.17 (1.89, 5.31)		<0.001^h^
Overall survival (OS), months			
% (95% CI)	NE (NE, NE)	NE (NE, NE)	
HR (95% CI)^a^	0.67 (0.36, 1.22)		0.188^b^
6-month OS rate, % (95% CI)	97.4 (93.8, 98.9)	99.0 (93.1, 99.9)	
12-month OS rate, % (95% CI)	89.5 (84.1, 93.2)	87.7 (78.8, 93.0)	
18-month OS rate, % (95% CI)	79.0 (66.4, 87.3)	73.9 (60.5, 83.3)	

*BIRC* blinded independent radiological committee, *CI* confidence interval, *DCR* disease control rate, *HR* hazard ratio, *NE* not evaluable, *ORR* objective response rate, *OS* overall survival, *PFS* progression-free survival

^a^Stratified log rank test

^b^Stratified Cox proportional-hazards model. Stratifications factors include visceral metastatic disease (yes vs. no), menopausal status (pre-, peri- or postmenopausal), and sensitivity to prior endocrine therapy (yes vs. no)

^c^An objective response is defined as a complete response or a partial response and disease control is defined as a complete response, a partial response or a stable disease. Clinical benefit rate is the proportion of patients with an objective response [complete or partial] or stable disease as their best overall response lasting ≥24 weeks

^d^Patients had at least one postbaseline radiological evaluation but were not evaluable per RECIST, version 1.1 or other criteria, or less than 6 weeks had elapsed between randomization and complete response, partial response or stable disease

^e^Patients had no postbaseline radiological evaluation

^f^OR was estimated by logistic regression

^g^The confidence interval was calculated using exact binomial method

^h^Stratified Mantel-Haenszel χ^2^ test with stratification according to visceral metastatic disease (yes vs. no), menopausal status (pre-, peri- or postmenopausal), and sensitivity to prior endocrine therapy (yes vs. no)

The results of independent review committee (IRC)-assessed PFS highly aligned with the investigator-assessed analyses, with a 58% reduction in the risk of progression or death with culmerciclib over placebo (HR = 0.42, 95% CI, 0.28–0.62; stratified log rank test *P* < 0.001) (Supplementary Fig. 1). Consistent findings were observed in the PPS and supplemental analyses (Supplementary Table 2).

At a median follow-up duration of 13.8 months, the median overall survival (OS) was not reached in both arms, with a numerically higher 18-month OS rate in the culmerciclib arm than the placebo arm (79.0% *vs*. 73.9%; stratified log rank test *P* = 0.188) (Fig. 2b and Table 2).

The investigators-assessed objective response rate (ORR) was 40.2% (95% CI, 33.3–47.5) in the culmerciclib arm compared to 12.1% (95% CI 6.4–20.2) in the placebo arm (stratified Mantel–Haenszel χ^2^ test *P* < 0.001). The median DOR was non-statistically different in the two arms (14.8 months, 95% CI, 12.0 to NE *vs*. NE, 95% CI, 5.7 to NE; stratified log rank test *P* = 0.563) (Supplementary Fig. 1). The clinical benefit rate (CBR) was significantly higher in the culmerciclib arm than the placebo arm (76.3%, 95% CI, 69.7–82.1 vs. 50.5%, 95% CI, 40.3–60.7; stratified Mantel–Haenszel χ^2^ test *P* < 0.001) (Table 2).

Consistently, the IRC-assessed ORR was significantly higher in the culmerciclib arm than the placebo arm (35.6%, 95% CI, 28.8–42.7 vs. 13.1%, 95% CI, 7.2–21.4; stratified Mantel–Haenszel χ^2^ test *P* < 0.001) (Supplementary Table 3). The median duration of response was not reached in either arm (NE, 95% CI 14.8 to NE vs. NE, 95% CI 9.1 to NE; stratified HR 1.27, 95% CI 0.26–6.29, stratified Mantel–Haenszel χ2 test *P* = 0.766). Moreover, a significantly greater proportion of patients had clinical benefit in the culmerciclib arm than the placebo arm (74.2%, 95% CI 67.5 to 80.2 vs. 50.5%, 95% CI 40.3–60.7; stratified Mantel–Haenszel χ^2^ test *P* < 0.001) (Supplementary Table 3). In addition, among patients with a measurable lesion at baseline, the ORR was 46.4% (78/168; 95% CI 38.7–54.3) in the culmerciclib arm compared to 14.1% (12/85; 95% CI 7.5–23.4) in the placebo arm (stratified Mantel–Haenszel χ^2^ test *P* < 0.001).

### Safety

Adverse events (AEs) were assessed using the National Cancer Institute Common Terminology Criteria for Adverse Events (CTCAE), version 5.0. The safety set included 194 patients in the culmerciclib arm and 99 in the placebo arm. Treatment-emergent AE (TEAEs) of any grade occurred in 98.5% (191/194) patients in the culmerciclib arm and 96.0% (95/99) in the placebo arm. The most frequent TEAEs of any grade in the culmerciclib arm were diarrhea (87.1% vs. placebo 7.1%), neutropenia (80.4% *vs*. 17.2%), and leukopenia (79.9% vs. 22.2%) (Table 3). Grade ≥3 TEAEs occurred in 61.3% (119/194) in the culmerciclib arm and 20.2% (20/99) in the placebo arm. The most frequently reported grade 3 or worse TEAEs were neutropenia (culmerciclib 24.7% vs. placebo 4.0%), leukopenia (15.0% vs. 3.0%), hypokalemia (14.4% vs. 1.0%), and anemia (10.8% vs. 0%). TEAEs occurring in ≥10% of the patients in each arm are provided in Supplementary Table 4 by grades and the duration of grade 2 or worse TEAEs is provided in Supplementary Table 5.

**Table 3:** Summary of treatment-emergent adverse events (TEAEs) in the safety population

	Culmerciclib plus fulvestrant *N* = 194		Placebo plus fulvestrant *N* = 99	
	Any grade	Grade 3 or higher	Any grade	Grade 3 or higher
Any TEAEs	191 (98.5)	119 (61.3)	95 (96.0)	20 (20.2)
Serious AEs	36 (18.6)	32 (16.5)	10 (10.1)	7 (7.1)
TEAEs leading to dose reductions in culmerciclib/placebo	66 (34.0)	51 (26.3)	1 (1.0)	1 (1.0)
TEAEs leading to treatment interruptions	116 (59.8)	81 (41.8)	18 (18.2)	12 (12.1)
TEAEs leading to treatment discontinuation	1 (0.5)	—	0 (0.0)	—
TEAEs causing death	2 (1.0)		2 (2.0)	
Diarrhea	169 (87.1)	14 (7.2)	7 (7.1)	0 (0.0)
Neutropenia	156 (80.4)	48 (24.7)	17 (17.2)	4 (4.0)
Leukopenia	155 (79.9)	29 (15.0)	22 (22.2)	3 (3.0)
Anemia	124 (63.9)	21 (10.8)	12 (12.1)	0 (0.0)
Vomiting	117 (60.3)	4 (2.1)	7 (7.1)	0 (0.0)
Nausea	82 (42.3)	2 (1.0)	9 (9.1)	0 (0.0)
Alanine aminotransferase increase	74 (38.1)	5 (2.6)	29 (29.3)	0 (0.0)
Aspartate aminotransferase increase	74 (38.1)	9 (4.6)	31 (31.3)	0 (0.0)
Hypertriglyceridemia	74 (38.1)	14 (7.2)	30 (30.3)	2 (2.0)
Platelet count decrease	69 (35.6)	6 (3.1)	6 (6.1)	0 (0.0)
Hypokalemia	65 (33.5)	28 (14.4)	5 (5.1)	1 (1.0)
Hypercholesterolemia	57 (29.4)	0(0.0)	22 (22.2)	1 (1.0)
Lymphocyte count decrease	55 (28.4)	14 (7.2)	8 (8.1)	0 (0.0)
γ-glutamyl transferase increase	50 (25.8)	11 (5.7)	7 (7.1)	0 (0.0)
Body weight decrease	43 (22.2)	1 (0.5)	1 (1.0)	0 (0.0)
Hyperuricemia	43 (22.2)	1 (0.5)	12 (12.1)	0 (0.0)
Urinary tract infection	39 (20.1)	2 (1.0)	14 (14.1)	0 (0.0)
Lactate dehydrogenase increase	37 (19.1)	1 (0.5)	4 (4.0)	0 (0.0)
Blood creatinine increase	34 (17.5)	2 (1.0)	3 (3.0)	0 (0.0)
Asthenia	33 (17.0)	6 (3.1)	4 (4.0)	0 (0.0)
Blood alkaline phosphatase increase	32 (16.5)	4 (2.1)	8 (8.1)	0 (0.0)
Occult blood positive	31 (16.0)	0 (0.0)	13 (13.1)	0 (0.0)
Abdominal pain	27 (13.9)	1 (0.5)	0 (0.0)	0 (0.0)
Hypocalcemia	27 (13.9)	2 (1.0)	0 (0.0)	0 (0.0)
Decreased appetite	27 (13.9)	5 (2.6)	0 (0.0)	0 (0.0)
Hypoalbuminemia	25 (12.9)	0 (0.0)	6 (6.1)	0 (0.0)
Pyrexia	22 (11.3)	1 (0.5)	5 (5.1)	0 (0.0)
Sinus tachycardia	22 (11.3)	0 (0.0)	2 (2.0)	0 (0.0)
Hypophosphatasemia	21 (10.8)	1 (0.5)	1 (1.0)	0 (0.0)
Upper abdominal pain	20 (10.3)	0 (0.0)	0 (0.0)	0 (0.0)
Hyperglycemia	16 (8.3)	0 (0.0)	10 (10.1)	0 (0.0)

The safety population included all the patients who received at least one dose of culmerciclib, fulvestrant, or placebo. AEs in at least 10% of the patients for any grade in either group are reported regardless of the relationship to culmerciclib, fulvestrant, or placebo

Serious AEs occurred in 36 patients (18.6%) receiving culmerciclib plus fulvestrant and 10 (10.1%) in the placebo arm (Supplementary Table S4). Death due to TEAEs occurred in 2 patients (1.0%) in the culmerciclib arm (cerebral infarction and disease progression in 1 patient each) and in 2 patients in the placebo arm (disease progression in both patients). Deaths were not deemed to be treatment related according to the investigators.

TEAEs led to dose interruptions in 116 patients (59.8%) in the culmerciclib arm and 18 (18.2%) in the placebo arm. AEs led to dose reductions in 66 patients (34.0%) receiving culmerciclib versus 1 (1.0%) receiving placebo. One patient in the culmerciclib arm stopped treatment due to TEAEs and no patient in the placebo arm stopped treatment due to TEAEs.

Treatment-related AEs (TRAEs) are summarized in (Supplementary Tables 6, 7). TRAEs of any grade were reported in 98.5% (191/194) patients in the culmerciclib arm and 87.9% (87/99) in the placebo arm. Grade ≥3 TRAEs were reported in 57.7% (112/194) in the culmerciclib arm and 12.1% (12/99) in the placebo arm. TRAEs led to dose interruptions in 103 patients (53.1%) in the culmerciclib arm and 13 (13.1%) in the placebo arm. TRAEs led to dose reductions in 66 patients (34.0%) receiving culmerciclib versus 1 (1.0%) receiving placebo. No patient in either arm stopped treatment due to TRAEs and no death due to TRAEs was reported.

## Discussion

In this trial, the combination of culmerciclib and fulvestrant led to a clinically meaningful improvement, extending the median PFS of the ITT population by 9.1 months over placebo, with a 64% reduction in the risk of disease progression or death. These results confirm the study’s successful achievement of its primary endpoint. The PFS benefit was generally consistent across broad patient subgroups. At the time of interim analysis, OS data was immature. The concordant findings of the supplemental analyses, with the use of stratified and covariate-adjusted Cox proportional-hazards models, further ensured that the conclusions were robust and not dependent on mechanisms used to account for missing data.

The therapeutic potential of CDK2/4/6 inhibitors in HR^+^/HER2^-^ ABC patients with endocrine therapy resistance warrants further investigation. CDK2 plays a main role in cell cycle checkpoint from G1 to S and aberrant CDK2 activation has been identified as a key resistance mechanism to CDK4/6 inhibition in HR^+^ breast cancer.^11,13,14^ Patients with CDK2 activation responded poorly to palbociclib.^21^ This underscores CDK2 as a promising strategy to mitigate resistance.^14^ Notably, effective targeting may require polypharmacology approaches, while CDK2 inhibition alone exhibits limited antitumor activity.^18^ co-inhibition of CDK2/4/6 could synergistically overcome resistance mechanisms. Abemaciclib, a weak inhibitor of CDK2, when added to fulvestrant, led to a 7.1-month extension in PFS in women with HR^+^/HER2^-^ ABC who had progressed.^6^ Intriguingly, abemaciclib retains efficacy in palbociclib-/ribociclib-resistant models,^22^ hinting at CDK2 inhibition’s potential contributory role. As a CDK4-biased inhibitor with dual CDK2/4/6 activity,^23^ simultaneous pathway blockade by culmerciclib may provide a potential explanation for the 9.1-month PFS prolongation over placebo demonstrated in this trial.

Our findings resonate with several pivotal trials yet reveal distinct features. Similar to MONARCH 2^6^(25.3% vs 21.5% primary endocrine resistance rates), both studies enrolled patients with ≤1 prior endocrine therapy line. Notably, while MONARCH 2 and MONALEESA-3^7^ excluded advanced-stage chemotherapy recipients, 23.2% of our cohort received chemotherapy for recurrent/metastatic disease. In PALOMA-3,^8,24^ palbociclib plus fulvestrant achieved a 6.6-month PFS advantage versus placebo. Caution should be exercised for cross-trial comparisons due to population heterogeneity and trial design variations.

Subgroup analyses revealed consistent benefits. Prior aromatase inhibitor recipients derived substantial benefit (HR 0.30, 95% CI 0.20–0.47), a population enriched for *ESR1* mutations implicated in endocrine resistance.^25^ Similarly, PR-negative patients (HR 0.33, 95% CI 0.17–0.65) and HER2-low expressors (HR 0.31, 95% CI 0.20–0.47) showed pronounced responses, aligning with evidence that PR loss^26^ and HER2-low status^27^ correlate with CDK4/6 inhibitor resistance. Even primary endocrine-resistant subgroups benefited (HR 0.39, 95% CI 0.20–0.73), notably challenging given their traditionally poor prognosis.^28^

The safety profile of culmerciclib/fulvestrant proved manageable, with most AEs in the culmerciclib arm being mild or moderate, and no new safety concerns emerged. Despite targeting multiple kinases, culmerciclib showed an overall acceptable safety profile compared to other CDK4/6 inhibitors with regards to ≥grade 3 TEAEs, indicating that CDK2 inhibition did not increase toxicities of culmerciclib. Similar to abemaciclib, diarrhea was the most frequent TEAE with culmerciclib (87.1% and abemaciclib 86.4%).^6^ Overall, diarrhea was managed with antidiarrheal medication, or by dose reductions or omission. No patients stopped treatment due to diarrhea in this trial. The rates of hematologic toxicities with culmerciclib were comparable with abemaciclib for Chinese patients.^29^ The rate of grade 3 or 4 neutropenia was the lowest with culmerciclib among CDK4/6 inhibitors and no febrile neutropenia occurred, likely because that culmerciclib is a potent CDK4-biased inhibitor over CDK6, thereby leading to less prominent hematologic toxicities.^11^ Though Asian patients were significantly more likely to have neutropenia.^30,31^ culmerciclib had a low rate of grade 3 or 4 neutropenia (24.7%).

The study has several limitations. The placebo arm included patients who were treated with fulvestrant only and the trial did not include patients who had received standard therapy with CDK4/6 inhibitors. This population is similar to that in the PALOMA 3 trial^32^ of palbociclib. Palbociclib is associated with mechanism-based hematologic toxicities, fatigue, nausea, and an increased risk of infection. Culmerciclib, apart from CDK4/6, targets CDK2 and has distinct binding kinetics for CDK2/4/6. The actual merits of culmerciclib needs to be demonstrated in patients who have failed current standard of care. A phase 2 trial (NCT06702618) is ongoing that explores the efficacy of culmerciclib in recurrent/metastatic breast cancer patients who received prior CDK4/6 inhibitor therapy. Furthermore, biomarker analyses were omitted, limiting insights into molecular determinants of response. In addition, the modest size of the trial population did not allow meaningful analyses of certain subgroups. At the data cutoff date, OS data was immature. The OS results will be reported separately upon maturity.

In conclusion, fulvestrant plus culmerciclib, a novel CDK2/4/6 inhibitor with distinct kinase selectivity, significantly improved PFS over fulvestrant plus placebo in Chinese patients with pretreated HR^+^/HER2^-^ ABC. Notably, culmerciclib demonstrated a manageable safety profile. These findings indicate that this combination could offer an effective therapeutic strategy for this population. Given that the trial did not include patients who had received prior therapy with CDK4/6 inhibitors, future studies should further evaluate the efficacy of culmerciclib in patients previously treated with CDK4/6 inhibitors.

## Methods

### Ethic statement

The trial was undertaken in accordance with the provisions of the Declaration of Helsinki and the International Conference on Harmonisation guidelines for Good Clinical Practice. The study has received approval from the lead institutions, the Fifth Medical Center of Chinese PLA General Hospital (No: 2021-11-39-3), the Jiangsu Province Hospital (No: 2021-MD-180.A3), as well as the ethics committees of each participating center. All patients provided written informed consent before enrollment. The trial, along with its subsequent amendments, is registered with clinicatrials.gov (NCT05375461). The study protocol adhered to the SPIRIT statement^33^ and the reporting of the study adhered to the CONSORT statement.^34^ An independent data and safety monitoring committee assessed the progress of the trial and reviewed the efficacy and safety data.

### Trial design and patients

This randomized, double-blind, parallel-controlled, phase 3 trial conducted at 56 participating centers across China enrolled pre-, peri- or postmenopausal women (between 18 and 75 years of age) with histologically proven HR^+^/HER2^-^, locally recurrent or metastatic breast cancer that was not amenable to curative resection or radiotherapy, or clinically not indicated for chemotherapy. HR^+^ status was defined as positive (≥10%) estrogen receptor expression or progesterone receptor expression. Patients should have a measurable lesion according to the Response Evaluation Criteria in Solid Tumors (RECIST), version 1.1, or bone only metastasis including lytic or mixed lytic-blastic bone lesions. HER2^-^ status was defined as 0 or 1+ intensity on immunohistochemical testing, 2+ intensity on immunohistochemical testing and in-situ hybridization-negative, or in-situ hybridization-negative in the absence of immunohistochemical testing. Both HR and HER2 status were assessed centrally. Patients were considered sensitive to prior endocrine therapy if they had a relapse after 24 months of adjuvant endocrine therapy or had a relapse after 6 months of first line endocrine therapy. Patients were considered to have visceral metastasis if they had metastasis to organs in the thorax, abdomen or pelvis.

Patients were required to have adequate bone marrow and organ function and an Eastern Cooperative Oncology Group (ECOG) performance status of 0 or 1. Patients should have relapse or progression during or within 12 months after the end of adjuvant endocrine therapy without subsequent endocrine therapy, or progression after rescue endocrine therapy after an initial diagnosis of locally advanced or metastatic disease or >12 months after the end of adjuvant endocrine therapy. Patients must have received no more than one line of endocrine therapy or one line of chemotherapy for recurrent or metastatic disease. Adjuvant endocrine therapy should last for at least one year and relapse or disease progression had to be radiologically confirmed. Patients should have received no more than one line of systemic chemotherapy or endocrine therapy in the context of recurrent or metastatic disease. Patients should have a left ventricular ejection fraction (LVEF) of no less than 50%. Principal exclusion criteria were central nervous system metastasis, carcinomatous meningitis or leptomeningeal disease. The eligibility criteria are detailed in the trial protocol (p 40-41).

### Randomization and treatments

Randomization was done using a central interactive, web-based randomization scheme and stratified according to visceral metastatic disease (yes vs. no), menopausal status (pre-, peri- or postmenopausal), and sensitivity to prior endocrine therapy (yes vs. no). Using a 2:1 ratio, we randomly assigned patients to receive culmerciclib (180 mg, orally, once daily [QD] for a 4-week cycle) plus fulvestrant (500 mg, administered as an intramuscular injection, on day 1 and 15 of cycle 1 and day 1 of subsequent cycles) or placebo plus fulvestrant. Pre- or perimenopausal women received a gonadotropin-releasing hormone agonist for continuous ovarian function suppression throughout the study. Two dose reductions of culmerciclib or placebo were allowed. All patients and investigators were blind to assigned treatment. Treatment continued until disease progression, intolerable toxicities, or at the discretion of the investigators.

### Assessments and study endpoints

Tumors were evaluated by computed tomography or magnetic resonance imaging by investigators and an independent radiological committee (IRC) per RECIST, version 1.1, within 4 weeks before study enrollment, every 8 weeks for the first 48 weeks, and every 12 weeks thereafter. Bone scintigraphy was undertaken every 24 weeks in patients with baseline bone lesion and when clinically indicated in those without. Hematologic and blood laboratory tests and 12-lead electrocardiography were performed within 7 days before study enrollment and at each visit through the treatment period.

AEs were monitored throughout the treatment period and until 4 weeks after the final dose of study medications and graded using CTCAE, version 5.0. AEs were coded to a preferred term using the latest MedDRA. The incidence of AEs, serious AEs and laboratory abnormalities were recorded.

The primary endpoint was investigator-confirmed PFS, which was calculated from the date of randomization to disease progression or death of any cause, whichever occurred earlier. The secondary endpoints included IRC-confirmed PFS, OS, calculated from the date of randomization to death of any cause; ORR, which was defined as the percentage of patients who had complete or partial response as their best overall response as assessed by investigators per RECIST, version 1.1; duration of response (DOR), which was calculated from the first documented complete or partial response to the first documented disease progression or death of any cause, whichever occurred earlier; and CBR, which was the proportion of patients with complete or partial response or stable disease as their best overall response lasting ≥24 weeks.

### Statistical analysis

The number of patients required for this trial was based on the primary end point of investigator-assessed PFS and was calculated with the use of a predefined stratified log-rank test. Assuming a median PFS of 6.0 months for placebo plus fulvestrant, 186 events of progression or death would be required in the two treatment arms for the study to have 90% power to detect an HR of 0.60 with a two-sided significance level of α = 0.05. A total population of 243 patients was required. Assuming an attrition rate of 15%, at least 287 patients (191 for the fulvestrant plus culmerciclib arm and 96 for the fulvestrant plus placebo arm) were anticipated. The primary endpoint was to be analyzed at the interim analysis at ~70% maturity in the overall population when 131 events of progression or death had occurred and at the final analysis at 100% maturity when 186 events of progression or death had occurred. Type I errors were controlled using the Lan-DeMets spending function approximating O’Brien-Fleming boundary.^35^ For statistical significance for difference between culmerciclib plus fulvestrant *vs*. placebo plus fulvestrant, two-sided nominal *P* values were calculated to be <0.01477 and *P* < 0.04551 at the prespecified interim analysis and the final analysis, respectively, and adjusted to 0.01654 and 0.04501, respectively, at the interim analysis.

The study followed the ITT principle. Efficacy analyses were based on the ITT population which encompassed all the patients who had undergone randomization. The Per Protocol Set (PPS) consisted of all randomized patients who received at least one dose of the study medications as planned per protocol and had no major protocol violations. The results of the analysis of the PPS are provided briefly to show consistency. The Kaplan–Meier method was used to estimate median PFS, OS, and duration of response and 95% CI, and tested with a log-rank test, with stratification according to visceral metastatic disease (yes vs. no), menopausal status (pre-, peri- or postmenopausal), and sensitivity to prior endocrine therapy (yes vs. no). HR and associated 95% CI were calculated from a stratified Cox proportional-hazards model. The between-arm comparisons for objective response and clinical benefit rates were implemented using the stratified Mantel–Haenszel method and the odds ratio (OR) was estimated by logistic regression.

Sensitivity analyses for PFS were carried out as specified in the statistical analysis plan and a hypothetical estimand strategy was used to account for intercurrent events. Subgroup analyses of PFS were performed by menopausal status (pre- or perimenopausal vs. postmenopausal), age (<65 years or ≥65 years), ECOG performance status (0 vs. 1), progesterone receptor status (positive vs. negative), HER2 expression (HER0 vs. HER2-low), visceral metastatic disease (measurable lesion (yes vs. no), visceral metastatic disease (yes vs. no), lung metastasis (yes vs. no), liver metastasis (yes vs. no), non-visceral metastases (bone metastasis *vs*. others), number of metastatic sites (<3 vs. ≥3), prior endocrine treatments (selective estrogen receptor modulators vs. aromatase inhibitors vs. selective estrogen receptor modulators and aromatase inhibitors) prior endocrine therapy setting ([neo]adjuvant vs, recurrent/metastatic vs. [neo]adjuvant and recurrent/metastatic) and sensitivity to prior endocrine therapy (yes vs. no), and prior chemotherapy (yes vs. no).

The safety set consisted of all patients who received at least one dose of study medication and underwent at least one postbaseline safety evaluation. Safety data was analyzed mainly using descriptive statistics.

SAS (version 9.4; SAS Institute, Cary, NC, USA) was used for statistical analyses. This study is registered with ClinicalTrials.gov, NCT05375461.

## Supplementary information

Supplementary Tables and Figures

Protocol

SAP

CONSORT_2025_checklist

## Data Availability

The protocol, statistical analysis plan, and other relevant study materials are publicly available online. The datasets generated and analyzed during the current study are not publicly available due to ethical and regulatory restrictions but may be made available from the corresponding authors upon reasonable request. De-identified participant data may be shared following approval of a proposal that includes a detailed description of study objectives and a statistical analysis plan, which will be reviewed by all corresponding authors.
