# Supplementary material for: Novel CDK2/4/6 inhibitor culmerciclib (TQB3616) plus fulvestrant in previously treated, HR-positive, HER2-negative advanced breast cancer: a randomized, double-blind, phase 3 trial
Source: Signal Transduct Target Ther. 2025 Dec 18;10:414. doi: 10.1038/s41392-025-02475-6 (PMC12714862; doi:10.1038/s41392-025-02475-6)
Supplement: Supplementary file 1 — Supplementary Tables and Figures [file 41392_2025_2475_MOESM1_ESM.docx]

Supplementary Materials for

**Novel CDK2/4/6 inhibitor culmerciclib (TQB3616) plus fulvestrant in previously treated, HR-positive, HER2-negative advanced breast cancer: a randomized, double-blind, phase 3 trial**

Yongmei Yin†*, Qingyuan Zhang†, Tao Sun, Chunfang Hao, Zhihong Wang, Jin Yang, Yongsheng Wang, Yanxia Shi, Jing Sun, Quchang Ouyang, Haichuan Su, Jinsheng Wu, Lu Gan, Meng Han, Liming Gao, Xiaojia Wang, Bing Zhao, Hui Li, Jiuda Zhao, Hongwei Yang, Fangling Ning, Fuguo Tian, Juliang Zhang, Hongmei Sun, Zhaofeng Niu, Hong Zong, Aimin Zang, Xinshuai Wang, Xinyu Qian, Shikai Wu, Jianyun Nie, Lijia He, Ying Cheng, Yanrong Hao, Yi Zhai, Huiping Li, Jingfen Wang, Shihong Wei, Man Li, Yunjiang Liu, Hongqiang Guo, Qun Hu, Lina Liu, Xinghua Han, Ruizhen Luo, Mingli Ni, Xianjun Tang, Zhenhua Zhai, Meiqian Ding, Haibo Wang, Peng Shen, Xian Wang, Lian Liu, Wenyan Chen, Gang Liu, Zhengwen Cai, Zefei Jiang*

Correspondence to: ym.yin@hotmail.com, jiangzefei@csco.org.cn

**This document includes:**

Supplementary Figure 1

Supplementary Tables 1 to 7

**Supplementary Figure1**


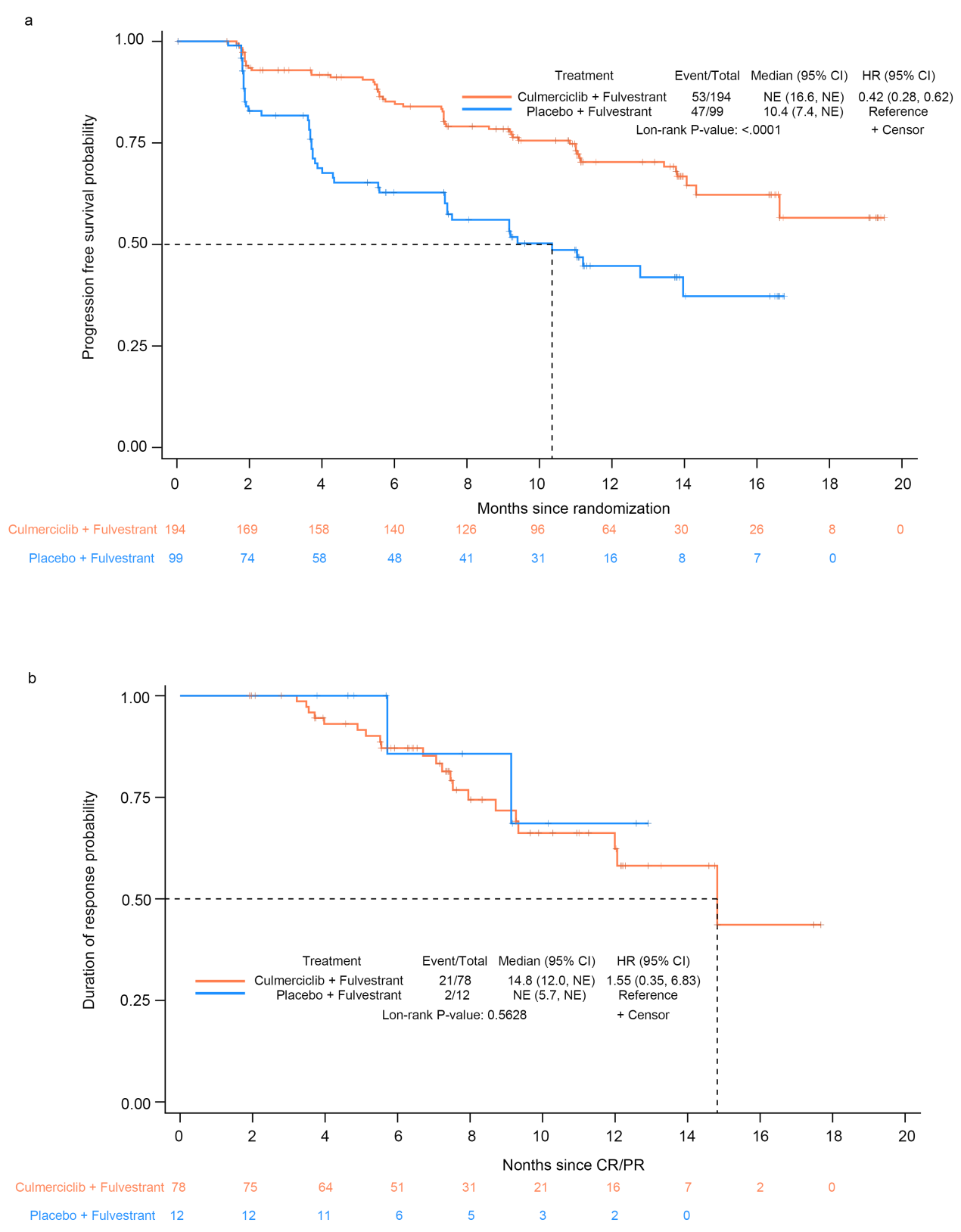


**Figure S1.** (a) Independent radiological committee (IRC)-assessed progression-free survival (PFS) in the overall population. The hazard ratio was estimated with the use of the Cox proportional-hazards model with stratification according to visceral metastatic disease (yes *vs.* no), menopausal status (pre-, peri- or postmenopausal), and sensitivity to prior endocrine therapy (yes *vs.* no). Tick marks indicate censored data. (b) Investigator-assessed duration of response in the overall population.

Independent radiological committee (IRC)-assessed progression-free survival (PFS) in the overall population. The hazard ratio was estimated with the use of the Cox proportional-hazards model with stratification according to visceral metastatic disease (yes *vs.* no), menopausal status (pre-, peri- or postmenopausal), and sensitivity to prior endocrine therapy (yes *vs.* no). Tick marks indicate censored data. (b) Investigator-assessed duration of response in the overall population.

**Supplementary table 1**: Treatment Characteristics of the Study Patients in the ITT Population.

|  | Culmerciclib plus fulvestrant | Placebo plus fulvestrant |
| --- | --- | --- |
| Culmerciclib or placebo | **N=194** | **N=99** |
| Median exposure duration (IQR), months | 10.6(5.5, 13.6) | 7.1(2.3, 11.9) |
| Mean (SD) exposure, mg/day | 166.4(17.4) | 178.1(3.9) |
| Relative dose intensity (%) |  |  |
| Mean (SD) | 92.4(9.7) | 98.9(2.2) |
| <80% | 18 (9.3) | 0 (0.0) |
| 80%-120% | 176 (90.7) | 98 (99.0) |
| >120% | 0 (0.0) | 0 (0.0) |
| Fulvestrant |  |  |
| Median exposure duration (IQR), months | 12.0(7.0,16.0) | 8.0(3.0,13.0) |
| Mean (SD) exposure, mg/day | 499.6(4.9) | 500.0(0.0) |
| Relative dose intensity (%) |  |  |
| Mean (SD) | 99.9(1.0) | 100.0(0.0) |
| <80% | 0 (0.0) | 0 (0.0) |
| 80%-120% | 194 (100.0) | 99 (100.0) |
| >120% | 0 (0.0) | 0 (0.0) |
| Data are expressed as number (%) unless otherwise specified.  Relative drug intensity is defined as the actual dose received divided by the preset dose in a treatment group. | | |

**Supplementary table 2**: Supplemental analyses of progression-free survival (PFS).

|  | Culmerciclib plus fulvestrant | Placebo plus fulvestrant | *P* |
| --- | --- | --- | --- |
| Estimand 2-1^¶^, FAS | N=194 | N=99 |  |
| Number of events, n (%) | 44 (22.7) | 53 (53.5) |  |
| Censored events, n (%) | 150 (77.3) | 46 (46.5) |  |
| PFS assessed by investigators |  | |  |
| Median (95% CI), months | 16.6 (13.8, NE) | 7.39 (4.01, 11.0) |  |
| HR (95% CI) ^‖^ | 0.34 (0.23, 0.52) | | <0.001^**^ |
| 6-month PFS rate, % (95% CI) | 83.3 (75.7, 88.7) | 50.7 (39.2, 61.2) |  |
| 12-month PFS rate, % (95% CI) | 64.8 (54.4, 73.4) | 30.3 (19.1, 42.3) |  |
| 18-month PFS rate, % (95% CI) | 47.8 (29.6, 63.9) | NE (NE, NE) |  |
| Estimand 2-2‡‡, FAS |  |  |  |
| Number of events, n (%) | 52 (26.8) | 57 (57.6) |  |
| Censored events, n (%) | 142 (73.2) | 42 (42.4) |  |
| PFS assessed by investigators |  |  |  |
| Median (95% CI), months | 16.6 (13.8, NE) | 7.39 (4.0, 11.0) |  |
| HR (95% CI) ^‖^ | 0.37 (0.25, 0.54) | | <0.001^**^ |
| 6-month PFS rate, % (95% CI) | 82.5 (75.3, 87.7) | 51.21 (40.1, 61.2) |  |
| 12-month PFS rate, % (95% CI) | 64.8 (55.5, 72.7) | 32.10 (21.7, 43.0) |  |
| 18-month PFS rate, % (95% CI) | 46.9 (29.4, 62.7) | NE (NE, NE) |  |
| Estimand 3^§§^, PPS | N=183 | N=96 |  |
| Number of events, n (%) | 62 (33.9) | 64 (66.7) |  |
| Censored events, n (%) | 121 (66.1) | 32 (33.3) |  |
| PFS assessed by investigators |  |  |  |
| Median (95% CI), months | NE (14.0, NE) | 7.4 (5.3, 11.0) |  |
| HR (95% CI) ^‡^ | 0.36 (0.25, 0.51) | | <0.001^**^ |
| 6-month PFS rate, % (95% CI) | 82.3 (75.6, 87.3) | 51.06 (40.4, 60.6) |  |
| 12-month PFS rate, % (95% CI) | 65.9 (57.6, 73.0) | 31.5 (21.7, 41.7) |  |
| 18-month PFS rate, % (95% CI) | 51.0 (38.2, 62.4) | NE (NE, NE) |  |
| ^¶^Patients with culmerciclib interruptions ＞7 days due to the COVID pandemic were censored.  ^**^ Stratified log rank test.  †† Covariate-adjusted cox proportional-hazards model. Covariates include visceral metastatic disease (yes *vs.* no), menopausal status (pre-, peri- or postmenopausal), and sensitivity to prior endocrine therapy. (yes *vs.* no).  ^‡‡^ Patients with culmerciclib plus fulvestrant interruptions ＞7 days due to the COVID pandemic were censored.  ^§§^ PPS. | | | |

**Supplementary table 3**: Treatment response of the overall population per IRC

|  | Culmerciclib plus fulvestrant  N=194 | Placebo plus fulvestrant  N=99 | *P* |
| --- | --- | --- | --- |
| Objective response assessed by IRC^‡^ |  |  |  |
| % (95% CI) ^††^ | 35.6 (28.8, 42.7) | 13.1 (7.2, 21.4) |  |
| Odds ratio (95% CI)** | 3.74 (1.93,7.23) | | <0.001^§^ |
| Best overall response, n(%) |  |  |  |
| Complete response | 1 (0.5) | 0 (0.0) |  |
| Partial response | 68 (35.1) | 13 (13.1) |  |
| Stable disease | 104 (53.6) | 68 (68.7) |  |
| Progressive disease | 13 (6.7) | 17 (17.2) |  |
| Not evaluable^¶^ | 0 (0.0) | 0 (0.0) |  |
| No assessment^‖^ | 8 (4.1) | 1 (1.0) |  |
| Duration of response assessed by IRC, median (95% CI), months | NE (14.8, NE) | NE (9.1, NE) |  |
| HR (95% CI) ^†^ | 1.27 (0.26-6.29) | | 0.766 ^§^ |
| Disease control assessed by IRC^‡^ |  |  |  |
| % (95% CI) | 89.2 (83.9, 93.2) | 81.8 (72.8, 88.9) |  |
| Odds ratio (95% CI)** | 1.83 (0.92,3.63) | | 0.0821^§^ |
| Clinical benefit assessed by IRC^‡^ |  |  |  |
| % (95% CI) ^††^ | 74.2 (67.5, 80.2) | 50.5 (40.3, 60.7) |  |
| Odds ratio (95% CI)** | 2.83 (1.70,4.71) | | <0.001^§^ |
| Abbreviations: IRC, independent radiological committee; CI, confidence interval NE, not evaluable.  ^†^ Stratified Cox proportional-hazards model. Stratifications factors include visceral metastatic disease (yes *vs.* no), menopausal status (pre-, peri- or postmenopausal), and sensitivity to prior endocrine therapy (yes *vs.* no).  †Covariate-adjusted cox proportional-hazards model (estimand 1). Covariates include visceral metastatic disease (yes *vs.* no), menopausal status (pre-, peri- or postmenopausal), and sensitivity to prior endocrine therapy (yes *vs.* no).  ^‡^An objective response is defined as a complete response or a partial response and disease control is defined as a complete response, a partial response or a stable disease. Clinical benefit rate is the proportion of patients with an objective response [complete or partial] or stable disease as their best overall response lasting ≥24 weeks.  ^§^ Stratified Mantel-Haenszel χ^2^ test with stratification according to visceral metastatic disease (yes *vs.* no), menopausal status (pre-, peri- or postmenopausal), and sensitivity to prior endocrine therapy (yes *vs.* no).  ^¶^Patients had at least one postbaseline radiological evaluation but were not evaluable per RECIST, version 1.1 or other criteria, or less than 6 weeks had elapsed between randomization and complete response, partial response or stable disease.  ^‖^Patients had no postbaseline radiological evaluation.  ** OR was estimated by logistic regression.  ^††^The confidence interval was calculated using exact binomial method. | | | |

**Supplementary table 4:** Treatment-emergent adverse events (TEAEs) according to grades in the safety population

|  | **Culmerciclib plus fulvestrant**  **N=194** | | | | | **Placebo plus fulvestrant**  **N=99** | | | | |
| --- | --- | --- | --- | --- | --- | --- | --- | --- | --- | --- |
| Grade | 1 | 2 | 3 | 4 | 5 | 1 | 2 | 3 | 4 | 5 |
| Preferred terms |  |  |  |  |  |  |  |  |  |  |
| Diarrhea | 76(39.18) | 79(40.72) | 14(7.22) | 0(0.00) | 0(0.00) | 7(7.07) | 0(0.00) | 0(0.00) | 0(0.00) | 0(0.00) |
| Neutropenia | 23(11.86) | 85(43.81) | 44(22.68) | 4(2.06) | 0(0.00) | 4(4.04) | 9(9.09) | 3(3.03) | 1(1.01) | 0(0.00) |
| Leukopenia | 34(17.53) | 92(47.42) | 28(14.43) | 1(0.52) | 0(0.00) | 11(11.11) | 8(8.08) | 3(3.03) | 0(0.00) | 0(0.00) |
| Anemia | 47(24.23) | 56(28.87) | 21(10.82) | 0(0.00) | 0(0.00) | 9(9.09) | 3(3.03) | 0(0.00) | 0(0.00) | 0(0.00) |
| Vomiting | 38(19.59) | 75(38.66) | 4(2.06) | 0(0.00) | 0(0.00) | 3(3.03) | 4(4.04) | 0(0.00) | 0(0.00) | 0(0.00) |
| Nausea | 54(27.84) | 26(13.40) | 2(1.03) | 0(0.00) | 0(0.00) | 4(4.04) | 5(5.05) | 0(0.00) | 0(0.00) | 0(0.00) |
| Alanine aminotransferase increase | 61(31.44) | 8(4.12) | 5(2.58) | 0(0.00) | 0(0.00) | 27(27.27) | 2(2.02) | 0(0.00) | 0(0.00) | 0(0.00) |
| Aspartate aminotransferase increase | 59(30.41) | 6(3.09) | 9(4.64) | 0(0.00) | 0(0.00) | 26(26.26) | 5(5.05) | 0(0.00) | 0(0.00) | 0(0.00) |
| Hypertriglyceridemia | 39(20.10) | 21(10.82) | 10(5.15) | 4(2.06) | 0(0.00) | 16(16.16) | 12(12.12) | 1(1.01) | 1(1.01) | 0(0.00) |
| Platelet count decrease | 48(24.74) | 15(7.73) | 3(1.55) | 3(1.55) | 0(0.00) | 4(4.04) | 2(2.02) | 0(0.00) | 0(0.00) | 0(0.00) |
| Hypokalemia | 34(17.53) | 3(1.55) | 20(10.31) | 8(4.12) | 0(0.00) | 3(3.03) | 1(1.01) | 1(1.01) | 0(0.00) | 0(0.00) |
| Hypercholesterolemia | 53(27.32) | 4(2.06) | 0(0.00) | 0(0.00) | 0(0.00) | 21(21.21) | 0(0.00) | 1(1.01) | 0(0.00) | 0(0.00) |
| Lymphocyte count decrease | 21(10.82) | 20(10.31) | 14(7.22) | 0(0.00) | 0(0.00) | 5(5.05) | 3(3.03) | 0(0.00) | 0(0.00) | 0(0.00) |
| γ-glutamyl transferase increase | 24(12.37) | 15(7.73) | 9(4.64) | 2(1.03) | 0(0.00) | 6(6.06) | 1(1.01) | 0(0.00) | 0(0.00) | 0(0.00) |
| Body weight decrease | 20(10.31) | 22(11.34) | 1(0.52) | 0(0.00) | 0(0.00) | 1(1.01) | 0(0.00) | 0(0.00) | 0(0.00) | 0(0.00) |
| Hyperuricemia | 42(21.65) | 0(0.00) | 1(0.52) | 0(0.00) | 0(0.00) | 12(12.12) | 0(0.00) | 0(0.00) | 0(0.00) | 0(0.00) |
| Urinary tract infection | 11(5.67) | 26(13.40) | 2(1.03) | 0(0.00) | 0(0.00) | 6(6.06) | 8(8.08) | 0(0.00) | 0(0.00) | 0(0.00) |
| Blood lactate dehydrogenase increase | 36(18.56) | 0(0.00) | 1(0.52) | 0(0.00) | 0(0.00) | 4(4.04) | 0(0.00) | 0(0.00) | 0(0.00) | 0(0.00) |
| Blood creatinine increase | 27(13.92) | 5(2.58) | 2(1.03) | 0(0.00) | 0(0.00) | 3(3.03) | 0(0.00) | 0(0.00) | 0(0.00) | 0(0.00) |
| Asthenia | 20(10.31) | 7(3.61) | 6(3.09) | 0(0.00) | 0(0.00) | 4(4.04) | 0(0.00) | 0(0.00) | 0(0.00) | 0(0.00) |
| Blood alkaline phosphatase increase | 25(12.89) | 3(1.55) | 4(2.06) | 0(0.00) | 0(0.00) | 7(7.07) | 1(1.01) | 0(0.00) | 0(0.00) | 0(0.00) |
| Occult blood positive | 31(15.98) | 0(0.00) | 0(0.00) | 0(0.00) | 0(0.00) | 13(13.13) | 0(0.00) | 0(0.00) | 0(0.00) | 0(0.00) |
| Abdominal pain | 15(7.73) | 11(5.67) | 1(0.52) | 0(0.00) | 0(0.00) | 0(0.00) | 0(0.00) | 0(0.00) | 0(0.00) | 0(0.00) |
| Hypocalcemia | 18(9.28) | 7(3.61) | 1(0.52) | 1(0.52) | 0(0.00) | 0(0.00) | 0(0.00) | 0(0.00) | 0(0.00) | 0(0.00) |
| Decreased appetite | 14(7.22) | 8(4.12) | 5(2.58) | 0(0.00) | 0(0.00) | 0(0.00) | 0(0.00) | 0(0.00) | 0(0.00) | 0(0.00) |
| Hypoalbuminemia | 17(8.76) | 8(4.12) | 0(0.00) | 0(0.00) | 0(0.00) | 5(5.05) | 1(1.01) | 0(0.00) | 0(0.00) | 0(0.00) |
| Pyrexia | 17(8.76) | 4(2.06) | 1(0.52) | 0(0.00) | 0(0.00) | 5(5.05) | 0(0.00) | 0(0.00) | 0(0.00) | 0(0.00) |
| Sinus tachycardia | 19(9.79) | 3(1.55) | 0(0.00) | 0(0.00) | 0(0.00) | 2(2.02) | 0(0.00) | 0(0.00) | 0(0.00) | 0(0.00) |
| Hypophosphatasemia | 20(10.31) | 0(0.00) | 1(0.52) | 0(0.00) | 0(0.00) | 1(1.01) | 0(0.00) | 0(0.00) | 0(0.00) | 0(0.00) |
| Upper abdominal pain | 11(5.67) | 9(4.64) | 0(0.00) | 0(0.00) | 0(0.00) | 0(0.00) | 0(0.00) | 0(0.00) | 0(0.00) | 0(0.00) |
| Hyperglycemia | 16(8.25) | 0(0.00) | 0(0.00) | 0(0.00) | 0(0.00) | 9(9.09) | 1(1.01) | 0(0.00) | 0(0.00) | 0(0.00) |
| Data are expressed as n (%). AEs in at least 10% of the patients for any grade in either group are reported regardless of the relationship to culmerciclib, fulvestrant, or placebo. | | | | | | | | | | |

**Supplementary Table 5:** The duration (days) of grade 2 or worse TEAEs in the safety set

| Preferred terms | Culmerciclib plus fulvestrant  N=194 | Placebo plus fulvestrant  N=99 |
| --- | --- | --- |
| Neutrophil count decrease |  |  |
| Grade 2 | 31.0(28.0,57.0) | 29.0(28.0,57.0) |
| Grade 3 | 6.0(2.0,15.0) | 16.5(4.0,29.5) |
| Grade 4 | 3.5(2.5,4.5) | 4.0(4.0,4.0) |
| Leucocyte count decrease |  |  |
| Grade 2 | 35.0(29.0,64.0) | 29.0(16.0,63.0) |
| Grade 3 | 4.0(2.0,11.0) | 4.0(2.0,12.0) |
| Diarrhea |  |  |
| Grade 2 | 24.5(5.0,52.5) |  |
| Grade 3 | 6.0(3.0,13.0) |  |
| Vomiting |  |  |
| Grade 2 | 12.0(4.0,32.0) | 14.0(2.0,22.0) |
| Grade 3 | 20.0(6.0,29.0) |  |
| Anemia |  |  |
| Grade 2 | 56.0(29.0,98.0) | 13.5(12.0,15.0) |
| Grade 3 | 18.0(10.0,32.0) |  |
| Lymphocyte count decrease |  |  |
| Grade 2 | 29.0(14.0,58.0) | 29.0(14.0,57.0) |
| Grade 3 | 9.0(4.0,14.0) |  |
| Hypertriglyceridemia |  |  |
| Grade 2 | 29.0(28.0,57.0) | 30.0(29.0,48.5) |
| Grade 3 | 30.0(14.0,46.0) | 29.0(29.0,29.0) |
| Grade 4 | 18.5(5.0,29.0) | 3.0(3.0,3.0) |
| Nausea |  |  |
| Grade 2 | 16.5(3.5,57.0) | 11.0(8.0,22.0) |
| Grade 3 | 20.5(7.0,34.0) |  |
| Urinary tract infection |  |  |
| Grade 2 | 54.5(29.0,86.0) | 54.0(29.0,57.0) |
| Grade 3 | 8.5(6.0,11.0) |  |
| γ-glutamyl transferase increase |  |  |
| Grade 2 | 30.0(28.0,74.0) | 9.0(9.0,9.0) |
| Grade 3 | 41.0(22.0,74.0) |  |
| Grade 4 | 9.0(9.0,9.0) |  |
| Body weight decrease |  |  |
| Grade 2 | 61.0(39.0,149.0) |  |
| Platelet count decrease |  |  |
| Grade 2 | 12.0(6.0,30.0) | 30.0(30.0,30.0) |
| Grade 3 | 7.0(6.0,19.0) |  |
| Grade 4 | 15.5(8.0,23.0) |  |
| Abdominal pain |  |  |
| Grade 2 | 5.5(3.0,10.0) |  |
| Grade 3 | 6.0(6.0,6.0) |  |
| Alanine aminotransferase increase |  |  |
| Grade 2 | 27.0(6.0,29.0) | 31.0(29.0,33.0) |
| Grade 3 | 7.0(5.0,9.0) |  |
| Aspartate aminotransferase increase |  |  |
| Grade 2 | 24.5(7.0,39.0) | 30.0(27.0,33.0) |
| Grade 3 | 8.0(6.0,14.0) |  |
| Fatigue |  |  |
| Grade 2 | 8.0(5.0,10.0) |  |
| Grade 3 | 32.0(26.0,39.0) |  |
| Decreased appetite |  |  |
| Grade 2 | 23.5(9.0,39.0) |  |
| Grade 3 | 10.0(6.0,12.0) |  |
| Upper abdominal pain |  |  |
| Grade 2 | 9.5(4.5,10.5) |  |
| Hypocalcemia |  |  |
| Grade 2 | 14.0(9.0,44.0) |  |
| Grade 3 | 4.0(3.0,5.0)) |  |
| Grade 4 | 16.0(16.0,16.0) |  |
| Hypoalbuminemia |  |  |
| Grade 2 | 11.5(4.0,18.0) |  |
| Blood alkaline phosphatase increase |  |  |
| Grade 2 | 33.0(11.0,35.0) | 28.0(28.0,28.0) |
| Grade 3 | 14.5(10.0,19.0) |  |
| Blood creatinine level increase |  |  |
| Grade 2 | 29.0(29.0,46.0) |  |
| Grade 3 | 26.5(13.0,40.0) |  |
| Hypokalemia |  |  |
| Grade 2 | 9.0(8.0,23.0) | 6.0(6.0,6.0) |
| Grade 3 | 9.0(5.5,29.5) | 6.0(6.0,6.0) |
| Grade 4 | 3.0(2.5,9.0) |  |
| Pyrexia |  |  |
| Grade 2 | 2.0(2.0,3.0) |  |
| Grade 3 | 2.0(2.0,2.0) |  |
| Hypercholesterolemia |  |  |
| Grade 2 | 31.5(18.5,45.5) | 58.0(58.0,58.0) |
| Grade 3 |  | 15.0(15.0,15.0) |
| Sinus tachycardia |  |  |
| Grade 2 | 28.0(28.0,28.0) |  |
| Hyperglycemia |  | 57.0(57.0,57.0) |
| Grade 2 |  |  |
| Hypophosphatemia |  |  |
| Grade 3 | 2.0(2.0,2.0) |  |
| Blood lactate dehydrogenase increase |  |  |
| Grade 3 | 4.0(4.0,4.0) |  |
| Hyperuricemia |  |  |
| Grade 3 | 2.0(2.0,2.0) |  |
| Data are expressed as median (Q1,Q3) | |  |

**Supplementary table 6:** Overall summary of serious adverse events in the safety population

| Adverse events | Culmerciclib plus fulvestrant  N=194 | Placebo plus fulvestrant  N=99 |
| --- | --- | --- |
| Any serious adverse events | 36 (18.6) | 10 (10.1) |
| Anemia | 6 (3.1) | 0 (0.0) |
| Leukopenia | 4 (2.1) | 0 (0.0) |
| Platelet count decreased | 4 (2.1) | 0 (0.0) |
| Hypokalemia | 4 (2.1) | 0 (0.0) |
| Pleural effusion | 3 (1.6) | 3 (3.0) |
| Ascites | 2 (1.0) | 0 (0.0) |
| Cerebral infarction | 2 (1.0) | 0 (0.0) |
| Infectious pneumonia | 1 (0.5) | 2 (2.0) |
| Disease progression | 1 (0.5) | 2 (2.0) |
| Serious adverse events occurring in more than one patient in either groups are listed regardless of the relationship to culmerciclib, fulvestrant, or placebo. | | |

**Supplementary table 7:** Overall summary of treatment-related adverse events (TRAEs) in the safety population.

|  | Culmerciclib plus fulvestrant  N=194 | | Placebo plus fulvestrant  N=99 | |
| --- | --- | --- | --- | --- |
|  | Any grade | Grade 3 or higher | Any grade | Grade 3 or higher |
| Any TRAEs | 191 (98.5) | 112 (57.7) | 87 (87.9) | 12 (12.1) |
| Neutropenia | 156 (80.4) | 48 (24.7) | 16 (16.2) | 4 (4.0) |
| Leukopenia | 155 (79.9) | 29 (15.0) | 20 (20.2) | 3 (3.0) |
| Alanine aminotransferase increased | 70 (36.1) | 4 (2.1) | 27 (27.3) | 0 (0.0) |
| Aspartate aminotransferase increased | 70 (36.1) | 7 (3.61) | 30 (30.3) | 0 (0.0) |
| Platelet count decreased | 69 (35.6) | 5 (2.6) | 6 (6.1) | 0 (0.0) |
| Lymphocyte count decreased | 49 (25.3) | 12 (6.2) | 8 (8.1) | 0 (0.0) |
| γ-glutamyl transferase increased | 45 (23.2) | 10 (5.2) | 6 (6.1) | 0 (0.0) |
| Body weight decreased | 41 (21.1) | 0 (0.0) | 1 (1.0) | 0 (0.0) |
| Blood creatinine level increased | 32 (16.5) | 2 (1.0) | 3 (3.0) | 0 (0.0) |
| Lactate dehydrogenase increased | 30 (15.5) | 0 (0.0) | 3 (3.0) | 0 (0.0) |
| Alkaline phosphatase increased | 25 (12.9) | 4 (2.1) | 8 (8.1) | 0 (0.0) |
| Occult blood positive | 22 (11.3) | 0 (0.0) | 11 (11.1) | 0 (0.0) |
| Diarrhea | 169 (87.1) | 14 (7.2) | 6 (6.1) | 0 (0.0) |
| Vomiting | 117 (60.3) | 4 (2.1) | 7 (7.1) | 0 (0.0) |
| Nausea | 82 (42.3) | 2 (1.0) | 9 (9.1) | 0 (0.0) |
| Abdominal pain | 26 (13.4) | 1 (0.5) | 0 (0.0) | 0 (0.0) |
| Hypertriglyceridemia | 67 (34.5) | 14 (7.2) | 24 (24.2) | 2 (2.0) |
| Hypokalemia | 50 (25.8) | 23 (11.9) | 3 (3.0) | 0 (0.0) |
| Hypercholesterolemia | 47 (24.2) | 0 (0.0) | 19 (19.2) | 1 (1.0) |
| Hyperuricemia | 33 (17.0) | 0 (0.0) | 6 (6.1) | 0 (0.0) |
| Decreased appetite | 27 (13.9) | 5 (2.6) | 0 (0.0) | 0 (0.0) |
| Hypocalcemia | 21 (10.8) | 2 (1.0) | 0 (0.0) | 0 (0.0) |
| Anemia | 121 (62.4) | 21 (10.8) | 11 (11.1) | 0 (0.0) |
| Asthenia | 28 (14.4) | 6 (3.1) | 4 (4.0) | 0 (0.0) |
| Urinary tract infection | 32 (16.5) | 1 (0.5) | 8 (8.1) | 0 (0.0) |
