# Supplementary material for: Novel CDK2/4/6 inhibitor culmerciclib (TQB3616) plus fulvestrant in previously treated, HR-positive, HER2-negative advanced breast cancer: a randomized, double-blind, phase 3 trial
Source: Signal Transduct Target Ther. 2025 Dec 18;10:414. doi: 10.1038/s41392-025-02475-6 (PMC12714862; doi:10.1038/s41392-025-02475-6)
Supplement: Supplementary file 2 — Protocol [file 41392_2025_2475_MOESM2_ESM.docx]

TQB3616 in Combination with Fulvestrant Versus Placebo in Combination with Fulvestrant in Previously Treated HR-Positive, HER2-Negative Advanced Breast Cancer: A Randomized, Double-Blind, Parallel-Controlled Phase III Trial

| Protocol No. | TQB3616-III-01 |
| --- | --- |
| Version No. | 4.0 |
| Version Date | June 10, 2022 |
| Leading site | The Fifth Medical Center of Chinese People’s Liberation Army General Hospital  Jiangsu Province Hospital |
| Principal Investigator | Professor Zefei Jiang  Professor Yongmei Yin |
| Statistical Analysis Unit | Department of Biostatistics, School of Public Health, Nanjing Medical University |
| Sponsor | Chia Tai Tianqing Pharmaceutical Group Co., Ltd. |

**Confidentiality Statement**

| This document contains confidential information and is only used by clinical researchers. The copyright is owned by Chia Tai Tianqing Pharmaceutical Group Co., Ltd. No information contained in this document may be released or disclosed to any third party (group or individual) without prior written permission. If you have this document without prior authorization, please contact Chia Tai Tianqing Pharmaceutical Group Co., Ltd., and return the document and its copy to the company. |
| --- |

Organization Information Page

| Sponsor | Chia Tai Tianqing Pharmaceutical Group Co., Ltd. |
| --- | --- |
|  | Responsibilities: Responsible for initiating and applying for work, providing investigator manuals, research drugs, and trial funding as well as being responsible for organizing, monitoring, auditing, and assisting in the design of clinical trial protocols. |
|  | Address: No. 1099, Fuying Road, Jiangning District, Nanjing 210046, Jiangsu Province, China. |
|  | Chief of Project: Yanfeng Bai; Tel.: +86-18635018442; E-mail: YANFENG.BAI@CTTQ.COM  Chief of Medicine: Fan Feng; Tel.: +86-13260882293; E-mail: fengfan2014cpu@163.com |
| Leading site | The Fifth Medical Center of Chinese People’s Liberation Army General Hospital  Jiangsu Province Hospital |
|  | Responsibilities: Recruiting subjects, designing clinical study protocols, organizing and implementing studies, and accepting inspections by regulatory authorities. |
|  | Address: No. 8 Dongdajie, Fengtai District, Beijing  No. 300 Guangzhou Road, Nanjing City, Jiangsu Province |
|  | Principal investigator: Prof. Zhefei Jiang; Tel: +86-010-66947797  Principal Investigator: Prof. Yongmei Yin; Tel.: +86-025-83718836 |
| Data Management Unit | Chia Tai Tianqing Pharmaceutical Group Co., Ltd. |
|  | Function: Responsible for data management of the clinical trial. |
|  | Address: No. 1099, Fuying Road, Jiangning District, Nanjing |
|  | Responsible person: Yadong Miao; Contact information: +86-18551674600 |
| Clinical research statistics center | Department of Biostatistics, School of Public Health, Nanjing Medical University |
|  | Responsibilities: Responsible for statistical analysis of clinical trial. |
|  | Address: No. 818, Tianyuan East Road, Jiangning District, Nanjing |
|  | Responsible: Hao Yu; Tel.: +86-13814056262 |

Protocol Signatures Pages (Sponsor)

We have read this clinical trial protocol (Protocol No.: TQB3616-III-01, Version No.4.0, Version date: June 10, 2022), and I agree to perform my duties as sponsor in accordance with the laws of the People's Republic of China, the Declaration of Helsinki, the GCP and this study protocol.

Sponsor of clinical trial: Chia Tai Tianqing Pharmaceutical Group Co., Ltd.

| Xunqiang Wang |  | |  |
| --- | --- | --- | --- |
| Clinical Technical Leader (Print) | | Signature | Date of Signature (DD/MMM/YYYY) |

Protocol Signatures Pages (Leading site)

I will earnestly perform my duties and personally participate in or directly guide this clinical trial in accordance with the current GCP regulations. I have read this clinical research protocol, protocol (Protocol No.: TQB3616-III-01, Version No.4.0, Version date: June 10, 2022). And I agree to perform my duties as an investigator in accordance with the laws of the People's Republic of China, the Declaration of Helsinki, the Good Clinical Practice and this clinical research protocol.

I agree to sign off and read the relevant safety information of the clinical trial provided by the sponsor in a timely manner and to consider the treatment of the subject, whether to adjust it accordingly, to communicate with the subject at the earliest possible time, if necessary, and to report any suspected and unintended serious adverse reactions provided by the sponsor to the Ethics Committee.

Leading site of clinical trial: The Fifth Medical Center of Chinese People’s Liberation Army General Hospital

| Zefei Jiang |  |  |
| --- | --- | --- |
| Principal Investigator (Print) | Signature of Principal Investigator | Date of Signature (DD/MMM/YYYY) |

Protocol Signatures Pages (Leading site)

I will earnestly perform my duties and personally participate in or directly guide this clinical trial in accordance with the current GCP regulations. I have read this clinical research protocol, protocol (Protocol No.: TQB3616-III-01, Version No.4.0, Version date: June 10, 2022). And I agree to perform my duties as an investigator in accordance with the laws of the People's Republic of China, the Declaration of Helsinki, the Good Clinical Practice and this clinical research protocol.

I agree to sign off and read the relevant safety information of the clinical trial provided by the sponsor in a timely manner and to consider the treatment of the subject, whether to adjust it accordingly, to communicate with the subject at the earliest possible time, if necessary, and to report any suspected and unintended serious adverse reactions provided by the sponsor to the Ethics Committee.

Leading site of clinical trial: Jiangsu Province Hospital

| Yongmei Yin |  |  |
| --- | --- | --- |
| Principal Investigator (Print) | Signature of Principal Investigator | Date of Signature (DD/MMM/YYYY) |

Protocol Signatures Pages (Study site)

I will earnestly perform my duties and personally participate in or directly guide this clinical trial in accordance with the current GCP regulations. I have read this clinical research protocol, protocol (Protocol No.: TQB3616-III-01, Version No.4.0, Version date: June 10, 2022). And I agree to perform my duties as an investigator in accordance with the laws of the People's Republic of China, the Declaration of Helsinki, the Good Clinical Practice and this clinical research protocol.

I agree to sign off and read the relevant safety information of the clinical trial provided by the sponsor in a timely manner and to consider the treatment of the subject, whether to adjust it accordingly, to communicate with the subject at the earliest possible time, if necessary, and to report any suspected and unintended serious adverse reactions provided by the sponsor to the Ethics Committee.

Study site:

|  |  |  |
| --- | --- | --- |
| Principal Investigator (Print) | Signature of Principal Investigator | Date of Signature (DD/MMM/YYYY) |

Protocol Signatures Pages (Statistical Analysis Unit)

I have read this clinical research protocol, protocol (Protocol No.: TQB3616-III-01, Version No.4.0, Version date: June 10, 2022). And I agree to perform my duties as an investigator in accordance with the laws of the People's Republic of China, the Declaration of Helsinki, the Good Clinical Practice and this clinical research protocol.

Clinical study institution: Department of Biostatistics, School of Public Health, Nanjing Medical University

| Hao Yu |  |  |
| --- | --- | --- |
| Statistical Analysis Unit Representative (Print) | Signature | Date of Signature (DD/MMM/YYYY) |

Protocol Update

| Version No. | Version Date | Updated |
| --- | --- | --- |
| 1.0 | July 22, 2021 | N/A |
| 2.0 | November 05, 2021 | 1. Revised the sample size and inclusion criteria, and improve the dose adjustment criteria of study drugs;  2. According ICH-E9 (R1), the estimation objectives are clearly defined, including concurrent events and concurrent event processing strategy. |
| 3.0 | February 10, 2022 | The sample size and previous treatment history requirements of the inclusion criteria were improved. |
| 4.0 | June 10, 2022 | 1. Delete the content of pharmacokinetics;  2. According ICH-E9 (R1), the estimation objectives are clearly defined, including concurrent event processing strategy;  3. Revising the description of blind design;  4. Improving image evaluation requirements;  5. Update the section of data management. |

CONTENTS

[Protocol Signatures Pages (Sponsor) 1](#_Toc183092853)

[Protocol Signatures Pages (Leading site) 2](#_Toc183092854)

[Protocol Signatures Pages (Leading site) 3](#_Toc183092855)

[Protocol Signatures Pages (Study site) 4](#_Toc183092856)

[Protocol Signatures Pages (Statistical Analysis Unit) 5](#_Toc183092857)

[Protocol Update 6](#_Toc183092858)

[Abbreviations 11](#_Toc183092859)

[Protocol Synopsis 12](#_Toc183092860)

[Subject Visit Schedule 19](#_Toc183092861)

[1. Introduction 23](#_Toc183092862)

[1.1 Study background 23](#_Toc183092863)

[1.2 Study Rationale 24](#_Toc183092864)

[1.2.1 Pharmacodynamic study results 24](#_Toc183092865)

[1.2.2 Results of pharmacokinetic studies 27](#_Toc183092866)

[1.2.3 Toxicological study results 29](#_Toc183092867)

[1.2.4 Clinical study of TQB3616 30](#_Toc183092868)

[1.3 Potential Risks and Benefits 31](#_Toc183092869)

[1.3.1 Known potential risks 31](#_Toc183092870)

[1.3.2 Known Possible Benefits 32](#_Toc183092871)

[2. Objectives and endpoints 32](#_Toc183092872)

[2.1 Primary objective 33](#_Toc183092873)

[2.2 Secondary objectives 33](#_Toc183092874)

[2.3 Primary endpoint 33](#_Toc183092875)

[2.4 Secondary endpoints 33](#_Toc183092876)

[3. Study Design 33](#_Toc183092877)

[3.1 Overall design 33](#_Toc183092878)

[3.2 Sample size estimation 33](#_Toc183092879)

[3.3 Interim Analyses 34](#_Toc183092880)

[3.4 Methods to reduce offset 34](#_Toc183092881)

[3.4.1 Randomization 34](#_Toc183092882)

[3.4.2 Blind design 35](#_Toc183092883)

[3.4.3 Handling of randomization errors 36](#_Toc183092884)

[4. Estimated objectives 36](#_Toc183092885)

[4.1 Primary estimated objective 36](#_Toc183092886)

[4.2 Secondary estimated objective 1 37](#_Toc183092887)

[4.3 Secondary estimated objective 2 37](#_Toc183092888)

[4.4 Secondary estimated objective 3 38](#_Toc183092889)

[4.5 Secondary estimated objective 4 38](#_Toc183092890)

[4.6 Secondary estimated objective 5 38](#_Toc183092891)

[5. Subject selection and withdrawal 38](#_Toc183092892)

[5.1 Inclusion criteria 38](#_Toc183092893)

[5.2 Exclusion criteria 40](#_Toc183092894)

[5.3 Withdrawal or termination criteria 42](#_Toc183092895)

[5.3.1 Withdrawal criteria 42](#_Toc183092896)

[5.3.2 Termination criteria 43](#_Toc183092897)

[5.3.3 Procedures for withdrawal or termination 43](#_Toc183092898)

[5.3.4 Lost to follow-up 44](#_Toc183092899)

[6. Investigational drugs 44](#_Toc183092900)

[6.1 Basic information 44](#_Toc183092901)

[6.2 Drug Management 44](#_Toc183092902)

[6.2.1 Dosing Regimen 44](#_Toc183092903)

[6.2.2 Dose Modification 44](#_Toc183092904)

[6.2.3 Drug Management 46](#_Toc183092905)

[6.2.4 Drug storage 46](#_Toc183092906)

[6.3 Concomitant medication and treatments 46](#_Toc183092907)

[6.3.1 Prohibited concomitant medications 47](#_Toc183092908)

[6.3.2 Concomitant medication allowing with caution 47](#_Toc183092909)

[6.3.3 Permitted concomitant medications 47](#_Toc183092910)

[7. Trail procedures 48](#_Toc183092911)

[7.1 Screening Visit 48](#_Toc183092912)

[7.2 Treatment period visits 50](#_Toc183092913)

[7.3 End of treatment (EOT) Visit 51](#_Toc183092914)

[7.4 Follow-up visits 51](#_Toc183092915)

[7.5 Unscheduled Visit 52](#_Toc183092916)

[8. Efficacy evaluation 52](#_Toc183092917)

[8.1 Evaluation Frequency 52](#_Toc183092918)

[8.2 Evaluation criteria 53](#_Toc183092919)

[8.3 Requirements for image evaluation 53](#_Toc183092920)

[8.4 Validation process of PD 53](#_Toc183092921)

[8.5 Evaluation indicators 54](#_Toc183092922)

[9. Safety evaluation 54](#_Toc183092923)

[9.1 Adverse events 54](#_Toc183092924)

[9.2 Evaluation of Adverse Events 54](#_Toc183092925)

[9.3 Records of adverse events 55](#_Toc183092926)

[9.4 Follow-up of adverse events 58](#_Toc183092927)

[9.5 Causality to Study Drugs 58](#_Toc183092928)

[9.6 Serious adverse event 58](#_Toc183092929)

[9.7 Management of Serious Adverse Events 59](#_Toc183092930)

[10. Source Documents and Obtaining Source Data/Documents 59](#_Toc183092931)

[11. Quality control and quality assurance 60](#_Toc183092932)

[11.1 Quality assurance of clinical trial process 60](#_Toc183092933)

[11.2 Quality assurance of the data transfer, calculation, and reporting process 60](#_Toc183092934)

[12. Data Management 61](#_Toc183092935)

[13. Statistical Analysis 63](#_Toc183092936)

[13.1 Analysis Datasets 63](#_Toc183092937)

[13.2 Statistical analysis plan 63](#_Toc183092938)

[13.2.1 General principles 63](#_Toc183092939)

[13.2.2 Test level 63](#_Toc183092940)

[13.2.3 Hypothesis test 63](#_Toc183092941)

[13.2.4 Stratification factors/covariates 64](#_Toc183092942)

[13.3 Study Population 64](#_Toc183092943)

[13.3.1 Sugjects distribution 64](#_Toc183092944)

[13.3.2 Protocol deviations 64](#_Toc183092945)

[13.3.3 Baseline characteristics Analysis of the two groups 64](#_Toc183092946)

[13.4 Efficacy evaluation 64](#_Toc183092947)

[13.4.1 Primary estimated objective 65](#_Toc183092948)

[13.4.2 Secondary estimated objective 1 65](#_Toc183092949)

[13.4.3 Secondary estimated objective 2 65](#_Toc183092950)

[1 3.4.4 Secondary estimated objective 3 66](#_Toc183092951)

[1 3.4.5 Secondary estimated objective 4 66](#_Toc183092952)

[1 3.4.6 Secondary Estimate Objective 5 66](#_Toc183092953)

[13.5 Safety evaluation 66](#_Toc183092954)

[13.5.1 Drug exposure 66](#_Toc183092955)

[13.5.2 Adverse events 66](#_Toc183092956)

[13.5.3 Vital Signs 67](#_Toc183092957)

[13.5.4 Laboratory test indicators 67](#_Toc183092958)

[13.5.5 Electrocardiogram 67](#_Toc183092959)

[13.5.6 Concomitant medication 68](#_Toc183092960)

[13.6 Missing Data 68](#_Toc183092961)

[13.7 Subgroup analysis 68](#_Toc183092962)

[13.8 Statistical analysis software 69](#_Toc183092963)

[14. Ethics and Informed Consent 69](#_Toc183092964)

[15. Publication of trial results 69](#_Toc183092965)

[16. References 69](#_Toc183092966)

Abbreviations

| AE | Adverse events | HR | Hormone receptor |
| --- | --- | --- | --- |
| AKP | Alkaline phosphatase | IC_50_ | Half-maximal inhibitory concentration |
| ALT | Glutamate Alanine Aminotransferase | IHC | Immunohistochemistry |
| ANC | Neutrophil count | IU | International Units |
| AST | Glutamate Aspartate Aminotransferase | K + | Blood potassium |
| BUN | Urea nitrogen | MTD | Maximum tolerated dose |
| CBR | Clinical benefit rate | Na + | Blood sodium |
| CDK | Cyclin-dependent kinase | NYHA | New York Heart Association |
| CNS | Central nervous system | ORR | Objective response rate |
| Cr | Creatinine | OS | Overall survival |
| CR | Complete response | PD | Disease progression |
| CRF | Case Report Form | PFS | Progression-free survival |
| DLT | Dose limiting toxicity | PO | Oral |
| DoR | Duration of Response | PPS | Per Protocol Set |
| EC | Ethics Committee | PR | Partial response |
| ER | Estrogen receptor | PLT | Platelets |
| E2 | Estradiol | RBC | Red blood cell count |
| FAS | Full Analysis Set | RECIST | Response Evaluation Criteria in Solid Tumors |
| FISH | Fluorescence in situ hybridization | RNA | Ribonucleic acid |
| FSH | Follicular estrogen | RP2D | Recommended Phase 2 Dose |
| GCP | Good Clinical Practice | SAE | Serious adverse event |
| HB | Hemoglobin | SAP | Statistical Analysis Plan |
| HBV | Hepatitis B Virus | SD | Disease progression |
| HCV | Hepatitis C Virus | SS | Safety Set |
| HER-2 | Human epidermal growth factor receptor-2 | T-BIL | Total bilirubin |
| HIV | Human Immunodeficiency Virus | WBC | White blood cell count |

Protocol Synopsis

| Title | TQB3616 in Combination with Fulvestrant Versus Placebo in Combination with Fulvestrant in Previously Treated HR-Positive, HER2-Negative Advanced Breast Cancer: A Randomized, Double-Blind, Parallel-Controlled Phase III Trial |
| --- | --- |
| Protocol No. | TQB3616-III-01 |
| Version No./Date | 4.0 /June 10, 2022 |
| Sponsor | Chia Tai Tianqing Pharmaceutical Group Co., Ltd. |
| Leading site/principal investigator | The Fifth Medical Center of Chinese People’s Liberation Army General Hospital/ Professor Zefei Jiang  Jiangsu Province Hospital/ Professor Yongmei Yin |
| Objectives | Primary objective:   - To evaluate the progression free survival (PFS) of TQB3616 plus fulvestrant versus placebo plus fulvestrant in subjects with previously treated HR-positive, HER2-negative advanced breast cancer.   Secondary objectives:   - To evaluate the overall survival (OS), objective response rate (ORR), clinical benefit rate (CBR), duration of response (DOR) of TQB3616 plus fulvestrant versus placebo plus fulvestrant in subjects with previously treated HR-positive, HER2-negative advanced breast cancer. - To evaluate the safety of TQB3616 in combination with fulvestrant versus placebo in combination with fulvestrant in subjects with previously treated HR-positive, HER2-negative advanced breast cancer, including incidence and severity of adverse events (AEs), serious adverse events (SAEs) and abnormal laboratory values. |
| Endpoints | Primary endpoint   - PFS assessed by the investigator.   Secondary endpoints   - Efficacy indicators - PFS assessed by independent review committee; - OS; - ORR; - CBR; - DOR. - Safety indicators - Incidence and severity of AEs, SAEs and laboratory abnormalities. |
| Subjects | HR positive, HER2-negative locally advanced or metastatic breast cancer patients |
| Sample Size estimation | This study uses a randomized, double-blind, parallel-controlled, multicenter trial designand PFS is the primary endpoint.  The number of patients required for this trial was based on the primary end point of investigator-assessed PFS and was calculated with the use of a predefined stratified log-rank test. Assuming a median PFS of 6.0 months for placebo plus fulvestrant, we estimated that 186 events of progression or death would be required in the two treatment groups for the study to have 90% power to detect a hazard ratio (HR) of 0.60 with a two-sided significance level of α = 0.05. A total sample of 243 patients was required. Assuming an attrition rate of 15%, at least 287 patients (191 for the experimental group and 96 for the control group) were anticipated. The primary end point was to be analyzed at the interim analysis at approximately 70% maturity in the overall population when 131 events of progression or death had occurred and at the final analysis at 100% maturity when 186 events of progression or death had occurred. Type I errors were controlled using the Lan-DeMets spending function approximating O’Brien-Fleming boundary. |
| Study Design | This is a randomized, double-blind, parallel-controlled, multicenter study. Patients were randomized in a 2:1 ratio to receive TQB3616 plus fulvestrant (experimental group) or placebo plus fulvestrant (control group). Randomization is stratified according to:   1. visceral metastatic disease (yes vs. no) 2. menopausal status (pre-, peri- or postmenopausal) 3. sensitivity to prior endocrine therapy (yes vs. no).   Efficacy assessments are performed every 8 weeks (56 days) and for every 12 weeks (84 days) after 48 weeks. For patients with disease control (CR + PR + SD) and tolerable adverse reactions, the treatment can be continued until loss of clinical benefit, intolerable toxicity and the investigator consider it inappropriate to continue the treatment. |
| Interim Analyses | An Independent Data Monitoring Committee (IDMC) will be established for this study to conduct an interim analysis. The primary end point (PFS) is to be analyzed at the interim analysis at approximately 70% maturity in the overall population when 131 PFS events had occurred. Type I errors were controlled using the Lan-DeMets spending function approximating O’Brien-Fleming boundary with a two-sided significance level of α = 0.05:   \| Number of events \| Test level \| \| --- \| --- \| \| 131 (70 %) \| 0.01477 \| \| 186 \| 0.04551 \|   Note: The actual nominal test level at interim analysis will be determined according to the proportion of events at that time. The nominal test level for the final analysis will be adjusted accordingly.  In actual operation, if the proportion of events exceeds 70% in interim analysis, the corresponding nominal test level will be recalculated according to the O 'Brien-Fleming α-consumption function method, as described in the IDMC charter. Based on the analysis results, the IDMC made a written recommendation to the sponsor on whether the trial is conducted or not and whether the data can be summarized in advance. The sponsor will take appropriate decisions on this study project according to the IDMC recommendations, such as terminating the trial due to safety or lack of efficacy or summarizing the application in advance due to drug efficacy, and supplementing the data after the end of the entire trial. |
| Inclusion criteria | Subjects who meet all of the following inclusion criteria can be included in this trial:   1. Voluntarily participate in this study, sign the ICF and have good compliance; 2. Age 18-75 years (calculated on the date of signing the informed consent form); ECOG PS of 0-1; predicted life expectancy of ≥3 months; 3. Postmenopausal or premenopausal/perimenopausal female patients who meet any of the following:  - Prior Oophorectomy; - Age ≥ 60 years; - Age < 60 years, natural menopause ≥ 12 months (no chemotherapy, tamoxifen, toremifene, or ovarian castration drugs have been administered in the past year), with follicle stimulating hormone (FSH), and estradiol (E2) levels within postmenopausal range; - Premenopausal or perimenopausal patients may also be eligible, if willing to accept LHRH agonist therapy during the study.  1. Histopathologically confirmed HR-positive, HER2-negative, locally recurrent or metastatic breast cancer, not amenable to curative resection or radiotherapy, or clinically not indicated for chemotherapy:  - HR positive status was defined as positive (≥ 10%) estrogen receptor expression or progesterone receptor expression (confirmed by the pathology department of the research center); - HER2-negative status was defined as 0 or 1+ intensity on immunohistochemical testing, 2+ intensity on immunohistochemical testing and *in-situ* hybridization-negative, or *in-situ* hybridization-negative in the absence of immunohistochemical testing (confirmed by the pathology department of the research center);  1. Eligible participants with prior endocrine therapy must meetone of the following three criteria: 2. relapse or progression during or within 1 year after completion of adjuvant endocrine therapy without subsequent endocrine therapy; 3. recurrence or progression more than 1 year after completion of adjuvant endocrine therapy, and re-progression after receiving rescue endocrine therapy; 4. progression after rescue endocrine therapy after an initial diagnosis of locally advanced or metastatic disease;   Note: ① Recurrence or progression of previous treatment should be confirmed by imaging examination;  ② The time required to receive adjuvant endocrine therapy is not less than 1 year.   1. must have received no more than one line of rescue endocrine therapy or rescue chemotherapy for recurrent or metastatic disease; 2. required to have a measurable lesion according to the Response Evaluation Criteria in Solid Tumors (RECIST), version 1.1, or bone only metastasis including lytic or mixed lytic-blastic bone lesions; 3. Adequate function of the important organs as evidenced by the following:  - Blood routine examination (no blood transfusion within 7 days, no correction with hematopoietic stimulator drugs):   a) Hemoglobin (HB) ≥ 100 g/L;  b) Absolute neutrophil count (NEUT) ≥ 1.5 × 10 ^9^ /L;  c) Platelets Counts (PLT) ≥ 90 × 10 ^9^ /L.   - Blood Chemistry:   a) Total bilirubin (TBIL) ≤ 1.5×upper limit of normal value (ULN);  b) alanine aminotransferase (ALT) and aspartate aminotransferase (AST) ≤ 2.5 × ULN. If with liver metastases, then ALT and AST ≤ 5 × ULN;  c) Serum creatinine (CR) ≤ 1.5 × ULN , or creatinine clearance (CCR) ≥ 60 ml/min.   - Coagulation function:   Prothrombin time (PT), activated partial thromboplastin time (APTT), international normalized ratio (INR) ≤ 1.5 × ULN (no anticoagulant therapy);   - Echocardiography evaluation: left ventricular ejection fraction (LVEF) is ≥ 50%.  1. Women of childbearing potential and men must agree to take contraception for the duration of study treatment and 6 months after the last dose of study treatment (such as an intrauterine device, contraceptive pills, or condoms), and have a negative serum pregnancy test within 7 days prior to the study grouping and be on-lactating. |
| Exclusion Criteria | Subjects who meet any of the following exclusion criteria will not be enrolled in the study:   1. Previous pathological diagnosed HER2-positive breast cancer; 2. Bilateral breast cancer or inflammatory breast cancer; 3. Concomitant diseases and medical history: 4. Patients with other malignancies within 3 years excluding the following two conditions: other malignancies treated by single operation, reaching disease-free survival (DFS) for 5 consecutive years; cured cervical carcinoma in situ, non-melanoma skin cancer, and superficial bladder tumors [Ta (non-invasive tumor), Tis (carcinoma in situ), and T1 (tumor infiltrating basal lamina)]; 5. With multiple factors affecting oral and drug absorption (e.g., inability to swallow, post-GI resection, ulcerative colitis, symptomatic/inflammatory bowel disease, chronic diarrhea and intestinal obstruction and other gastrointestinal diseases); 6. History of severe pneumonia such as interstitial lung disease; 7. Unresolved toxicity higher than CTC AE Grade 1, excluding alopecia, due to any prior therapy; 8. Major surgical treatment, or significant traumatic injury within 28 days prior to the study. 9. Long-term unhealed wounds or fractures; 10. Arteriovenous thrombosis occurred within 6 months, such as cerebrovascular accident (including temporary ischemic attack, cerebral hemorrhage, cerebral infarction), deep vein thrombosis and pulmonary embolism; 11. Patients with a history of psychotropic drug abuse and unable to quit or with mental disorders; 12. Presence of any severe and/or uncontrolled disease: 13. Class II or more of myocardial ischemia or myocardial infarction, congestive heart failure according New York Heart Association (NYHA), treatable arrhythmia (including QTc ≥ 480 ms during screening period), or uncontrolled hypertension occurred within 6 months before the first dose; 14. Active or uncontrolled severe infection (CTC AE grade ≥2) or unexplained fever > 38.5°C in the 28 days prior to randomization; 15. Cirrhosis, active hepatitis*;   * Active hepatitis (hepatitis B reference: HBsAg positive, and HBV DNA detection value exceeds the upper limit of normal; hepatitis C reference: HCV antibody positive, and HCV virus detection value exceeds the upper limit of normal); note: eligible subjects with hepatitis B surface antigen positive or core antibody positive and hepatitis C patients require continuous antiviral therapy to prevent viral activation.   1. Patients with renal failure requiring hemodialysis or peritoneal dialysis; 2. History of immunodeficiency, including HIV positivity or other acquired, congenital immunodeficiency disease, or history of organ transplantation or hematopoietic stem cells transplant historian; 3. Tumor related symptoms and treatment: 4. Conditions with visceral crisis; 5. With central nervous system metastases (CNS), clinical evidence or history of carcinomatous meningitis, leptomeningeal disease; 6. Severe bone injury caused by tumor bone metastasis, including important pathological fracture and spinal cord compression within 6 months or expected to occur in the near future; 7. Received chemotherapy within 3 weeks before randomization, and radiotherapy (except palliative radiotherapy for nontarget lesions), endocrine therapy, or other antineoplastic therapy had been received within 2 weeks before randomization (the washout period is calculated from the end of the last treatment).; 8. Previous fulvestrant, everolimus, or CDK4/6 inhibitor therapy; 9. Uncontrolled pleural effusion, ascites, and pericardial effusion of equal or greater volume need to be drained repeatedly; 10. Known hypersensitivity for fulvestrant, LHRH agonists (i.e. Goserelin) TQB3616/ placebo or any of the excipients; 11. Attenuated live vaccination 28 days prior to the study or planned attenuated live vaccination during the study; 12. Participation in other clinical trials within 4 weeks prior to the study; 13. Subjects who have other serious physical or mental illness or laboratory abnormalities that may increase the risk of study participation, or interfere with study results, and who, in the opinion of the investigator, are otherwise unsuitable for this study. |
| Drugs | TQB3616 Capsules/Placebo Capsules, Fulvestrant Injection (Qingkeyi)  The above two drugs are produced and provided by Chia Tai Tianqing Pharmaceutical Group Co., Ltd. |
| Dosing regimen | TQB3616 /Placebo:  180 mg /0 mg, oral, QD for a 28-day cycle. It is recommended to take the drug at a fixed time every day; if there is missed dose during medication and the time interval between this dose and the next dose is less than 10 hours, the patient would not be redosed.  Fulvestrant:  500 mg, administered as an intramuscular injection, on day 1 and 15 of cycle 1 and day 1 of subsequent cycles. |
| Efficacy Evaluation | Evaluated using RECIST 1.1 criteria. |
| Safety Evaluation | NCINCI-CTC AE 5.0 criteria is used to determine the severity of adverse events. During the trial, the adverse event record form should be faithfully filled out, including the time of occurrence, severity, correlation with study drugs, duration, measures taken, and outcome of adverse events. |
| Safety indicators | Vital signs, physical examination, body weight, laboratory tests (hematology, blood biochemistry, urine routine, amylase, thyroid function, coagulation function, myocardial enzyme spectrum, etc.), 12-Lead ECG and echocardiography. |
| Duration of trial | March 2022 to December 2023 |

Subject Visit Schedule

| Study phase  (EPOCH) | Screening Period ^1^ | Treatment period ^2^ | | | | | | | Follow-up Period | | |
| --- | --- | --- | --- | --- | --- | --- | --- | --- | --- | --- | --- |
|  |  | Cycle 1 | | | Cycles 2-12 | | After Cycle 12 | EOT ^3^ | Safety Follow-up ^4^ | Efficacy Follow-up ^5^ | Survival Follow-up ^6^ |
|  |  |  |  |  | C2 N | C2n + 1 | C2n (n ≥ 7) |  |  |  |  |
| VISIT | Visit 1 | Visit 2 | Visit 3 | Visit 4 | Visit 5 | Visit 6 | Visit 7 | Within 7 days of EOT visit/study withdrawal | Within 28 days of last medication or initiation of new antineoplastic therapy | Time points for imaging assessment | Every 12 weeks |
| Evaluation time (day) | D-28 ~ -1 | D1 | D15 | D28 | D28 | D28 | D28 |  |  |  |  |
| Evaluation Window (days) | / | / | ± 1 day | ± 3 Day | ± 3 days | ± 3 days | ± 3 days | ± 7 days | ± 7 days | ± 7 days | ± 7 Day |
| Informed Consent | X |  |  |  |  |  |  |  |  |  |  |
| Demographics | X |  |  |  |  |  |  |  |  |  |  |
| Tumor history/past medical history | X |  |  |  |  |  |  |  |  |  |  |
| Trial Drug Treatment ^7^ |  | X (28-day cycle) | | | | | | |  |  |  |
| Height | X (within 7 days) |  |  |  |  |  |  |  |  |  |  |
| Physical examination, weight | X (within 7 days) |  | X | X | X | X | X | X |  |  |  |
| Vital Signs | X (within 7 days) |  | X | X | X | X | X | X |  |  |  |
| ECOG PS score | X (within 7 days) |  |  |  | X |  | X | X |  |  |  |
| Virological examination | X (within 14 days) |  |  |  |  |  |  |  |  |  |  |
| Pregnancy test ^8^ | X (within 7 days) |  |  |  |  |  |  |  |  |  |  |
| Hormone test ^9^ | X (within 7 days) |  |  |  | X |  | X | X |  |  |  |
| Blood routine | X (within 7 days) |  | X | X | X | X | X | X |  |  |  |
| Blood biochemistry | X (within 7 days) |  | X | X | X | X | X | X |  |  |  |
| Urine routine | X (within 7 days) |  | X | X | X | X | X | X |  |  |  |
| Stool routine | X (within 7 days) |  |  | X | X | X | X | X |  |  |  |
| 12-Electrocardiogram | X (within 7 days) |  | X | X | X | X | X | X |  |  |  |
| Echocardiogram | X (within 14 days) |  |  |  |  |  |  |  |  |  |  |
| Coagulation function | X (within 7 days) |  |  |  | X |  | X | X |  |  |  |
| Amylase, lipase | X (within 7 days) |  |  |  | X |  | X | X |  |  |  |
| Thyroid function | X (within 7 days) |  |  |  | X |  | X | X |  |  |  |
| Neoplasm Radiographic assessment ^1 0^ | X (within 28 days) |  |  |  | X |  | X | X |  | X |  |
| Tumor markers | X (within 7 days) |  |  |  | X |  | X | X |  | X |  |
| Bone Scan (EOT) ^1 0^ | X (within 42 days) |  |  |  | X |  | X | X |  | X |  |
| Safety Assessments | X | X | X | X | X | X | X | X | X |  |  |
| Concomitant medication/treatment | X | X | X | X | X | X | X | X | X |  |  |

Notes:

1. The screening period was 28 days, and the examination results that met the protocol requirements within the validity period before the subjects signed the informed consent form were acceptable. Physical examination, height, weight, ECOG score, vital signs, laboratory tests, tumor markers, 12-lead electrocardiogram results within 7 days before the first dose, echocardiography and virology results within 14 days before the first dose, bone scan results within 42 days before the first dose. Tumor imaging (CT or MRI of the head, neck, chest, abdomen, or pelvis) was received within 28 days before the first dose.

2. During treatment, visit on days 1, 15, and 28 of cycle 1; Weekly end-of-term (28-day) visits were conducted in cycles 2-12. After cycle 12, visits at the end of every two cycles (56 days), namely C14D28, C16D28, C18D28, C20D28... And other node visits.

3. End of Treatment (EOT) Visit: Examination steps for an end of treatment (EOT) visit are required when a subject meets any of the reasons for the end of study treatment. These tests were not required at the EOT visit if some tests were completed within 7 days before withdrawal.

4. Safety follow-up: A safety follow-up period was entered after the end-of-treatment visit to track safety events. Subjects should visit 28 days (±7 days) after the last treatment. If new antineoplastic therapy was initiated within 28 days after the last dose, the current visit was completed before new antineoplastic therapy.

5. Efficacy follow-up: subjects who discontinued the treatment due to non-disease progression or non-death reasons should continue to receive efficacy follow-up after the end of the trial drug, and tumor imaging evaluation was performed according to the time point specified in the protocol until the subject's disease progression, initiation of other anti-tumor drugs or death (whichever came first). The time of each follow-up, the results of tumor imaging evaluation and other anti-tumor treatment information were recorded in detail.

6. Survival follow-up (OS data collection): all non-death subjects who completed the safety follow-up and efficacy follow-up (whichever was completed later) were required to receive survival follow-up. Data on survival (date and cause of death) and post-study treatment (including treatment received) were collected at least once every 12 weeks (±7 days) by outpatient visits or telephone interviews with participants, their family members, or local physicians from the date of completion of safety and efficacy follow-up visits (whichever was completed later). Until death, loss of subjects to follow-up, or the end of OS data collection, whichever came first.

7. For premenopausal or perimenopausal patients, concomitant use of LHRH agonists is required: goserelin is recommended, and a 1-month dosage form is recommended. Subjects who had started medical castration before signing the informed consent form were preferred to switch to goserelin for the duration of the study.

8. Pregnancy test: blood pregnancy test, which is required only for patients with ovarian function suppression using LHRH agonists.

9. Hormone examination: follicle stimulating hormone (FSH), estradiol (E2). Except for those with bilateral oophorectomy or those aged > 60 years, baseline FSH and E2 were checked within 7 days before the first dose. Subjects using LHRH agonists may have FSH and E2 checked every 4 cycles as appropriate.

10. Tumor imaging evaluation: at least the head, neck, chest, abdomen and pelvis were included in the screening period, and at least the neck, chest, abdomen and pelvis were included in the subsequent cycle, and the other parts were performed according to clinical needs; For the first 12 cycles (48 weeks) of the treatment phase, check-ups were performed every 8 weeks; After cycle 12 (48 weeks), examinations were performed every 12 weeks. Bone scans generally do not need to be performed routinely during treatment and are only performed when clinically indicated, but repeat scans every 24 weeks are recommended for patients with baseline bone lesions. The time window allowed for tumor imaging was ±7 days. For subjects who discontinued the study drug for reasons other than disease progression and death, the follow-up imaging evaluation was continued after the end of the study drug. Tumor imaging evaluation was performed according to the time point specified in the protocol until the subject's disease progression, the initiation of other antineoplastic drugs or death (whichever came first). The time of each follow-up, the results of tumor imaging evaluation and other anti-tumor treatment information were recorded in detail.

1. Introduction
   1. Study background

According to the data of the World Cancer Report, in 2012, there were about 1.7 million new cases of breast cancer and about 500 000 deaths worldwide, accounting for 25% of all new cancers in women and 15% of all cancer deaths, ranking first in the world. In China, statistics show that in 2014, the number of new cases of breast cancer in Chinese women reached 268,000, accounting for 15% of all new cancers in women, ranking first, and about 70,000 deaths, accounting for 7% of all cancer deaths. From 2000 to 2011, the new cases and deaths showed an upward trend. Of new cases of breast cancer each year, 3%-10% of women have distant metastases at the time of diagnosis. Among patients with early-stage breast cancer, 30%-40% will develop into advanced breast cancer (ABC), and the 5-year survival rate is about 20%[1]

At present, there is no standard treatment for ABC patients. The overall median survival of ABC is 2-3 years and varies by molecular subtype. For human epidermal growth factor receptor 2 (HER2) positive ABC patients, anti-HER2 therapy can significantly prolong the survival time. However, the overall prognosis of triple-negative ABC patients has not been significantly improved. Hormone receptor-positive (HR+) breast cancer accounts for about 65-75% of breast cancer. Endocrine therapy has become the first choice for hormone receptor-positive (HR+) metastatic breast cancer due to its comparable therapeutic efficacy and less toxic side effects with chemotherapy.

Although endocrine therapy is the main treatment regimen for hormone receptor-positive breast cancer, there are about 30% of hormone receptor-positive breast cancers are primary resistant to endocrine therapy, and almost all patients develop secondary resistance during subsequent treatment, so how to overcome endocrine therapy resistance has become an urgent problem to be solved in the field of breast cancer treatment.

Studies have shown that overexpression of positive regulatory proteins of cell cycle or loss or low expression of negative regulatory proteins are involved in endocrine therapy resistance. The typical cell cycle is divided into: G0 (stationary phase), G1 (prophase of DNA synthesis), S (phase of DNA synthesis), G2 (prophase of division), and M (phase of cell division). G1 to S phase transition is the most critical checkpoint in the cell cycle, which is positively regulated by Cyclin and CDKs kinases, of which Cyclin D1/CDK4/6 is the most important. Cyclin D1 binds to CDK4/6 to form an activation complex that phosphorylates Rb protein, thereby releasing Rb protein's transcriptional repression of transcription factor E2F, causing the transcription of S-phase specific target genes, and causing the cell cycle to enter S phase from G1 phase [2]. Some studies also suggest that CyclinD-CDK4 can also phosphorylate SMAD2, FOXM1 and other targets, and directly act on pathways related to proliferation, metastasis and DNA damage response [3]. In addition, a large number of evidences have shown that overexpression of CyclinD1-CDK4/6 also plays an important role in the occurrence and development of breast cancer [4]. About 15%-25% of human breast cancers have CDK4 and CyclinD1 amplification, and about 50% of breast cancers have high expression of CyclinD1, and the type of breast cancer with high expression of CyclinD1 is mainly ER-positive breast cancer. All this information suggests that CDK4/6 inhibitors and endocrine therapy should have a synergistic effect against breast cancer and inhibit or delay the development of endocrine therapy resistance.

Palbociclib, developed by Pfizer, was approved by the FDA in 2015 for the first-line treatment of estrogen receptor-positive (ER +), human epidermal growth factor receptor 2-negative (HER2-) advanced or metastatic breast cancer in postmenopausal women. Later, it combined with fulvestrant was approved by the FDA in 2016 as second-line therapy for HR + /HER2- advanced or for metastatic breast cancer after progression after endocrine therapy. Ribociclib (Kisqali), developed by Novartis, was approved in 2017 in combination with aromatase inhibitors as first-line treatment for HR-positive/HER2-negative advanced or metastatic breast cancer in postmenopausal women. Abemaciclib was also approved in 2017 with fulvestrant in patients with HR-positive, HER2-negative advanced or metastatic breast cancer after endocrine therapy progression. At present, only Palbociclib and Abemaciclib have been approved for marketing in China, and Ribociclib is still in clinical development, but it is still far from meeting the clinical demand of hormone receptor-positive advanced or metastatic breast cancer in China.

TQB3616 capsule is a novel CDK2/4/6 kinase inhibitor developed by Chia Tai Tianqing Pharmaceutical Group Co., LTD. Preclinical data have shown that TQB3616 selectively inhibits CDK2/4/6 kinase activity to form a complex with Cyclin D, reduce the phosphorylation level of retinoblastoma protein (Rb) in cancer cells, and prevent cells from entering S phase, thereby inhibiting cell proliferation and anti-tumor effects. It is expected to be used in the treatment of advanced malignant solid tumors such as recurrent/metastatic breast cancer.

- 1. Study Rationale

1.2.1 Pharmacodynamic study results

Palbociclib and Abemaciclib were used as reference compounds for pharmacodynamic studies.

In vitro studies showed that TQB3616 strongly inhibited the activities of CDK4/cyclin D1 and CDK6/cyclin D1 with IC_50_ values of 0.35 and 0.49nM, respectively, and also inhibited CDK2/cyclin A with IC_50_ values of 2.62nM. TQB3616 inhibited the proliferation of 19 RB-positive and 1 RB-negative tumor cell lines. The IC_50_ of TQB3616 on ER+, Her2-, Rb+ breast cancer cell line MCF-7 was 89.34 nM. However, the cell line MDA-MB-436 with low expression of ER, Her2 and Rb had a significantly weaker inhibitory activity, and the IC_50_ was 548.38 nM. For the other 18 Rb+ tumor cell lines, TQB3616 at 300 nM inhibited the proliferation of 8 of them (BxPC3, DU145, NCI-H460, NCI-H520, SW620, T47D, KG-1, Mino) by more than 50%. The results of non-target kinase screening showed that TQB3616 inhibited the kinases CAMK2 alpha, CDK2, DYRK3, MAP4K4 and MINK with an IC_50_ of 10 Nm-100 nM. The IC_50_ for GSK3 beta, JNK1, TAOK2 and TNIK ranged from 101 nM to 1000 nM. The IC_50_ of other kinases was greater than 1000 nM. GPCR screening results showed that TQB3616 did not significantly inhibit or agonize the selected targets.

The inhibition of hERG potassium channels by compound TQB3616 by manual patch-clamp method showed that within the concentration range tested, the IC_50_ of TQB3616 on hERG potassium channel currents stably expressed in Chinese hamster ovary (CHO) cells was 2.76µM, and the slope was 1.03.

ER+, Her2-, Rb+ breast cancer cell line MCF-7 xenograft model in nude mice, CDK4/6 and CDK4/6 D type cell cycle binding protein (CCND1) high expression, Rb1 positive, negative regulatory gene p16INK4A deletion of human non-small cell lung cancer LU-01-0393 xenograft model. The efficacy of TQB3616 was studied in vivo.

In the human breast cancer line MCF-7 nude mice xenograft model, TQB3616 12.5 mg/kg showed a good anti-tumor effect at 20 days of administration, with tumor growth inhibition (TGI) and T/C of 112% and 20%, respectively. However, at a slightly higher dose of 18.75 mg/kg, TGI and T/C were 114% and 18%, respectively, and no more significant anti-tumor effect was observed compared with the dose of 12.5mg/kg. It may be related to the fact that the antitumor effect is very strong at the lowest dose. The dose of 25mg/kg was administered once a day for the first week and then once every two days for the next two weeks. The frequency of administration was lower than that of the two lower doses, and the anti-tumor effect was equivalent to that of the two lower doses, with TGI and T/C of 117% and 17%, respectively. Palbociclib at doses of 25 mg/kg and 50 mg/kg showed significant tumor suppression with T/C ratios of 42% and 26% and TGI of 82% and 104%, respectively. Abemaciclib at the dose of 25 mg/kg also showed a significant anti-tumor effect with T/C 19% and TGI 113%. The efficacy of TQB3616 at 12.5mg/kg was comparable to that of the reference compounds Palbociclib at 50mg/kg and Abemaciclib at 25mg/kg.

In order to explore the lowest effective dose and the repeatability of the experiment, the drug efficacy was tested again in the human breast cancer line MCF-7 nude mice xenograft model. At 20 days of dosing, the T/C of 5 mg/kg, 7.5 mg/kg, and 15 mg/kg TQB3616 was 45%, 52%, and 27%, respectively, and the TGI was 68%, 60%, and 93%, respectively. Palbociclib showed 59% and 37% T/C and 52% and 82% TGI at doses of 20 and 40 mg/kg, respectively. Abemaciclib had 66% and 43% T/C and 45% and 76% TGI in the 7.5 mg/kg and 15 mg/kg dose groups. In conclusion, TQB3616 at a dose of 15mg/kg could significantly inhibit the tumor growth of human cell line MCF7 breast cancer mouse xenograft model, and the efficacy was similar to Palbociclib (40 mg/kg) and Abemaciclib (15 mg/kg). The PD study showed that TQB3616 reduced p-RB levels after 15 mg/kg dose administration. p-RB levels were also reduced after Palbociclib (40mg/kg) and Abemaciclib (15 mg/kg) administration.

The in vivo antitumor effects of TQB3616 at doses of 60, 45, 35, 25 and 15 mg/kg were analyzed in human lung cancer cell line LU-01-0393 xenograft tumor model. The results of 21 days of administration showed that TQB3616 inhibited tumor growth in a dose-dependent trend. The T/C of 45 mg/kg, 35 mg/kg, 25mg/kg and 15 mg/kg groups were 28%, 51%, 54% and 74%, respectively, and the TGI were 99%, 65%, 62% and 32%, respectively. Compared with the solvent control, all the groups except the 15 mg/kg group had p <0.05. Drug-induced weight loss was also dose-dependent. Tumor suppression was not calculated in the highest dose group of TQB3616 (60mg/kg) because all animals lost to 20% body weight during administration and were euthanized. Mean body weight loss was less than 10% when TQB3616 was administered at doses of 35 and 25 mg/kg. Palbociclib 120mg/kg administered for 21 days resulted in a T/C of 32%, a TGI of 86%, and a slight weight loss. At 21 days of Abemaciclib 120mg/kg, T/C was 36% and TGI was 82%, p=0.001, with a slight weight loss. PD studies in this model showed that the expression levels of Rb and p-Rb were decreased after TQB3616 (35 mg/kg) Palbociclib (120 mg/kg) and Abemaciclib (120 mg/kg) administration.

Taken together, TQB3616 inhibited the kinase activity of CDK2/4/6 and inhibited the proliferation of a variety of tumor cells. The in vivo results showed that TQB3616 had significant anti-tumor activity in both human cell line MCF7 breast cancer and human non-small cell lung cancer xenografts in nude mice. The lowest dose tested in the subcutaneous xenograft tumor model of human breast cancer MCF7 cells was 5mg/kg, which was the effective dose. The inhibition of TQB3616 on RB phosphorylation downstream of CDK2/4/6 was observed in both models.

1.2.2 Results of pharmacokinetic studies

After a single intravenous injection of TQB3616 1 mg/kg in male and female SD rats, the plasma clearance (CL) was 30.5±9.07 mL/min/kg. The steady-state apparent volume of distribution (V_dss_) was 11.1±4.09 L/kg, the elimination half-life (T1/2) and the area under the time-plasma concentration curve (AUC_0-last_) were 4.20±0.343 h and 1300±385 nM • h, respectively.

A single oral administration of TQB3616 at 2, 6 or 20 mg/kg in male and female SD rats resulted in bioavailability of 45.1%, 75.5% and 95.8%, AUC_0-last_ of 1150± 385,5880 ±2520 and 25200±6970 nM • h, respectively. The peak concentrations (C_max_) were 103±30.7, 415±112 and 1070 ±141 nM, respectively, and the peak time appeared at 6.67±2.07, 9.33±2.07 and 10.0 ±2.19 hours after administration, respectively. Within the dose range of 2 mg/kg to 20 mg/kg, the systemic exposure (AUC_0-last_) of male and female rats increased more than the proportional increase of the dose (18.7 and 24.6 times in males and females, respectively), but the C_max_ increased about the same proportion of the dose (9.78 and 10.8 times in males and females, respectively).

At each dose of a single oral dose, the systemic exposure of female rats was slightly higher than that of male rats, but there was no significant difference (the ratio of female to male AUC_0-last_ at 2, 6 and 20 mg/kg doses were 1.19, 2.05 and 1.57, respectively). After 7 days of oral administration of 6 mg/kg TQB3616, there was no significant change in systemic exposure (AUC_0-24_ on day 7 in males and females was 182% and 112% of that on day 1, respectively).

After a single intravenous injection of 0.6 mg/kg TQB3616 in both male and female beagles, the plasma clearance (CL) was 32.0±7.22 mL/min/kg and the apparent volume of distribution at steady state (V_dss_) was 17.0±3.92 L/kg. The values of elimination half-life (T1/2) and area under the curve of plasma concentration (AUC_0-inf_) were 7.75±2.12 h and 729±165 nM • h, respectively.

After a single oral administration of 0.6, 2 and 6 mg/kg TQB3616 capsules in male and female beagle dogs, the bioavailability of TQB3616 capsules was 67.5%, 56.0% and 52.9%, respectively, and the systemic exposure (AUC_0-inf_) was 492±196, 1360±460 and 3860±656 nM• h, respectively. The peak concentrations (C_max_) were 33.5±10.2, 109±53.5 and 333±78.3 nM, and the peak time were 5.00±1.67, 5.33±1.03 and 5.00±1.10 h, respectively. As the dose increased from 0.6 to 2 mg/kg and from 2 to 6 mg/kg, the systemic exposure (AUC_0-inf_ and C_max)_ increased linearly in a dose-dependent manner in both female and male beagle dogs. Overall, there was a linear dose-dependent increase in systemic exposure (AUC_0-inf_) for both male and female beagle dogs across the dose range of 0.6 to 6 mg/kg.

There was no significant sex difference in TQB3616 systemic exposure (AUC_0-inf_ and C_max_) after a single oral administration in male and female Beagle dogs. After oral administration of 2 mg/kg TQB3616 for 7 consecutive days, a decrease in systemic exposure to TQB3616 (AUC_0-last_) was observed in both male and female beagle dogs compared with day 1, but not significantly so.

Under the experimental conditions of two-way permeability based on the Caco-2 cell model, it is difficult to make a clear evaluation of the permeability of TQB3616 and whether it is an efflux transporter substrate. However, on the surface, TQB3616 exhibits low permeability in Caco-2 cells and is most likely a substrate for efflux transporters.

After administration of [^14^C] TQB3616 to male and female SD rats at 6 mg/100 µCi/kg, the total radioactivity was widely distributed in the body, mainly in the gastrointestinal wall, kidney, spleen, liver and lung. The peak concentration (C_max_) of the total radioactivity in the stomach wall and testis and epididymis was reached at 0.5 h and 24 h after oral administration, respectively. The total radioactivity in the other tested tissues reached or approached C_max_ at 4 h after oral administration, which was similar to the peak time of the total radioactivity in whole blood and plasma. The total radioactivity was eliminated rapidly in vivo, and a small amount of radioactivity could still be detected at the last collection time point (72 hours), accounting for less than 0.67% of the dose.

Plasma protein binding assays showed that TQB3616 exhibited high protein binding rates (96.0-97.4% and 97.0-97.5%, respectively) in CD-1 mouse and SD rat plasma at 0.2, 2 and 10 µM tested concentrations. It showed moderate to high protein binding rates in beagle dog plasma (94.7 to 95.2%), moderate protein binding rates in cynomolgus monkey and human plasma (91.5 to 93.9% and 91.3 to 92.8%, respectively). No significant concentration dependence was found in the plasma protein binding rates at the different tested concentrations.

TQB3616 is metabolized at a moderate rate in CD-1 mice, SD rats, beagle dogs, cynomolgus monkeys, and human liver microsomes. It is metabolized in liver microsomes mainly through monooxidation, dioxidation, demethylation, deethyl, dealkylation and dehydrogenation. Compared with animal species, the percentage of specific metabolites in human liver microsomes of M10 was 1.33%, and other metabolites could be detected in animal species. TQB3616 is metabolized in hepatocytes mainly through monooxidation, deethyl, and N-dealkylation pathways. In contrast to animal species, no specific metabolites were found in human hepatocytes, and all metabolites could be detected in animal species. The main metabolic pathways of TQB3616 in rats were: 1) oxidation; 2) oxidation and pethidine ring dehiscence; Phase II is a glucuronic acid conjugate. The main biotransformation pathways of TQB3616 in dogs include dealkylation and oxidation. There was no significant sex difference in metabolism between male and female dogs.

For metabolites M1 and M4, CYP3A was the major metabolic enzyme of TQB3616. For metabolite M7, other unknown enzymes may be involved in the metabolism of TQB3616 to M7 in human liver microsomes and play a major role. There was no significant gender difference in the excretion route of TQB3616 in SD rats administered by oral gavage, and fecal excretion was the main route. TQB3616 had no inhibitory effect on CYP1A2, CYP2B6, CYP2C8, CYP2D6 and CYP3A4 (Midazolam as substrate) (IC_50_ > 50.0). There was a weak inhibition of CYP2C9 and CYP3A4 (IC_50_ = 23.0 and 30.3, respectively) with Testosterone as substrate, and a moderate inhibition of CYP2C19 (IC_50_ = 9.33). The enzyme activity and gene expression data did not show that the test product TQB3616 had a significant induction effect on CYP1A2, CYP2B6 and CYP3A4, but due to the cytotoxicity of the test product on human hepatocytes, the experimental results are for reference only.

1.2.3 Toxicological study results

In the safety pharmacological study, TQB3616 did not affect respiratory function, central nervous system, blood pressure and electrocardiogram, and had no significant or toxicological significance. Only a slight increase in blood pressure (systolic blood pressure, mean arterial pressure, and pulse pressure gradient) in beagles was observed after the administered dose of 10 mg/kg. In the single-dose toxicity (acute toxicity) study, the maximum tolerated dose (MTD) of rats was 120 mg/kg. The MTD for beagles was 200 mg/kg.

For the continuous toxicity (long-term toxicity) study, SD rats were treated with TQB3616 at doses of 10, 30, and 60→45 mg/kg/day once daily for 21 consecutive days. Harmful changes in hematology and granulocytocytic ratio were observed in female and/or male animals receiving doses ≥30 mg/kg/day. At doses from 60 to 45mg/kg/day, adverse changes in body weight/weight gain/loss, reduced food intake, and emaciation were observed, accompanied by histologic changes in the kidneys, liver, lung, small intestine (duodenum, jejunum, and ileum) and large intestine (cecum and colon), bone marrow (sternum and femur), thymus, spleen, lymph nodes (mesentery and submandibular), female reproductive system (ovary, uterus, and vagina), male reproductive system (prostate and seminal vesicle), adrenal gland, pancreas, and skin (groin). Therefore, the NOAEL level was 10 mg/kg/day for both male and female animals. At this dose level, AUC_0-24h_ after the last dose was 5740 h*ng/mL in males and 6450 h*ng/mL in females, and C_max_ was 370 ng/mL and 388 ng/mL, respectively. Because no dead or dying animals were observed at the dose of 30 mg/kg/day, the HNSTD (the highest non-severe toxic dose) was 30 mg/kg/day in both sexes under the experimental conditions. At this dose level, AUC0-24h after the last dose was 14500 h*ng/mL in males and 15600 h*ng/mL in females, and C_max_ was 755 ng/mL and 773 ng/mL, respectively.

Male and female beagle dogs were administered TQB3616 at doses of 1, 3, and 10→7.5 mg/kg/day once daily for 21 days. The dose of 10 mg/kg/day group was reduced to 7.5 mg/kg/day from week 2 due to the significant decrease in body weight and food intake observed. In the 10→7.5 mg/kg/day group, vomiting, abnormal stool (soft, watery and mucus) and poor appetite were observed in both sexes, accompanied by a significant decrease in body weight and food intake. At the end of the dosing period, deleterious changes in haematology, serum biochemistry and histopathological changes in the liver, gallbladder, lung, bone marrow (sternum) and thymus were observed. Therefore, the NOAEL level was considered to be 3 mg/kg/day under the experimental conditions. At this dose level, the AUC_0-24h_ of male and female animals after the last dose was 467 h*ng/mL and 447 h*ng/mL, respectively, and the maximum C_max_ was 53.7 ng/mL and 45.0 ng/mL, respectively.

The results of genetic toxicology test showed that TQB3616 had no mutagenic activity, no activity to induce DNA damage and chromosome aberration in rats, and no activity to induce chromosome aberration in cells.

1.2.4 Clinical study of TQB3616

As of July 2021, phase I and phase II clinical trials been conducted on TQB3616 in in China. The results of TQB3616 clinical trials are summarized as follows:

- Phase I Tolerability and Pharmacokinetics Clinical Trial of TQB3616

This was a single-arm, open-label, dose-escalation and expansion phase I clinical study. Patients with ER+, HER2- advanced breast cancer who had failed standard therapy were enrolled. TQB3616 was administered orally once daily in a 3+3 dose escalation regimen in 28-day treatment cycles. At the same time, the subjects were expanded to 10 to 12 patients in the expansion stage. The primary endpoints were safety and tolerability, MTD and dose-limiting toxicity (DLT). The secondary end points were pharmacokinetic characteristics and preliminary efficacy.

By July 2021, a total of 40 breast cancer patients had been enrolled, and the safety and tolerance of different doses had been basically completed. The study results showed that TQB3616 had a good safety and tolerability, and no DLT was observed in the dose range of 20 to 240mg, that is, the maximum tolerated dose MTD was not less than 240mg. The main adverse events were gastrointestinal reactions and hematologic toxicity, which were mainly grade 1-2. Grade 3 adverse events (≥10%) included diarrhea (5/40, 12.5%), neutropenia (5/40, 12.5%), and leukopenia (4/40, 10.0%). No grade 4 adverse events or deaths were observed.

The median age of the patients in phase I study was 52.5 years, the median number of treatment lines was 4 (2-9), 75% had received at least 2 lines of endocrine therapy, and 63% had received at least 2 lines of chemotherapy. Sixty percent of patients had at least three sites of metastases, and more than 80% had visceral metastases. The ORR of evaluable patients was 15.4% (6/39), DCR was 84.6% (33/39), and CR was 51.3% (20/39). The ORR of the recommended dose of 180mg in phase II was 25% (3/12). The median PFS was 6.4 months, and the longest treatment time was more than 18 months.

In the phase I study, the in vivo peak time T_max_ of TQB3616 was about 6 ~ 8 h, and the elimination half-life T_1/2_ was between 65 ~ 75 h. In the dose range of 20 to 240mg, C_max_ and AUC increased with the dose. There is accumulation of plasma concentration and exposure after continuous administration. Combined with safety data, once-daily dosing is supported.

- TQB3616 in combination with fulvestrant for HR positive / Phase II Clinical Trial in HER2 Negative Advanced Breast Cancer

To investigate the efficacy and safety of TQB3616 plus fulvestrant in hormone receptor-positive (HR+), HER2-negative advanced breast cancer in a single-arm, open-label, multicenter phase II trial was conducted. The study consisted of two cohorts. Cohort 1 included patients with endocrine-resistant breast cancer who relapsed during adjuvant therapy or had advanced disease progression after first-line therapy within 1 year; Cohort 2 enrolled patients with treatment-naive, locally advanced or metastatic breast cancer who had not received previous systemic therapy. The regimen was TQB3616 (180mg/qd) + fulvestrant (500mg). Until July 2021, a total of 73 subjects had been enrolled, including 38 cases in cohort 1 (endocrine resistance) and 35 cases in cohort 2 (first-line initial treatment). Twenty subjects (10 in cohort 1 and 10 in cohort 2) completed at least one efficacy evaluation, and 33 subjects completed at least one cycle of safety evaluation.

The efficacy evaluation results showed that 15 of the 20 evaluable subjects underwent only one efficacy evaluation, and the preliminary efficacy showed that 5 cases (25%) were PR, 14 cases (70%) were SD, and 1 case (5%) was PD. At present, the ORR, PFS, CBR and other data are not mature and continue to be followed up, but TQB3616 combined with fulvestrant has shown good preliminary efficacy.

Thirty-three subjects could be evaluated for safety, and all of them reported AE, but most of them were grade 1-2, which were controllable after symptomatic treatment. No new unexpected adverse reactions were observed. The overall incidence of grade 3 TEAEs was only 15.2% (5/33), mainly including neutropenia (3/33, 9.1%), diarrhea (2/33, 6.1%), leukopenia (2/33, 6.1%), vomiting (1/33, 3.0%) and fatigue (1/33, 3.0%). When TQB3616 was combined with fulvestrant at the dose of 180mg, only 2 subjects experienced dose reduction (reduce to 150mg), and 1 patient experienced drug-related serious adverse event (general fatigue). At present, the safety evaluation is still under close follow-up. Preliminary safety data analysis showed that TQB3616 180mg/qd combined with fulvestrant was well tolerated in advanced breast cancer, diarrhea and other gastrointestinal adverse reactions were controllable, and hematological toxicity was low, suggesting that this combination can be used for long-term continuous treatment.

- 1. Potential Risks and Benefits

1.3.1 Known potential risks

Due to TQB3616 plus fulvestrant is in the premarketing phase of clinical studies, and data are scarce, there are still uncertain safety risks associated with receiving the investigational drug in this trial. As with other drugs, TQB3616 and fulvestrant may be associated with some expected adverse events, as well as unexpected and even severe toxic effects.

Based on the preliminary safety information observed for TQB3616, combined with the information on similar drugs abroad, some adverse reactions to TQB3616 may occur, including but not limited to the following:

- Gastrointestinal system: diarrhea, vomiting, nausea, abdominal pain, loss of appetite, oral mucositis;
- Blood system: neutropenia, leukopenia, thrombocytopenia, anemia, lymphocytopenia, etc.
- Liver function: ALT/AST increased, bilirubin increased, etc.
- Others: fatigue, weight loss, hypertriglyceridemia, hypercholesterolemia, infection, etc. According to the post-marketing instructions, the main side effects of fulvestrant include:
- Very common (≥10%) adverse reactions: weakness, injection site reactions, elevated liver enzymes (ALT, AST, ALP), nausea, etc.
- Common (≥ 1% ≤ 10%) adverse reactions: venous thromboembolism, hot flashes, vomiting, diarrhea, anorexia, rash, urinary tract infection, allergic reactions, bilirubin elevation, headache, back pain, etc.
- Occasional (≤1%) but potentially serious adverse reactions: liver failure, hepatitis, etc.

In the process of clinical trials, investigators will pay close attention to the safety observation indicators of the subjects, make timely medical decisions to adjust the dosage regimen or actively support treatment, and manage and control the risk with reference to the clinical experience of similar drugs.

Related medical examinations during the study may also pose a risk to the subjects. More frequent imaging examinations will expose the subject to low dose radiation more frequently. However, subjects with advanced or metastatic disease usually progress rapidly, and a certain frequency of imaging examinations is also an essential step to determine whether the subject's disease is progressing.

1.3.2 Known Possible Benefits

TQB3616, a novel CDK2/4/6 inhibitor, is currently being used in the treatment of advanced malignant solid tumors. Preclinical in vitro activity data showed that TQB3616 was more potent than Abemaciclib and Palbociclib against CDK2, CDK4, and CDK6 kinases, as well as MCF-7 cell lines. Moreover, the antitumor effect in MCF-7 and other xenograft tumor models is significantly stronger than that of the reference drugs Abemaciclib and Palbociclib. TQB3616 has shown good efficacy as a single agent in the treatment of advanced breast cancer patients with multi-line progression in phase I clinical tolerability study. Among similar products abroad, CDK4/6 inhibitors combined with fulvestrant have significant efficacy in the treatment of HR-positive, HER2-negative locally advanced or metastatic breast cancer: despite different cohorts, CDK4/6 inhibitors combined with fulvestrant reduce the risk of disease progression by 40%-50%. Mortality risk was reduced by 20% to 30%. In a phase II study of TQB3616 combined with fulvestrant in advanced breast cancer, clear antitumor efficacy was also observed. Therefore, patients with previously treated HR-positive, HER2-negative breast cancer may benefit from this study.

1. Objectives and endpoints
   1. Primary objective

To evaluate the PFS of TQB3616 plus fulvestrant versus placebo plus fulvestrant in subjects with previously treated HR-positive, HER2-negative advanced breast cancer.

- 1. Secondary objectives

To evaluate the OS, ORR, CBR, DOR of TQB3616 plus fulvestrant versus placebo plus fulvestrant in subjects with previously treated HR-positive, HER2-negative advanced breast cancer.

To evaluate the safety of TQB3616 in combination with fulvestrant versus placebo in combination with fulvestrant in subjects with previously treated HR-positive, HER2-negative advanced breast cancer, including incidence and severity of AEs and SAEs, abnormal laboratory abnormalities.

- 1. Primary endpoint

PFS as assessed by the investigator.

- 1. Secondary endpoints
- Efficacy indicators
- PFS assessed by independent review committee;
- OS;
- ORR;
- CBR;
- DOR.
- Safety indicators
- Incidence and severity of AEs, SAEs and laboratory abnormalities.

1. Study Design
   1. Overall design

This is a randomized, double-blind, parallel-controlled, multicenter study. Patients were randomized in a 2:1 ratio to receive TQB3616 plus fulvestrant (experimental group) or matching placebo plus fulvestrant. Randomization is stratified according to:

1. visceral metastatic disease (yes vs. no)
2. menopausal status (pre-, peri- or postmenopausal)
3. sensitivity to prior endocrine therapy (yes vs. no).

Efficacy assessments are performed every 8 weeks (56 days) and for every 12 weeks (84 days) after 48 weeks. For patients with disease control (CR + PR + SD) and tolerable adverse reactions, the treatment can be continued until loss of clinical benefit, intolerable toxicity and the investigator consider it inappropriate to continue the treatment.

- 1. Sample size estimation

This study uses a randomized, double-blind, parallel-controlled, multicenter trial design, PFS is the primary endpoint.

The number of patients required for this trial was based on the primary end point of investigator-assessed PFS and was calculated with the use of a predefined stratified log-rank test. Assuming a median PFS of 6.0 months for placebo plus fulvestrant, we estimated that 186 events of progression or death would be required in the two treatment groups for the study to have 90% power to detect a hazard ratio (HR) of 0.60 with a two-sided significance level of α = 0.05. A total sample of 243 patients was required. Assuming an attrition rate of 15%, at least 287 patients (191 for the experimental group and 96 for the control group) were anticipated. The primary end point was to be analyzed at the interim analysis at approximately 70% maturity in the overall population when 131 events of progression or death had occurred and at the final analysis at 100% maturity when 186 events of progression or death had occurred. Type I errors were controlled using the Lan-DeMets spending function approximating O’Brien-Fleming boundary.

- 1. Interim Analyses

An Independent Data Monitoring Committee (IDMC) will be established for this study to conduct an interim analysis. The primary end point (PFS) is to be analyzed at the interim analysis at approximately 70% maturity in the overall population when 131 events of progression or death had occurred. Type I errors were controlled using the Lan-DeMets spending function approximating O’Brien-Fleming boundary with a two-sided significance level of α = 0.05:

| Number of events | Test level |
| --- | --- |
| 131 (7 0 %) | 0.01477 |
| 186 | 0.04551 |

Note: The actual nominal test level at interim analysis will be determined according to the proportion of events at that time. The nominal test level for the final analysis will be adjusted accordingly.

In actual operation, if the proportion of events exceeds 70% in interim analysis, the corresponding nominal test level will be recalculated according to the O 'Brien-Fleming α-consumption function method, as described in the IDMC charter. Based on the analysis results, the IDMC made a written recommendation to the sponsor on whether the trial is conducted or not and whether the data can be summarized in advance. The sponsor will take appropriate decisions on this study project according to the IDMC recommendations, such as terminating the trial due to safety or lack of efficacy or summarizing the application in advance due to drug efficacy, and supplementing the data after the end of the entire trial.

- 1. Methods to reduce offset

3.4.1 Randomization

This study is a multicenter, randomized, double-blind, parallel controlled trial. The randomization method is central stratified randomization, each center competed for enrollment, and the random ratio is 2:1. Subjects are randomized using a central randomization system. After the official launch of the project, the blind results are uploaded by independent statisticians. For the subjects who had signed the informed consent form and were successfully screened, the investigators are required to log in the central randomization system and input the basic information of the subjects, including the research center, the abbreviation of the subject's name, gender, date of birth, stratification factors, etc. After confirming the accuracy of the basic information and stratification factors of the subjects, the investigators or their authorized personnel underwent randomization. The central randomization system will return the randomization results for that subject, including: the randomization number and the corresponding drug number. The drug manager of the sub-center will issue the corresponding drug according to the drug number. The random number of each successfully randomized subject is uniquely and permanently identified. Patients who underwent randomization but did not receive a trial drug had their drug and drug number invalided and could not be reassigned. The monitor could log into the system to verify the basic information of the subjects and the drug use of each center, and send questions to the responsible person for the information in doubt.

Randomization is stratified according to the following three:

1. visceral metastatic disease (yes vs. no)
2. menopausal status (pre-, peri- or postmenopausal)
3. sensitivity to prior endocrine therapy (yes vs. no).

Note: Prior endocrine therapy-sensitive, defined as: disease progression after 2 years of adjuvant endocrine therapy, or disease progression after 6 months of first-line endocrine therapy. Visceral metastasis is defined as: systemic organs in the chest, abdomen and pelvis, excluding skull and musculoskeletal region.

3.4.2 Blind design

Blinding

This study uses a double-blind design, and the investigators, researchers involved in the evaluation of trial effects, data managers, statistical analysts, and subjects and their relatives or guardians are blinded to the treatment assignments. Blinded bases are generated with the use of SAS software by an independent statistician who is not associated with the final trial statistics and are uploaded to the central randomization system after the official start of the study. Blind bases are kept in a central randomization system during the study.

Emergency unblinding

- Conditions for emergency unblinding

In case of emergency (such as serious adverse events, serious complications, etc.), the subjects need rescue, and the rescue measures depend on knowing what treatment the patient receives, the blind can be unblinded urgently.

- Procedures for emergency unblinding

Online emergency unblinding is used. If necessary, the sub-site principal investigator may request online emergency unblinding for a specific subject through the central randomization system. Only the sub-site principal investigators have access to emergency unblinding.

- Independent emergency unblinding of pharmacovigilance

When SUSAR occurs, the independent pharmacovigilance specialist applies for the blind base to the system leader, who sends it directly to the pharmacovigilance specialist after the review by the system leader, who is responsible for keeping and maintaining the blind base.

- Records of unblinding

After the emergency unblinding, subsequent situations should be recorded in the corresponding original medical records/electronic case report form. The investigator should fill in the record form for emergency unblinding, and send the scanned copy to the sponsor for preservation.

- Treatment after unblinding

Once a subject is unblinded, the subject with this number will withdraw from the trial and will not be replaced. The unblinding subject should be followed until improvement or stable state is achieved.

3.4.3 Handling of randomization errors

If a subject who does not meet the study inclusion criteria and meets the exclusion criteria is randomized in error, the sponsor representative and the investigator must discuss the subject's continuation or withdrawal from the study. The sponsor will ensure that appropriate records are maintained of this determination.

1. Estimated objectives

The main clinical concerns of this study are: in subjects with HR-positive, HER2-negative advanced breast cancer defined by the inclusion and exclusion criteria, the Hazard Ratio (HR) is used as the effect size at the population level to study the efficacy of drugs through investigator-assessed PFS, regardless of early termination of treatment for any reason (an endpoint event is not reached) and under the assumption of no new antitumor therapy.

4.1 Primary estimated objective

Given that in our trial, early treatment termination reflects clinical practice, we considere the treatment strategy as the concomitant event of primary estimated objective: Strategies for early treatment termination. A hypothetical strategy is used to manage concomitant events: starting a new antitumor therapy before the PFS event. The main estimated primary objectives are defined as follows:

Target population: HR+/HER2- advanced breast cancer patients defined by inclusion and exclusion criteria;

Treatment: Subject Received TQB3616 Capsules/Placebo Capsules, Fulvestrant Injection (Qingkeyi);

Target Variables: PFS assessed by the investigator, defined as from randomization to PD or death, whichever occurs first;

Concomitant event and treatment strategy:

| Concomitant event | Treatment strategy | Notes |
| --- | --- | --- |
| Initiation of new antineoplastic therapy prior to PFS event | Imaginary strategy | If no new anticancer therapy occurs, the new anticancer therapy time point will be censored |
| Early termination of treatment | Therapeutic strategy | Ignored concomitant events and PFS time is used regardless of early discontinuation of treatment |

Population summary: Hazard ratio (HR).

Note: Early termination of treatment mainly includes the following:

1. The subject still cannot tolerate the toxicity after dose adjustment;
2. The subject requires premature termination of the investigational drug and voluntary withdrawal;
3. Other reasons that the investigator believes the subject is unable to continue the study treatment (lack of clinical benefit, worsening health condition, etc.);
4. The subject experiences a pregnancy event during the study;
5. Subjects who, in the opinion of the investigator, are noncompliant with study procedures or study drug administration, require early termination of treatment.

4.2 Secondary estimated objective 1

Target Variables: PFS assessed by independent review committee, defined as time feom randomization to PD or death, whichever occurs first;

The rest is the same as the primary estimated objective.

4.3 Secondary estimated objective 2

Target Variables: OS, defined as death from any cause from randomization;

Concomitant event and treatment strategy: Initiation of new antineoplastic therapy before the OS is the key event to be considered, and treatment strategy is adopted;

| Concomitant event | Treatment strategy | Notes |
| --- | --- | --- |
| Initiation of new antineoplastic therapy prior to OS event | Therapeutic strategy | Ignored concomitant events and PFS time is used regardless of new anti-tumor treatment. |

The rest is the same as the primary estimated objective.

4.4 Secondary estimated objective 3

Target Variables: DOR assessed by the investigator;

Target population: Patients with HR+/HER2- advanced breast cancer according to inclusion criteria who achieved clinical response;

Concomitant event and treatment strategy: With initiation of new anti-tumor therapy and premature discontinuation before DOR events as the key concomitant event . Among them, early termination of treatment is handled with the therapeutic strategy, and initiation of new anti-tumor treatment before the occurrence of the endpoint event is handled with the hypothetical strategy, that is, assuming no new anti-tumor treatment occurres.

The rest is the same as the primary estimated objective.

4.5 Secondary estimated objective 4

Target Variables: ORR assessed by the investigator;

Concomitant event and treatment strategy: The key concomitant event are new anti-tumor therapy and early termination of treatment, all of which are treated with imaginary strategies. Under this strategy, it is assumed that no new anti-tumor therapy occurs and no treatment is prematurely terminated. That is, if a subject starts a new antineoplastic therapy or prematurely discontinues treatment, subsequent efficacy evaluations will not be included in the determination of best response.

Population summary: Odds ratio (OR).

The rest is the same as the primary estimated objective.

4.6 Secondary estimated objective 5

Target Variables: Investigator-assessed CBR;

The rest are the same as secondary estimated objective 4.

1. Subject selection and withdrawal
   1. Inclusion criteria

Subjects who meet all of the following inclusion criteria can be included in this trial:

1. Voluntarily participate in this study, sign the ICF and have good compliance;
2. Age 18-75 years (calculated on the date of signing the informed consent form); ECOG PS of 0-1; predicted life expectancy of ≥3 months;
3. Postmenopausal or premenopausal/perimenopausal female patients who meet any of the following:

- Prior Oophorectomy;
- Age ≥ 60 years;
- Age < 60 years, natural menopause ≥ 12 months (no chemotherapy, tamoxifen, toremifene, or ovarian castration drugs have been administered in the past year), with follicle stimulating hormone (FSH), and estradiol (E2) levels within postmenopausal range;
- Premenopausal or perimenopausal patients may also be eligible, if willing to accept LHRH agonist therapy during the study.

1. Histopathologically confirmed HR-positive, HER2-negative, locally recurrent or metastatic breast cancer, not amenable to curative resection or radiotherapy, or clinically not indicated for chemotherapy:

- HR positive status was defined as positive (≥10%) estrogen receptor expression or progesterone receptor expression (confirmed by the pathology department of the research center);
- HER2-negative status was defined as 0 or 1+ intensity on immunohistochemical testing, 2+ intensity on immunohistochemical testing and *in-situ* hybridization-negative, or *in-situ* hybridization-negative in the absence of immunohistochemical testing (confirmed by the pathology department of the research center);

1. Eligible participants with prior endocrine therapy must meet one of the following three criteria:
2. relapse or progression during or within 1 year after completion of adjuvant endocrine therapy without subsequent endocrine therapy;
3. recurrence or progression more than 1 year after completion of adjuvant endocrine therapy, and re-progression after receiving rescue endocrine therapy;
4. progression after rescue endocrine therapy after an initial diagnosis of locally advanced or metastatic disease;

Note: ① Recurrence or progression of previous treatment should be confirmed by imaging examination;

② The time required to receive adjuvant endocrine therapy is not less than 1 year.

1. must have received no more than one line of rescue endocrine therapy or rescue chemotherapy for recurrent or metastatic disease;
2. required to have a measurable lesion according to the Response Evaluation Criteria in Solid Tumors (RECIST), version 1.1, or bone only metastasis including lytic or mixed lytic-blastic bone lesions;
3. Adequate function of the important organs as evidenced by the following:

- Blood routine examination (no blood transfusion within 7 days, no correction with hematopoietic stimulator drugs):

a) Hemoglobin (HB) ≥ 100 g/L;

b) Absolute neutrophil count (NEUT) ≥ 1.5 × 10 ^9^ /L;

c) Platelets Counts (PLT) ≥ 90 × 10 ^9^ /L.

- Blood Chemistry:

a) Total bilirubin (TBIL) ≤ 1.5×upper limit of normal value (ULN);

b) alanine aminotransferase (ALT) and aspartate aminotransferase (AST) ≤ 2. 5 × ULN. If with liver metastases, then ALT and AST ≤ 5 × ULN;

c) Serum creatinine (CR) ≤ 1.5 × ULN, or creatinine clearance (CCR) ≥ 60 ml/min.

- Coagulation function:

Prothrombin time (PT), activated partial thromboplastin time (APTT), international normalized ratio (INR) ≤ 1.5 × ULN (no anticoagulant therapy);

- Echocardiography evaluation: left ventricular ejection fraction (LVEF) is ≥ 50%.

1. Women of childbearing potential and men must agree to contraception for the duration of study treatment and 6 months after the last dose of study treatment (such as an intrauterine device, contraceptive pills, or condoms), and have a negative serum pregnancy test within 7 days prior to the study grouping and be on-lactating.
   1. Exclusion criteria

Subjects who meet any of the following exclusion criteria will not be enrolled in the study:

1. Previous pathological diagnosed HER2-positive breast cancer;
2. Bilateral breast cancer or inflammatory breast cancer;
3. Concomitant diseases and medical history:
4. Patients with other malignancies within 3 years excluding the following two conditions: other malignancies treated by single operation, reaching disease-free survival (DFS) for 5 consecutive years; cured cervical carcinoma in situ, non-melanoma skin cancer, and superficial bladder tumors [Ta (non-invasive tumor), Tis (carcinoma in situ), and T1 (tumor infiltrating basal lamina)];
5. With multiple factors affecting oral and drug absorption (e.g., inability to swallow, post-GI resection, ulcerative colitis, symptomatic/inflammatory bowel disease, chronic diarrhea and intestinal obstruction and other gastrointestinal diseases);
6. History of severe pneumonia such as interstitial lung disease;
7. Unresolved toxicity higher than CTC AE grade 1, excluding alopecia, due to any prior therapy;
8. Major surgical treatment, or significant traumatic injury within 28 days prior to the study.
9. Long-term unhealed wounds or fractures;
10. Arteriovenous thrombosis occurred within 6 months, such as cerebrovascular accident (including temporary ischemic attack, cerebral hemorrhage, cerebral infarction), deep vein thrombosis and pulmonary embolism;
11. Patients with a history of psychotropic drug abuse and unable to quit or with mental disorders;
12. Presence of any severe and/or uncontrolled disease:
13. Class II or more of myocardial ischemia or myocardial infarction, congestive heart failure according New York Heart Association (NYHA), treatable arrhythmia (including QTc ≥ 480 ms during screening period), or uncontrolled hypertension occurred within 6 months before the first dose;
14. Active or uncontrolled severe infection (CTC AE grade ≥2) or unexplained fever > 38.5°C in the 28 days prior to randomization;
15. Cirrhosis, active hepatitis*;

* Active hepatitis (hepatitis B reference: HBsAg positive, and HBV DNA detection value exceeds the upper limit of normal; hepatitis C reference: HCV antibody positive, and HCV virus detection value exceeds the upper limit of normal); note: eligible subjects with hepatitis B surface antigen positive or core antibody positive and hepatitis C patients require continuous antiviral therapy to prevent viral activation.

1. Patients with renal failure requiring hemodialysis or peritoneal dialysis;
2. History of immunodeficiency, including HIV positivity or other acquired, congenital immunodeficiency disease, or history of organ transplantation or hematopoietic stem cells transplant historian;
3. Tumor related symptoms and treatment:
4. Conditions with visceral crisis;
5. With central nervous system metastases (CNS), clinical evidence or history of carcinomatous meningitis, leptomeningeal disease;
6. Severe bone injury caused by tumor bone metastasis, including important pathological fracture and spinal cord compression within 6 months or expected to occur in the near future;
7. Received chemotherapy within 3 weeks before randomization, and radiotherapy (except palliative radiotherapy for nontarget lesions), endocrine therapy, or other antineoplastic therapy had been received within 2 weeks before randomization (the washout period is calculated from the end of the last treatment).;
8. Previous fulvestrant, everolimus, or CDK4/6 inhibitor therapy;
9. Uncontrolled pleural effusion, ascites, and pericardial effusion of equal or greater volume need to be drained repeatedly;
10. Known hypersensitivity for fulvestrant, LHRH agonists (i.e. Goserelin) TQB3616/ placebo or any of the excipients;
11. Attenuated live vaccination 28 days prior to the study or planned attenuated live vaccination during the study;
12. Participation in other clinical trials within 4 weeks prior to the study;
13. Subjects who have other serious physical or mental illness or laboratory abnormalities that may increase the risk of study participation, or interfere with study results, and who, in the opinion of the investigator, are otherwise unsuitable for this study.
    1. Withdrawal or termination criteria

5.3.1 Withdrawal criteria

A subject may withdraw from the trial at any time at his/her own , or for safety or administrative reason s by the investigator or sponsor, or because the subject is unable to comply with the treatment and visits protocol.

Reasons for subject withdrawal include:

1. withdrawal of informed consent by the subject;

2. death;

3. lost to follow-up;

4. the sponsor terminates the study;

5. other conditions that the investigator believes are necessary to withdraw from the study (such as the subject's loss of the ability to freely express his/her wishes due to imprisonment or isolation, etc.).

In addition, subjects may continue to be followed after discontinuing study intervention, particularly for purposes of collecting safety, survival, and efficacy study endpoints, as applicable. Requests for separate documentation for subject discontinuation or subjec withdrawal may be considered. In addition, there should be a dedicated page in the medical file and eCRF to record the date of study discontinuation or subject withdrawal and detailed potential reasons.

5.3.2 Termination criteria

Termination does not constitute withdrawal from the study. Subjects who terminate trial treatment must continue to complete the remaining visits as required by the protocol. The study treatment must be terminated if the subject meets any of the following criteria:

1. the subject requests termination of investigational drug treatment;

2. efficacy evaluation meeting the criteria for disease progression confirmed by the investigator (according to RECIST 1. 1), unless the investigator believes that the subject can continue to benefit and meets the criteria for continued treatment after disease progression;

3. pregnancy events during the study;

4. after dose adjustment, the subject is still unable to tolerate toxicity, or adverse events, laboratory abnormalities or concurrent diseases occur, etc., and continued participation in the study is not in the best interest of the subject as judged by the investigator;

5. the sponsor terminates the study;

6. Subjects who, in the opinion of the investigator, are noncompliant with study procedures or study drug administration should consult with the sponsor for instruction on handling the subject;

7. Other reasons that the investigator believes that study treatment cannot be continued (e.g., general deterioration of the subject's health status, inability to continue study treatment);

5.3.3 Procedures for withdrawal or termination

If a subject is withdrawn from the study or terminated study treatment, the following actions are taken:

- The reason (s) must be documented in the medical file and eCRF;
- An End of Treatment (EOT) visit should be performed. If some examination items are completed within 7 days before withdrawal, the above examination does not need to be performed at the EOT visit;
- Subjects must be followed for safety for no less than 28 days after the EOT visit or until the drug related toxicities resolve, return to baseline, or are deemed irreversible, whichever the longest;
- Efficacy follow-up: for subjects without disease progression but without death, imaging evaluation should be continued according to the planned frequency and time until the subjects starts new anti-tumor treatment or disease progression. Every effort should be made to obtain imaging evidence of disease progression in such patients;
- Survival status will be tracked and documented as specified in the protocol.

5.3.4 Lost to follow-up

Subjects are considered to be lost to follow-up if they do not return for scheduled trial site visits and are unable to be contacted by site staff on at least three occasions. Contact methods include: phone, fax, text, social media tools, email, etc. All these attempts to contact the subject should be documented in the subject's medical record or study file. If death is determined, the study center will obtain information on death and the cause of death to the extent possible, such as from public sources such as community health registries and databases. If all attempts have been made and subject status is not available, the investigator should report the date the subject is last known to be alive and record it in the subject's medical history.

1. Investigational drugs
   1. Basic information

TQB3616 Capsule

Strength: 60 mg/capsule, 50 mg/capsule

Storage: Protected from light, sealed, store below 25℃

Route: Oral

Placebo capsules (TQB3616 Simulant)

Strength: 0 mg/capsule

Storage: Protected from light, sealed, store below 25℃

Route: Oral

Fulvestrant Injection

Specification: 5mL: 0.25g

Storage: Sealed, protected from light, storeat 2 ~ 8℃

Route: Intramuscular

- 1. Drug Management

6.2.1 Dosing Regimen

TQB3616 /Placebo:

180 mg /0 mg, oral, QD for a 28-day cycle. It is recommended to take the drug at a fixed time every day; if there is missed dose during medication and the time interval between this dose and the next dose is less than 10 hours, the patient would not be redosed.

Fulvestrant:

500 mg, administered as an intramuscular injection, on day 1 and 15 of cycle 1 and day 1 of subsequent cycles.

6.2.2 Dose Modification

(1) General Principles

- The severity of AEs will be graded according to the National Cancer Institute Common Terminology Criteria for Adverse Events (NCI-CTCAE) v5.0 grading system;
- When severe toxicity of different severity levels occurs simultaneously, adjustments should be performed according to the highest level observed;
- Toxicities due to treatment should be recovered to at least ≤ grade 2 or baseline (except alopecia) before starting treatment in any cycle, otherwise dose delay is required.

(2) Dose adjustment

If a dose reduction is required for TQB3616/Placebo, treatment with a reduced dose will continue in subsequent cycles. No more than 2 down-reduction should be taken by the subject, which should be performed at 30 mg per dose reduction and no more than one level reduction was allowed. Re-escalation is not allowed after a dose reduction.

Dose Level adjustment of TQB3616/Placebo

| Initial level | 180 mg |
| --- | --- |
| Dose Level -1 | 150 mg |
| Dose level-2 | 120 mg |

No dose adjustment of fulvestrant is allowed, and delayed administration of fulvestrant is allowed due to toxicity. If fulvestrant is permanently teminated due to toxicity, TQB3616/placebo may be continued at the discretion of the investigator.

In the event of adverse events related to TQB3616/placebo, TQB3616/placebo should be adjusted according to the table below. In the event of toxicities related to investigational drugs during clinical operations, the investigators should consider the benefit/risk ratio of the subjects and consider that they cannot follow the procedures listed in the table below or encounter situations not listed in the table, and the investigators should deal with them according to their clinical manifestations and communicate with the sponsor if necessary.

Dose adjustment criteria of TQB3616/Placebo

| Grade | Recommendations for dosing adjustment and management |
| --- | --- |
| Grade 3 hematology toxicity  (without complication) | 1. Suspension administration, symptomatic treatment until toxicity recovers to ≤ grade 2;  2. Maintain the original dose or reduced by 1 dose level when re-administration at the discretion of the investigator. |
| Grade 4 hematologic toxicit ;  Grade 3 hematologic toxicity with infection or fever ≥ 38.5 ° C | 1. Suspension administration, symptomatic treatment until toxicity recovers to ≤ grade 2;  2. Reduced by 1 dose level when re-administration. |
| Grade 2 non hematology toxicity | 1. Immediate symptomatic treatment, suspension administration if failure to improve despite optimal symptomatic treatment until toxicity recovers to ≤ grade 1;  2. Maintain original dose. |
| Grade 3 non hematology toxicity | 1. Suspension administration until toxicity recovers to ≤ grade 1;  2. Reduced by 1 dose level when re-administration. |
| Grade 4 non-hematological toxicity | 1. Suspension administration until toxicity recovers to ≤ grade 1;  2. Permanently discontinue the treatment subsequently, or reduce the dose by 1-2 dose levels as judged by the investigator re-administration (if the investigator considers it necessary to reduce the dose by 2 dose levels, it is necessary to communicate with the sponsor to reach a consensus). |

6.2.3 Drug Management

In accordance with the requirements of GCP, all the investigational drugs shall be kept, distributed and returned by specially-assigned person in the Study site.

Used and partially used drug containers, infusion bags and syringes may be destroyed on site according to the instructions and operating procedures established by the study site and local authorities. All destruction should be documented. Unused drug was returned to the sponsor.

Complete records are required for drug distribution, recovery and other drug management processes. Returned drug was monitored periodically by the sponsor. The monitor regularly checked the use of the drug and the records.

6.2.4 Drug storage

The study site shall record the maximum and minimum temperatures for each working day at the storage location of all study drugs. The logging cycle begins with the receipt of the study drug and continues until all study drugs have been recovered.

Study sites with continuous temperature monitoring systems shall also have traceable monitoring logs for archival purposes and shall regularly inspect the temperature monitoring devices and storage devices (e.g. refrigerators) to ensure proper functioning.

In case of temperature deviation, it should be reported to the sponsor upon discovery. The research center should take active actions to bring the product to the storage conditions described in the label as soon as possible and, at the same time, report the temperature deviation and the measures taken to the sponsor. The study drug affected by the temperature deviation must be stored in an environment that meets the storage conditions of the drug until permission is received from the Sponsor to proceed with its use.

- 1. Concomitant medication and treatments

The start and end time points for collection of concomitant medication and treatment are specified as follows:

| Recordkeeping time | Recordkeeping requirements |
| --- | --- |
| Within 28 days before the first dose (the first day) | All medications or meaningful nondrug treatments: generic name, doses, the reason for treatment with this medication, start and stop date of this medication, or whether this medication is continued at study enrollment |
| From the first dose of study drug until withdrawal/termination | All medications or meaningful nondrug treatments |
| Withdrawal/termination until 28 days after the last dose or initiation of other antitumor therapy, whichever come first. | All medications related to AE/SAE disposition |
| Post-treatment phase (28 days after the last dose or initiation of other antitumor therapy) | All treatments relevant to the target indication |

6.3.1 Prohibited concomitant medications

Anti-tumor indications approved by NMPA, including modern traditional Chinese medicine preparations and immunomodulators (including compound cantharis capsule, Kangai injection, Kanglaite capsule/injection, Aidi injection, Brucea javanica oil injection/capsule, Xiaoaiping tablet/injection, cinobufacin capsule, etc).

During the trial, all patients were prohibited from receiving any local treatment for the lesion, including surgery and radiotherapy (except local palliative radiotherapy), and other systemic anti-tumor treatments such as chemotherapy, molecular targeted therapy, hormone therapy, and immunotherapy.

6.3.2 Concomitant medication allowing with caution

Preclinical studies have shown that CYP3A4 is the major metabolic enzyme of TQB3616. Concomitant use of strong inducers or inhibitors of CYP3A4 should be avoided unless deemed necessary by the investigator, in which case patients must be closely monitored for any decrease in efficacy or increase in toxicity of the investigational drugs due to drug interactions.

CYP3A4/5 inducers include, but are not limited to the following drugs: rifampin, rifabutin, rifapentine, dexamethasone, phenytoin, carbamazepine or phenobarbital. Strong inhibitors of CYP3A4/5 include but are not limited to the following drugs: ketoconazole, itraconazole, clarithromycin, voriconazole, telithromycin, saquinavir, ritolavir, etc.

6.3.3 Permitted concomitant medications

Subjects could receive bisphosphonates for bone metastases during treatment. Palliative treatment of local non-target lesions causing significant symptoms, such as bone metastases where pain is not effectively controlled by systemic therapy or local analgesia, can be considered. Local palliative small area radiotherapy or surgery, and treatment of pleural and ascites can be considered, provided that the following conditions are met:

- These lesions were known to be present at the time of enrollment;
- Subjects who require local treatment due to aggravation of symptoms during the study. Whether there is disease progression should be judged by the investigator;
- Subjects with disease progression must meet the criteria for continued treatment after progression;
- Locally treated lesions cannot be target lesions;
- While the subject is receiving palliative topical therapy, trial medication should be withheld until the end of the palliative recovery phase.

Palliative topical therapy should be discussed with the sponsor prior to initiation. The palliative care should be recorded in detail in the medical documents and eCRF, including treatment date, site, treatment method and dose, adverse reactions, etc.

1. Trail procedures
   1. Screening Visit

Subjects must sign an ICF before they can undergo any of the study-specified screening procedures. If routine tumor imaging assessments have been performed prior to the signing of the ICF, as long as these computed tomography (CT) or magnetic resonance imaging (MRI) were completed within 28 days (42 days for bone scans) before the start of study treatment, at the same study site, and were eligible for study evaluation, there was no need for repeat CT or MRI or bone scans during the screening period. Assessments within 28 days before first dose:

1. Signing the ICF;
2. Demographic data: age, gender, occupation, ethnicity, correspondence address and telephone number etc.;
3. Medical history: including but not limited to the following items, detailed tumor history and treatment history should be recorded:

- Tumor diagnosis: Primary and recurrent lesions, date of pathological diagnosis of metastases, pathological type, pre-enrollment clinical diagnosis and TNM stage, metastatic sites, and disease progression or recurrence date after the last treatment (Note: In case only liver metastases exist except for bone lesions, liver metastases must be pathologically cinfirmed. In principle, other sites also need to be re-sampled for pathology or provided pathological results within 2 years before informed consent. In case of difficult sampling of recurrence or of metastases, discuss with the sponsor if the pathological results of the primary lesion coule be used)
- Most recent Immunohistochemical results: ER/PR, HER2, Ki-67, etc.;
- Menopausal status;
- History of surgical treatment: date of surgery and name of surgery;
- History of radiotherapy: start and stop time, radiotherapy site, total dose, best effect;
- History of endocrine/chemical/targeted anti-tumor therapy (including neoadjuvant and adjuvant therapy): the name of the drug or regimen used, dose, start and stop time, cycle, tolerance, best response, whether treatment failure or progression and its date;
- History of other anti-tumor treatment: start and stop time, treatment details, best response;

1. Other prior history:

- History of concomitant diseases: record the history of diseases within 5 years prior to signing ICF, such as diabetes and hypertension;
- Allergic history: allergen;
- Concomitant diseases and abnormalities: Clinically significant abnormalities that are ongoing at screening will be considered concurrent medical conditions or symptoms. Observation continued during the study after adjudication/diagnosis by the investigator.

1. Imaging examinations (CT or MRI): PET examination is not a routine imaging assessment, and the contrast-enhanced CT or MRI examination and assessment sites before medication must include the neck, chest, abdomen and pelvis. For subjects with bone metastasis, whole-body bone scan should be performed (within 42 days prior to start of study drug is acceptable). Imaging should be performed at all suspicious lesion sites. Brain MRI and bone scan are required to be performed during the screening period (subjects without bone metastases during the screening period are examined at the discretion of the investigator based on clinical indications).
2. Concomitant medications, adverse events.

- **Assessments within 14 days before first dose:**

Virological examination: including HIV test, HBV tests (quantitative detection of HBV DNA if HBsAg is positive), HCV antibody test (HCV RNA test if HCV antibody is positive);

Echocardiogram;

- **Assessments within 7 days before first dose:**

1. Physical examination, including: general condition, skin mucosa, head, neck, chest, abdomen, spine /extremities, nervous system, lymph nodes, etc.;
2. Vital Signs: pulse rate, blood pressure, body temperature, respiration, etc. All vital signs should be measured with the subject at rest, if the body temperature is measured on the same day more than 2 times, record the highest result;
3. Height (at screening only), Weight, ECOG score;
4. Serum pregnancy test: HCG test, only for women of childbearing potential;
5. Hormone level testing (except for eophorectomy or aged > 60);
6. Tumor markers (CA15-3 and CEA);
7. 12-Lead ECG;
8. Blood routine, Blood biochemistry, Urine routine, stool routine, thyroid function, coagulation function, Lipase, amylase, etc.;
   1. Treatment period visits

In case of dose delay, the corresponding examination items at scheduled visits should be consistent with the actual administration time.

Cycle 1, C1D1:

- Study drug administration;
- Concomitant medications and adverse events.

Cycle 1, C1D15 (± 1 day):

- Physical examination, body weight;
- Vital signs;
- Hematology, blood biochemistry and urinalysis;
- 12-Lead ECG;
- Concomitant medications and adverse events.

Cycle 1, C1D28 (± 3 days):

- Physical examination, body weight;
- Vital signs;
- Hematology, blood biochemistry, urinalysis and stool routine examination;
- 12-Lead ECG;
- Concomitant medications and adverse events.

Subsequent even treatment cycles, C2nD28 (± 3 Days):

- Physical examination, body weight;
- Vital signs;
- ECOG score;
- Hematology, blood biochemistry, urinalysis, stool routine, coagulation function, thyroid function, amylase, lipase and hormone levels;
- 12-Lead ECG;
- Imaging examinations;
- Tumor markers;
- Concomitant medications and adverse events.

Subsequent odd cycles, C2n+1 D28 (± 3 Days):

- Physical examination, body weight;
- Vital signs;
- Hematology, blood biochemistry, urinalysis and stool routine examination;
- 12-Lead ECG;
- Concomitant medications and adverse events.
  1. End of treatment (EOT) Visit

When a subject meets any of the reasons for the end of study treatment, relevant examination procedures for the EOT visit are required. If some tests are done within 7 days before treatment withdrawal, they do not need to be done at the EOT visit. The following tests/collect the following information are required for EOT visit:

- Physical examination, body weight;
- Vital signs;
- ECOG PS;
- Hematology, blood biochemistry, urinalysis, stool routine, coagulation function, thyroid function, amylase, lipase and hormone levels;
- 12-Lead ECG;
- Imaging examinations;
- Tumor markers;
- Concomitant medications and adverse events.
  1. Follow-up visits

The follow-up visits begin after the EOT visit.

The safety follow-up visit is the interval between the EOT visit and the scheduled follow-up visit, which is to occur 28 days (±7 days) after the EOT visit or, if no EOT visit is performed, 28 days (±7 days) after the last dose of the study drug. Adverse events and SAEs have to be reported until at least 28 days after the last dose of study drug, the date of the follow-up visit, or until toxicity resolved, returned to baseline levels, or was deemed irreversible, whichever is longer. Reasonable efforts should be made to have the subject return to the study center for a follow-up visit to report all AEs that occur during that time. If a subject is scheduled to start a new anticancer therapy before the end of the safety follow-up period, the safety follow-up visit is to occur before the initiation of a new anticancer therapy. Once a new anticancer treatment is initiated, the subject enters the survival follow-up period.

Efficacy follow-up: Subjects who terminated the study drug for non-disease progression or non-death reasons continue to receive efficacy follow-up visit after the end of the study drug, and tumor imaging evaluation is performed according to the time point specified in the protocol until the subject's disease progression, initiation of other anti-tumor drugs or death (whichever came first). The time of each follow-up, the results of tumor imaging evaluation and other anti-tumor treatment information are recorded in detail.

The non-death subjects who were followed up for safety and efficacy (whichever is completed later) begin to enter the survival follow-up visit. Data on survival (date and cause of death) and post-study treatment (including treatment received) are collected at least once every 12 weeks (±7 days) and post-study treatment (including treatment received) sion, initiation of other anti-tumor drugs or death (whichever came firstompletion of safety and efficacy follow-up visits (whichever is completed later). Until death, loss of subjects to follow-up, or the end of OS data collection, whichever come first. The investigators could follow up the subjects themselves, family members or doctors by telephone to collect whether the subjects had taken other anticancer treatment before the follow-up. If other therapies are used, it is important to document the treatment regimen and the number of cycles and outcomes. The patients are followed up until death, and the cause and specific time of death are recorded.

- 1. Unscheduled Visit

Unscheduled visits may be performed at any time at the discretion of the investigator and appropriate clinical and laboratory tests may be performed based on AEs or other findings.

1. Efficacy evaluation
   1. Evaluation Frequency

During the study, the time points of tumor assessment will not be changed due to dose regimen adjustment, but will be calculated from the day of the first dose. Efficacy will be assessed every 8 weeks (± 7 days) and every 12 weeks (± 7 days) since 48th weeks until the radiologically confirmed disease progression. The frequency of radiographic assessments is independent of dose delays and/or dose interruptions. Additional imaging could be performed when clinically indicated. The evaluation during screening have to include noncontrast plus contrast-enhanced CT or MRI of the neck, chest, abdomen, and pelvis. Imaging should be performed at all suspicious sites. Unenhanced and contrast-enhanced MRI of the head and bone scans are required during the screening period (for subjects without bone metastases during the screening period, the study physician determined according to clinical indications). If contrast-enhanced MRI could not be performed because of contrast allergy or other reasons, contrast-enhanced CT could be performed. The same imaging algorithm have to be used for each subject throughout the study

- 1. Evaluation criteria

Imaging evaluation is performed according to RECIST 1.1.

- 1. Requirements for image evaluation

The methods of radiographic assessment (CT or MRI) will be determined by the investigator. However, the assessment methods, machines, and technical parameters should be consistent throughout the study period. Contrast media is required for subjects without contraindications. For subjects who had received a radiographic assessment within 28 days (42 days for bone scan) before the first dose using the same procedure in the same center, the radiographic assessment could be used as the baseline data in this trial. The baseline tumor assessment should include enhanced CT or MRI of the chest, abdomen, and pelvis (except for subjects with an allergy to contrast media). During screening, brain examination (contrast-enhanced MRI) and bone scanning (for subjects without bone metastases during screening, determined according to clinical indicationst by study physician) should performed. Imaging should be performed at all suspicious sites. For subjects with bone metastases, the lesion should be followed. Subjects with bone metastases at baseline do not need to be reviewed at each tumor assessment if they do not have clinical worsening during treatment. In the case of clinical worsening, patients should be reviewed promptly, and repeat bone scans are generally recommended every 24 weeks. In cases in which disease progression was suspected before the next scheduled evaluation, an unplanned tumor evaluation was performed. Imaging during the trial was recommended to include CT or MRI of the neck, chest, abdomen, pelvis, and other areas with lesions, with additional imaging of suspicious areas during the trial.

Timely imaging is required when participants discontinued study treatment for any reason (±28 days, or no repeat examination was required when they were discharged from the study group if the previous examination was not more than 4 weeks after discontinuation of treatment). Imaging is also performed when disease progression is suspected (e.g., worsening symptoms) and when the subject withdrew from the study (if imaging had not been completed in the previous 4 weeks).

Tumor assessment should be performed on an ongoing basis in all subjects according to the study protocol and should not be affected by drug interruptions or delays. Subjects were reported as symptomatic worsening if they needed to discontinue study treatment because of worsening general health but there is no objective evidence of disease progression at this time. Even after drug discontinuation, objective disease progression (confirmed on imaging) is documented, as required by the protocol.

- 1. Validation process of PD

The efficacy assessments for this study are divided into site investigator assessments and independent review committee (IRC) assessments. During the study, each study site performe efficacy assessment in combination with clinical conditions. Whether the subjects continued medication is assessed and determined by the authorized investigator in the study site.

Blind independent imaging assessment will be performed by the IRC, IRC accessed PFS according to ECIST1.1 criteria is used as a secondary endpoint.

Further details regarding the IRC assessment can be found in the "Central Imaging Operations Manual" provided by the sponsor.

- 1. Evaluation indicators

Progression-free survival (PFS): defined as the time from randomization to objective disease progression or death due to any cause, whichever occurs first.

Overall survival (OS): defined as the time from randomization to death due to any cause.

Objective response rate (ORR): defined as the percentage of subjects with complete response (CR) or partial response (PR) per RECIST 1.1 as determined by the investigator.

Time to disease response (DOR): For a best response of complete response (CR) or partial response (PR) subjects, defined as the time from the date of first documented tumor response to the date of first documented disease progression or death due to any cause, whichever occurs first.

Clinical benefit rate (CBR): The proportion of all randomized subjects with a best overall response (BOR) of complete response (CR), partial response (PR), and stable disease (SD) of 24 weeks or more according to RECIST 1.1 criteria.

1. Safety evaluation
   1. Adverse events

An adverse event (AE) is any untoward medical occurrence in a subject administered the investigational drugs and which may manifest itself by symptoms, signs, disease, or laboratory abnormalities and which do not necessarily have a causal relationship with the investigational product.

Any untoward medical occurrence or initiation of treatment for a new target indication (whichever occurs first) that occurs from the time the subject signs the ICF through 28 days after the last dose, regardless of causal relationship with the investigational drug, will be considered an adverse event in this trial.

- 1. Evaluation of Adverse Events

The nature and severity of adverse events were assessed according to the National Cancer Institute Common Toxicity Criteria (NCI-CTC v5.0).

Adverse event terms not included in NCI CTCAE v5.0 were graded according to the following CTCAE grading principles:

| Severity | Clinical description |
| --- | --- |
| Grade 1 | mild; no symptoms or mild signs; only clinical or diagnostic observation; no medical stem is needed |
| Grade 2 | moderate; the need for minimal, local or non-invasive treatment; age-appropriate functions of daily living (e.g., cooking, shopping, using the telephone, and managing money) are limited^*^ |
| Grade 3 | severe or clinically significant but not immediately life-threatening; hospitalization or prolonged hospitalization; disabling; the ability to perform activities of daily living (e.g., bathing, dressing and undressing, eating, toileting, and taking medications) was limited but not bedridden^**^ |
| Grade 4 | resulting in life-threatening consequences; urgent treatment is required |
| Grade 5 | Death related to AE |

- 1. Records of adverse events

During the reporting period of AEs, the investigators are required to record any AEs, including SAEs, in the CRF/eCRF. For the report of AEs, the investigators are required to use the correctly standardized medical terminology rather than colloquialism and abbreviations. The start date, severity grade as per NCI-CTCAE v5.0, stop date, causality to study drugs, effects on the trial, concomitant therapy, and recovery will be recorded.

Diagnosis vs symptoms and signs

A diagnosis (if known) should be recorded on the CRF/eCRF rather than individual signs and symptoms (e.g., record only liver failure or hepatitis rather than jaundice, asterixis, and elevated transaminases). However, if a constellation of signs and/or symptoms cannot be medically characterized as a single diagnosis or syndrome at the time of reporting, each event should be recorded on the CRF/eCRF as AEs. If a diagnosis is later established, it should be updated on the CRF/eCRF.

Adverse events secondary to other events

In general, AEs occurring secondary to other events (e.g., cascade events or clinical sequelae) should be identified by their primary cause, except for severe or serious secondary events. However, medically significant AEs occurring secondary to an initiating event that are separated in time should be recorded as independent events on the CRF/eCRF. All AEs should be recorded separately as primary or secondary events if it is unclear as to whether the events are associated.

Continuous, intermittent, or single AEs (frequency of adverse events)

Continuous AE extends to continuously, without resolution between cycles/courses, such as the upper respiratory tract infection lasting 5 days. The event must only be reported once. For severity grade, the highest severity grade should be recorded.

Intermittent AE refers to the changes or relief of symptoms, signs or laboratory tests during the whole process, but without clinically significant outcomes, such as nausea and vomiting lasting for several days with the intermittent resolution, and persistent hypertension with intermittent resolution. The event must only be reported once. For severity grade, the highest severity grade should be recorded.

Single AE is one that occurs independently or only once during therapy, such as an accident in which a subject falls; or vomiting that a subject only experiences once during the trial. The event must only be reported once on the CRF/eCRF.

It should be noted that these above events are recurrent after clinically significant resolution and meanwhile have no course continuity with the former, each recurrence of an AE should be recorded separately on the CRF/eCRF.

Laboratory abnormalities or vital signs

Laboratory test results will be recorded on the laboratory results pages of CRF. Not all abnormal laboratory tests and vital signs will be reported as AEs. Investigators have the responsibility to review all laboratory findings and vital signs. Medical and scientific judgment should be exercised in deciding whether an isolated laboratory abnormality should be classified as an AE. Any abnormalities that meet one or more of the following conditions for clinical significance will be reported as AEs:

- Accompanied by clinical symptoms;
- Leading to a treatment change (e.g., dose modification, interruption, or termination);
- Requiring medical intervention or the change of concomitant therapy (e.g., concomitant medication, new treatment, treatment interruption, termination, or any other change);
- Have significant clinical significance as judged by the investigator.

If a clinically significant laboratory abnormality is a sign of a disease or syndrome (e.g., increased ALT/AST and bilirubin caused by hepatic dysfunction), only the diagnosis should be recorded on the CRF/eCRF (hepatic dysfunction). Otherwise, the abnormality should be recorded along with a descriptor indicating if the test result is above or below the normal range. If the abnormalities have corresponding standard clinical terms, the standard clinical terms should be recorded (e.g., an increase in blood potassium to 7.0 mmol/L should be recorded as hyperkalemia).

Death

For reporting death events, the death due to AE should be recorded as the single medical concept on the CRF/eCRF and reported as SAE. If the cause of death is unknown, “unexplained death” should be recorded on the CRF/eCRF, reported as SAE provisionally, and confirmed by further investigation. If the cause of death later becomes available, the “unexplained death” should be replaced by the established cause of death. if the death is due to tumor progression, it should not be recorded and reported as an AE/SAE.

Pre-existing medical conditions

A pre-existing medical condition should be recorded as an AE only if the frequency, severity, or character of the condition worsens (excepte the worsening of the studiedd isease) during the study. When recording such events, it is important to convey the concept that the preexisting condition has changed by including applicable descriptors (e.g., more frequent headaches, and aggravated hypertension)

Hospitalization or prolongation of existing hospitalization

The following conditions leading to hospitalization or prolongation of existing hospitalization are not classified as SAEs:

- The hospitalization or prolongation of existing hospitalization required by protocol (e.g., administration and efficacy evaluation);
- An elective hospitalization for a pre-existing condition unrelated to the study indication. For example, the planned surgery or treatment before study or the scheduled surgery or treatment after enrollment. However, hospitalization for surgery or treatment due to disease worsening (surgery or treatment in advance) will be classified as SAEs.

Surgery

If the disease for surgery is definite, the disease should be recorded as AE rather than surgery (e.g., for subjects who underwent inguinal herniorrhaphy, AE is inguinal hernia rather than inguinal herniorrhaphy). Otherwise, the surgery should be recorded as AE (e.g., for subjects who underwent abbreviated laparotomy, abbreviated laparotomy is AE).

Pregnancy

If a female subject becomes pregnant during the trial, the investigators should be informed immediately. Investigators are required to report to the sponsor within 24 hours of learning of its occurrence. Pregnant subjects should immediately stop using the study drug. The investigator should counsel the patient, and discuss the risks of continuing the pregnancy and the possible effects on the fetus. Pregnant subjects will be monitored until the end of pregnancy. All pregnancies within 30 days of the last administration will be reported to the investigators.

Both induced and spontaneous abortions should be reported as SAEs. Any congenital anomalies/birth defects in infants born to female subjects or female partners of male subjects who had taken study drugs should be reported as SAEs.

Disease progression

If the observed progression is confirmed to be consistent with the expected pattern of primary tumor progression, it will not be considered an AE. The hospitalization due to this progression will also not be considered an SAE. If symptoms cannot be confirmed to be caused by tumor progression, or consistent with the expected pattern, the events will be considered an AE or an SAE.

- 1. Follow-up of adverse events

Investigators are required to follow up all AEs until any of the following occurs:

- AEs resolve or improve to baseline;
- No further remission will be expected by the investigators;
- Death;
- Lost to follow-up;
- AEs that are not related to the study treatment confirmed by the investigators;
- Starting new antitumor treatment;
- End of clinical or safety data collection, or the database lock.

The outcomes of each AE (including the date of resolution and death) need to be recorded in the CRF/eCRF.

- 1. Causality to Study Drugs

Investigators are required to assess the causality of AEs to study drugs, according to the following 5 criteria:

(1) Whether the administration time and the suspected AEs exhibit a reasonable relationship;

(2) Whether the suspected AEs fulfill the criteria for the typical reactions of the drug;

(3) Whether the suspected AEs can be explained by the effects of the combined drug, patient’s clinical condition, or other therapies;

(4) Whether the suspected AEs disappear or are mitigated after drug discontinuation or reduction;

(5) Whether the same AEs recurred after repetitive administration of the study drugs.

|  | 1 | 2 | 3 | 4 | 5 |
| --- | --- | --- | --- | --- | --- |
| Definite | + | + | - | + | + |
| Probable | + | + | - | + | ? |
| Possible | + | + | ± | ± | ? |
| Unlikely | + | - | ± | ± | ? |
| Unrelated | - | - | + | - | - |

Note: +, Yes; -, No; ±, probably Yes or No; ?, unknown.

AEs will be calculated as the sum of definitely-related, probably-related, and possibly-related events.

- 1. Serious adverse event

AEs that meet one or more of the following criteria are classified as SAE: death, life-threatening events (imminent danger of death), in-patient hospitalization or prolongation of existing hospitalization, a persistent or severe disability or incapacity, congenital anomalies/birth defects, or any AEs that, based upon appropriate medical judgment, may jeopardize the subject and may require medical or surgical intervention to prevent one of the outcomes listed above. Pregnancy in patients or their spouses will be reported as SAEs to the sponsor or their representative.

Disease progression (including signs and symptoms of progression) under the trial will not be reported as an SAE, but death due to disease progression during the trial or safety reporting period will be reported as an SAE. Hospitalization due to signs and symptoms of disease progression will also not be reported as an SAE. During the trial or safety reporting period, events leading to death must be reported as SAE in dead subjects.

- 1. Management of Serious Adverse Events

Any SAE, whether or not related to the study drug, occurring must provide the written report to the sponsor or their representative (PV@cttq.com) within 24 hours of being aware of its occurrence, followed by the written follow-up report in detail. For death events, the investigators have to provide the documents (e.g., autopsy and medical report) to the sponsor and ethics committee. The sponsor is required to immediately assess the severity of SAEs, their causality to the study drug, and whether it is an expected event. For suspected unexpected SAEs, the sponsor should quickly report to all participating investigators, clinical trial institutions, and the ethics committee, as well as the Drug Administration. Investigators are also required to inform these suspected unexpected SAEs of the ethics committee. Suspected and unexpected serious adverse events should also be reported by the sponsor to National Mecical Products Administration and local Commission of Health.

1. Source Documents and Obtaining Source Data/Documents

In accordance with ICH E6, applicable regulations, and institutional requirements for the protection of personal information, each study site must maintain appropriate records related to the treatment and research related to this study. As part of Chia Tai Tianqing Pharmaceutical Group CO., LTD funding or participation in the study, each site will permit the sponsor or its authorized representative and regulatory agencies to inspect (or copy if permitted by law) the clinical records for quality review, audits, and safety, study progress and data validity evaluations.

The raw data are all information necessary for the reconstruction and evaluation of the clinical study and are original records of clinical findings, observations, or other activities. Examples of such source documents and data records include, but are not limited to, hospital records, laboratory notes, memoranda, subject diary cards, pharmacy dispensing records, recording of consultation meetings, recorded data from automated instruments, copies or transcriptions certified after verification as being accurate and complete, microfiches, photographic negatives, microfilm or disks, x-rays, and subject files and records kept at the participating pharmacy, at the laboratories, and at medico-technical departments.

Source documents are able to demonstrate the existence of the subject and substantiate the integrity of the data collected. Source documents are archived at the investigator's site.

Data transferred to the eCRF from source documents must be consistent with the source documents and any discrepancies must be explained. Depending on the trial, the investigator may need previous medical records or transfer records, or may need current medical records.

The investigator/institution will permit trial-related inspections, audits, IRB/IEC review, and regulatory inspection, providing direct access to all relevant source data/documents. ECRFs/CRFs and all source documents, including disease records and copies of laboratory and medical test results must be available at all times for inspection by the sponsor's clinical research associates, auditors, and health authorities. The CRA and auditor may review all eCRFs/CRFs and written informed consents.

1. Quality control and quality assurance

The sponsor and investigator should establish their own quality assurance system, fulfill their respective responsibilities, strictly follow the clinical trial protocol and adopt the corresponding standard operating procedures to ensure the quality control of the clinical trial and the implementation of the quality assurance system.

- 1. Quality assurance of clinical trial process

Before the trial initiation, the investigators should be trained on the trial protocol so that the investigators can fully understand the clinical trial protocol and the specific connotation of each indicator. Quality control personnel should check the basic conditions of clinical trials to ensure that the clinical trial conditions can meet the requirements of the protocol. During the trial, the investigator should earnestly perform clinical operations and other work in accordance with the SOP of the department and the requirements of the trial protocol, and record in an authentic, timely, complete and standardized manner. Quality control personnel shall perform quality verification on trial process and corresponding original records. At the end of the trial, the Study Site shall sort out corresponding project documents, which shall be archived and preserved after being checked by QC personnel. The Quality Assurance Department of the clinical study site audited the implementability of the trial conducted. In case of any non-conformance identified, timely notify the investigator and the responsible person of the unit to make correction, and track the correction.

- 1. Quality assurance of the data transfer, calculation, and reporting process

After the site data are entered into the eCRF, the QC personnel should check the consistency between the eCRF data and original records to ensure that the data are accurately entered into the eCRF. The monitor 100% checked whether the trial data entry in the eCRF system was complete and accurate and consistent with the original medical records. For the data items with questions or inconsistent with original data, timely raise a query and urge the data entry personnel and investigators to reply to the query and verify and correct the inconsistent data.

When the personnel of data management department use edit check, check the quality of data entry, send the questionable results to the investigator in the form of query, and the investigator will make modification after verification. QC personnel check data management documents and database data.

The quality assurance personnel of the study site conduct spot check on data transfer documents and data in statistical analysis report to ensure the accuracy of data.

The Sponsor shall audit the clinical trial process, sample test process, data, report and settlement process mentioned above from different aspects as needed in combination with the trial progress and the verification results of quality control personnel/monitor.

1. Data Management

The electronic Medical record report form (eCRF) was used to manage and record the study data through the electronic data capture system (EDC). EDC will automatically record the audit trace of all role operations such as data saving, modification, deletion, proofreading, audit, freezing, e-signature and lock.

A clinical data manager finalized the data management plan (DMP) before the first subject was enrolled. DMP specifies and records all tasks of clinical data management in detail and comprehensively, including personnel roles, work content, operation specifications, etc. The database designer is responsible for creating the database, designing eCRF and logical verification according to the trial scheme, CDASH and internal standards, and conducting user acceptance test (UAT) of the system after the configuration is completed, and then the system is deployed online after the test is passed. Normal range configuration was completed before site activation or data entry. Researchers are required to receive user training and project training before being authorized to access the formal environment.

Investigators or authorized investigators were responsible for data entry, corrections, and revisions. Source data validation (SDV) were performed by clinical monitors against source documents to assess completeness and consistency and to challenge findings of incomplete or inconsistent data. Clinical data managers reviewed the accuracy, completeness and specification of study data according to eCRF Completion guideline (CCG) and data validation plan (DVP), and questioned the unreasonable logic or improperly recorded data. Researchers should timely reply and deal with questions, and fill in the reasons for modification of data. Clinical monitors and clinical data managers should promptly close resolved queries.

The clinical data manager was responsible for the consistency check of the SAE in the clinical trial database and the pharmacovigilance database, checked the accuracy and completeness of the SAE information in the two databases, wrote the SAE consistency check report, and communicated with the investigators, pharmacovigilance department and CRA.

After data entry, traceability and review, the final confirmation and electronic signature of the study data were performed by the investigators. Data changes are generally not allowed after the electronic signature. If there is any data change after the signature, the electronic signature is invalid. Researchers are required to sign the Electronic Signature Statement before implementing the electronic signature, which declares that the electronic signature has the same legal effect as the written handwritten signature. Usually the electronic signature is done after all the data is frozen.

The names of adverse events and comorbidities were coded with the use of the MedDRA dictionary, version 25.0 or higher, and the names of drugs were coded with the use of the WHODrug Global 2022 and above dictionary. Medical codes were completed before the data were locked.

After data cleaning, the clinical data manager wrote a data review report, and at a data review meeting, the principal investigator, statistician, project manager, medical staff, and data management staff discussed protocol violations and identified the population for statistical analysis.

After the clinical data manager confirmed that the database lock task list was complete, the database lock was performed. After the database is locked, the principle is no longer unlocked. If it is necessary to unlock, the unlocking conditions and procedures must conform to the corresponding SOP, and the unlocking process must be carefully controlled and carefully recorded.

After the database was finally locked, the original database was exported, the SAS programmers made the submission data set, the analysis data set, the data description file and the annotation CRF, etc. The clinical data manager was responsible for archiving all data management related materials.

For specific task flow and quality assurance of clinical data management, see data management standard operating procedure and data management plan.

The specific transmission form, frequency and content of external data management are described in the External Data Transmission Protocol.

1. Statistical Analysis
   1. Analysis Datasets

- Analysis Set Based on Randomization

Included All randomized treated subjects with HR-positive, HER2-negative advanced breast cancer. Efficacy analyses will be performed as randomized, regardless of the actual treatment received.

This analysis set is the primary analysis set for analysis of demographic data and baseline characteristics and evaluation of different estimated objectives.

- The safety data set (SS)

All subjects who take the study drug at least once and have safety evaluation data after medication constitute the safety population of this study. The safety population will be used for the analysis of safety data. Treatment assignment was analyzed by actual assignment.

In this trial, demographic, baseline data and estimated target analysis are based on randomized analysis set, SS is used to analyze laboratory test data and adverse event.

- 1. Statistical analysis plan

13.2.1 General principles

For quantitative variables, summary statistics will include the number of subjects, mean, standard deviation (SD), median, minimum, and maximum. The decimal places for the minimum or maximum will be the same as that recorded in the database. The decimal places for mean and median will report one more decimal place than the original data values in the database, and standard deviation will report two more decimal places than the original data values. All decimal places shall not exceed 4 decimal places.

For categorical variables, frequency counts and percentage of patients will be presented. The percentage will be presented with two decimal places.

13.2.2 Test level

The primary endpoint is tested by difference test, and the test level is two-sided α=0.05, and the confidence interval is 95% confidence. The O'Brien-Fleming alpha-spending function is used for type I error correction for interim analisis. The test level for the secondary efficacy endpoitts and safety analyses is α=0.05 (two-sided). A two-sided P value of 0.05 or less is considered statistically significant. The confidence interval is 95%.

13.2.3 Hypothesis test

The primary endpoint is investigator-assessed PFS, and the log-rank test is used to compare the survival process. The test hypothesis is as follows:

H0: The survival function is the same for the experimental group and the control group, S1 (t) = S2 (t);

H1: The survival function is different for the experimental group and the control group, S1 (t) ≠ S2 (t).

Test level: two-sided α=0.05.

13.2.4 Stratification factors/covariates

Three randomization stratification factors are designed for this study. This stratification factor will be included in the statistical model for analysis.

1) visceral metastatic disease (yes vs. no)

2) menopausal status (pre-, peri- or postmenopausal)

3) sensitivity to prior endocrine therapy (yes vs. no).

- 1. Study Population

13.3.1 Sugjects distribution

Description of subject enrollment, dropouts, completion of expected course of treatment and premature discontinuation in each site: number of cases and percentage.

Sugjects distribution in each data set: summarize the subject distribution in each analysis, and the reasons for excluding.

Drop out and premature discontinuation sugjects shall be described one by one: medication and reasons for premature discontinuation.

13.3.2 Protocol deviations

Summarize and describe the sugjects with protocol deviations.

13.3.3 Baseline characteristics Analysis of the two groups

The analysis of baseline characteristics is based on the randomized analysis set, mainly using descriptive analysis, including basic data, history of previous anti-tumor treatment, infection screening, physical examination, ECOG PS, height, weight, vital signs, serum pregnancy test, tumor markers, 12-Lead ECG, blood routine, urine routine, stool routine (including occult blood), thyroid function, coagulation function, liver function, renal function, blood lipid, electrolytes, blood lipase, serum amylase, fasting blood glucose, echocardiography, etc. Continuous variables describe their mean, standard deviation, median, minimum and maximum; categorical variables describe their frequency, frequency or constituent ratio.

Baseline is defined as the last non-missing measurement prior to forst dosing.

- 1. Efficacy evaluation

13.4.1 Primary estimated objective

Primary estimated method

Kaplan-Meier method is used to estimate the median PFS and 95% Confidence Interval (CI) based on the efficacy data evaluated by the investigators, and Kaplan-Meier curve is drawn. The baseline risk is assumed to differ across strata. Based on this assumption, the stratified Log-rank results are used for comparison between groups, and the stratified Cox proportional hazards model is used to estimate the Hazard Ratio (HR) between groups. The stratification factors are shown in 14.2.4.

Sensitivity analysis 1

This sensitivity analysis is based on the assumption that the baseline risk is the same across strata. Investigator-assessed efficacy data are used for between-group comparisons with an unstratified Log-rank test, and an unstratified Cox proportional-hazards model is used to estimate HR between groups.

Sensitivity Analysis 2

Missing two or more consecutive efficacy assessments prior to an event will be censored. Analytical methods and other hypothetical homogenous estimation methods. This sensitivity analysis is designed to examine the robustness of the overall results under the assumption of no disease progression in the missing disease assessment interval.

Supplementary estimated objective 1

With investigator-assessed efficacy data, Cox proportional-hazards models with covariate adjustment are used to estimate HR between groups, with the above stratification factors included as covariates. This analysis corresponds to a different scientific question: whether treatment with TQB3616 capsules and fulvestrant injection, compared with placebo plus fulvestrant, would prolong PFS for patients with a covariate value of the population mean.

13.4.2 Secondary estimated objective 1

Primary estimated method

The IRC accessed median PFS and 95% CI are estimated by the Kaplan-Meier method, and Kaplan-Meier curves are plotted. The stratified Log-rank test is used to compare the results between groups, and the stratified Cox proportional hazards model is used to estimate the HR between groups.

13.4.3 Secondary estimated objective 2

Primary estimated method

The median OS and 95% CI are estimated by the Kaplan-Meier method, and Kaplan-Meier curves are plotted. The stratified Log-rank test is used to compare the results between groups, and the stratified Cox proportional hazards model is used to estimate the HR between groups.

1 3.4.4 Secondary estimated objective 3

Primary estimated method

The median DOR and 95% CI are estimated by the Kaplan-Meier method, and Kaplan-Meier curves are plotted. The stratified Log-rank test is used to compare the results between groups, and the stratified Cox proportional hazards model is used to estimate the HR between groups.

1 3.4.5 Secondary estimated objective 4

Primary estimated method

The ORR and 95% CI of the two groups are calculated. The 95%CI is calculated based on the exact binomial method of the F-distribution. The stratified Mantel-Haenszel test is used to compare ORR between groups. Logistic regression is used to estimate the Odds Ratio (OR) between the two groups.

1 3.4.6 Secondary Estimate Objective 5

Primary estimated method

The CBR and 95% CI of the two groups were calculated. The 95%CI was calculated based on the exact binomial method of the F-distribution. The stratified Mantel-Haenszel test was used to compare ORR between groups. Logistic regression was used to estimate the OR between the two groups.

- 1. Safety evaluation

13.5.1 Drug exposure

Drug exposure in each group is described by mean, standard deviation, maximum, minimum, and median.

Summarize the exposure of subjects to the study drug treatment, the number of cycles completed by patients in each group, the dose adjustment during treatment, and the cumulative number of dose adjustments during treatment.

The duration time of study drug, total dose of study drug and daily average dose during treatment period will be statistically described.

13.5.2 Adverse events

All AEs will be coded using the Medical Dictionary for Regulatory Affairs (MedDRA) for safety analysis.

Summary of the number of events, the number (incidence) and the percentage of subjects with the following: adverse events, treatment-emergent adverse events (TEAEs), treatment-emergent improtant adverse events, treatment-emergent adverse events of special interest, grade ≥3 TEAE, treatment-emergent serious adverse events (SAE), treatment-related TEAEs, treatment-emergent SAEs related to study drugs, TEAEs leading to dose adjustment, treatment termination, study termination, and death.

TEAEs or treatment-related TEAEs with ≥5% incidence will also be summarized by PT.

TEAEs or treatment-related TEAEs with ≥10% incidence will also be summarized by PT.

A frequency table of AEs will be provided for each intervention arm by SOC and PT. The incidence will be calculated by the system, symptoms, and vital signs (the number of events is defined as the number of patients with at least one event).

The number of events, the number (incidence) and the percentage of patients with TEAEs will also be provided by severity grade per NCI-CTCAE v5.0 and the causality to the study drugs. A patient will be counted only once by the worst severity grade per NCI-CTCAE v5.0.

Listings of patients with TEAEs and SAEs will be provided.

13.5.3 Vital Signs

The values and changes in vital signs before and after the treatment will be summarized by mean, SD, minimum, maximum, and median in each intervention arm.

13.5.4 Laboratory test indicators

Hematology, urinalysis, thyroid function, coagulation function, liver function, renal function, four items of blood lipid, electrolyte, blood lipase and serum amylase were used to describe measured values and change values before and after treatment in each group using mean ± standard deviation, maximum, minimum and median. Cross-classification tables were used to describe normal and abnormal changes before and after treatment.

The proportion of subjects with "clinically significant abnormalities" abnormal changes will be described, and whether the abnormality is clinically significant will be judged by the investigator.

13.5.5 Electrocardiogram

Electrocardiogram: normal and abnormal according to the investigator's judgment. Normal and abnormal changes before and after treatment were described.

Heart rate, PR interval, QRS interval, QT interval and QTc were measured by mean ± standard deviation, maximum, minimum and median to describe the measured values and changes in the two groups before and after treatment. The overall electrocardiogram assessment results were used to describe the normal and abnormal changes before and after medication using the cross-classification table. The proportion of subjects with abnormal changes who were described as having "clinically significant abnormalities," where the clinical significance of the abnormality was judged by the investigator. The list presents a list of post-dose abnormalities.

13.5.6 Concomitant medication

The use of concomitant medications during the study period (including any changes in concomitant medications during screening, new concomitant medications after screening, and concomitant medications during follow-up) and the frequency of use of individual medications will be summarized.

The Anatomical Therapeutic Chemical (ATC) codes in WHODrug Global will be used for coding concomitant medications. The frequency of concomitant medications will be listed by main anatomical groups and therapeutic subgroups in the SS population.

- 1. Missing Data

In sensitivity analyses of the primary estimated objective 2, two or more consecutive missing efficacy assessments before the occurrence of an event a re censored. At the same time, the loss of subjects to follow-up can also lead to missing data. Participants are censored if they aree lost to follow-up for the analyses of the primary estimated objective and secondary estimated objectives 1, 2, and 3. Based on the assumption of missing at random (MAR), the likelihood-based method, Cox proportional hazards model, is used to deal with missing data.

In the analyses of secondary estimated objectives 4 and 5, treatment effects that are missing are treated as “nonresponse”.

Detailed rules for handling missing data are described in the Statistical Analysis Plan (SAP).

- 1. Subgroup analysis

Subgroup analysis are performed in the randomized analysis set only for the primary endpoint, investigator-assessed PFS. Subgroup factors included but were not limited to the following:

- Premenopausal or perimenopausal vs. postmenopausal
- PR positive vs. PR negative
- Metastatic site (visceral metastasis vs. bone metastasis vs. other)
- Measurable disease (yes vs. no)
- Number of metastatic organs (< 3 vs. ≥ 3)
- Prior Endocrine Therapy (SERMs vs. AI vs. SERMs and AI)
- Lesion age (< 65 vs. ≥ 65)
- ECOG PS (0 vs. 1)
- Prior Rescue Chemotherapy (Yes vs. No)
- Sensitivity to prior endocrine therapy (Yes vs No)
- Disease stage (locally advanced vs. metastatic disease)

In addition, subgroup analysis based on other potentially clinically significant factors will be performed in the statistical analysis plan (SAP).

- 1. Statistical analysis software

Statistical analysis is performed using SAS9.4 software.

1. Ethics and Informed Consent

This clinical trial must be conducted in accordance with the Declaration of Helsinki and the relevant guidelines and regulations for clinical trial research in China. The trial protocol was formulated before the start of the clinical trial, which was agreed and signed by the investigators and the sponsor, and submitted to the ethics committee for approval. If the protocol needs to be revised during the actual implementation of the clinical trial, the revised trial protocol will be submitted to the ethics committee for approval before implementation. If important new information related to the trial drug is found, the informed consent form must be revised in writing and submitted to the ethics committee for approval, and the consent of the subjects should be obtained again.

Prior to the initiation of a clinical trial, the investigator must provide the subject with detailed information about the clinical trial, including the nature of the trial, the purpose of the trial, the possible benefits and risks, alternative treatment options, and the rights and obligations of the subject. All participants provided informed consent and signed an ICF before starting the clinical trial.

1. Publication of trial results

The results are owned by Chia Tai Tianqing Pharmaceutical Group Co., LTD. Researchers (research institutions) should obtain the consent of Chia Tai Tianqing Pharmaceutical Group Co., LTD before publishing the paper. Prior to the official publication of the main study results, investigators (research units) should obtain the prior consent of Chia Tai Tianqing Pharmaceutical Group Co., Ltd. when communicating the results of the clinical trial at academic conferences.

1. References

1. 2014 China Subregional Analysis of Malignant Tumor Incidence and Death; Chinese Journal of Oncology; 2018, Vol. 27, No. 1.

2. Zhang Li, Wang Yong, Guo Yu; Progress in the treatment of hormone receptor-positive advanced breast cancer resistant to endocrine therapy; Tumor TUMOR Vol. 38, January 2018.

3. Xie Shao, Ding Jian, Chen Yi. Progress in the research and development of CDK inhibitors in the field of anti-tumor [J]. Progress in Pharmacy, 2015, 39 (10): 734-745.

4. Yang Fang, Guan Xiaoxiang. Progress of CDK4/6 Inhibitors in Breast Cancer Treatment [J]. Journal of Clinical Oncology, 2015, 20:654-657.
