# Supplementary material for: Novel CDK2/4/6 inhibitor culmerciclib (TQB3616) plus fulvestrant in previously treated, HR-positive, HER2-negative advanced breast cancer: a randomized, double-blind, phase 3 trial
Source: Signal Transduct Target Ther. 2025 Dec 18;10:414. doi: 10.1038/s41392-025-02475-6 (PMC12714862; doi:10.1038/s41392-025-02475-6)
Supplement: Supplementary file 3 — SAP [file 41392_2025_2475_MOESM3_ESM.docx]

Statistical Analysis Plan

| Protocol Name: | TQB3616 in Combination with Fulvestrant Versus Placebo in Combination with Fulvestrant in Previously Treated HR-Positive, HER2-Negative Advanced Breast Cancer: A Randomized, Double-Blind, Parallel-Controlled Phase III Trial |
| --- | --- |
| Protocol Number: | TQB3616-III-01 |
| Sponsor: | Chia Tai Tianqing Pharmaceutical Group Co., Ltd. |
| Statistical Analysis Department: | Department of Biostatistics, School of Public Health, Nanjing Medical University |
| SAP Version: | V1.0 |
| SAP Date: | May 9, 2024 |

Confidentiality Statement

This document contains proprietary information related to business secrets, owned by Chia Tai Tianqing Pharmaceutical Group Co., Ltd. Unauthorized dissemination, copying, or publication of this information is strictly prohibited.

Statistical Analysis Plan - Signature Page

I have thoroughly reviewed the " TQB3616 in Combination with Fulvestrant Versus Placebo in Combination with Fulvestrant in Previously Treated HR-Positive, HER2-Negative Advanced Breast Cancer: A Randomized, Double-Blind, Parallel-Controlled Phase III Trial (Protocol Number: TQB3616-III-01; Version: V4.0 ; Version Date: June 10, 2022 ) " and agree to conduct the statistical analysis of the trial results in accordance with this plan.

Statistical Analysis Department: Department of Biostatistics, School of Public Health, Nanjing Medical University

| Prepared by: |  |
| --- | --- |
| Sign Word: |  |
| Day Phase: |  |

| Reviewed by: |  |
| --- | --- |
| Sign Word: |  |
| Day Phase: |  |

Sponsor: Chia Tai Tianqing Pharmaceutical Group Co., Ltd.

| Medical Review: |  |
| --- | --- |
| Sign Word: |  |
| Day Phase: |  |
| Approved by: |  |
| Sign Word: |  |
| Day Phase: |  |

Version History

The Statistical Analysis Plan (SAP) will be updated, revised, or supplemented as needed, with version changes recorded in the table below. The latest version will be implemented.

| Version No. | Version Date | Prepared by | Remarks |
| --- | --- | --- | --- |
| V1.0 | 2024-05-09 | Loudonghua | Finalized before database lock |
|  |  |  |  |

CONTENTS

[Abbreviation 6](#_Toc207268753)

[1.Introduction 7](#_Toc207268754)

[2.Study overview 7](#_Toc207268755)

[2.1 Study objectives 7](#_Toc207268756)

[2.2 Overall trial design 8](#_Toc207268757)

[2.3 Randomization and blind method 9](#_Toc207268758)

[2 .3.1 Randomization 9](#_Toc207268759)

[2 .3.2 Blind method 10](#_Toc207268760)

[2.4 Sample size 11](#_Toc207268761)

[3. Study endpoints 11](#_Toc207268762)

[3.1 Primary Endpoint 11](#_Toc207268763)

[3.2 Secondary Endpoint 11](#_Toc207268764)

[4. Estimated objectives 12](#_Toc207268765)

[4.1 Primary Estimated Objective 12](#_Toc207268766)

[5. Statistical analysis datasets 13](#_Toc207268767)

[5.1 Analysis Set Based on Randomization 13](#_Toc207268768)

[5.2 Safety Analysis Set 14](#_Toc207268769)

[6.Special data handling 14](#_Toc207268770)

[6.1 Handling of Outliers 14](#_Toc207268771)

[6.2 Handling of Missing Data 14](#_Toc207268772)

[6.3 Other Data Handling 15](#_Toc207268773)

[6.4 Baseline Definition 16](#_Toc207268774)

[6.5 Time Calculation 17](#_Toc207268775)

[7. Statistical analysis 18](#_Toc207268776)

[7.1 General Principles 18](#_Toc207268777)

[7.1.1 Hypothesis test 19](#_Toc207268778)

[7.1.2 Interim Analyses 19](#_Toc207268779)

[7.1.3 Significance level 20](#_Toc207268780)

[7.1.4 Stratification factors 20](#_Toc207268781)

[7.2 Study Population 21](#_Toc207268782)

[7.2.1 Subject Disposition 21](#_Toc207268783)

[7.2.2 Protocol Deviations 21](#_Toc207268784)

[7.2.3 Demographic data and baseline characteristics 21](#_Toc207268785)

[7.3 Efficacy Analysis 23](#_Toc207268786)

[7.3.1 Analysis of primary estimand/primary endpoint 23](#_Toc207268787)

[7.3.2 Analysis of Secondary Estimate Objectives/Secondary Endpoints 25](#_Toc207268788)

[7.4 Safety analysis 26](#_Toc207268789)

[7.4.1 Drug Exposure Analysis 26](#_Toc207268790)

[7.4.2 Adverse events 28](#_Toc207268791)

[7.4.3 Laboratory tests 31](#_Toc207268792)

[7.4.4 Electrocardiogram 33](#_Toc207268793)

[7.4.5 Vital Signs 34](#_Toc207268794)

[7.4.6 ECOG Score 34](#_Toc207268795)

[7.4.7 Concomitant medication 34](#_Toc207268796)

[7.5 Subgroup Analysis 35](#_Toc207268797)

[8. Description of this plan 36](#_Toc207268798)

[Attachments 1: Protocol deviation definition 38](#_Toc207268799)

[Attachments 2: potentially clinically important criteria for laboratory parameters 39](#_Toc207268800)

# Abbreviation

| AE | adverse event | NMPA | National Medical Products Administration |
| --- | --- | --- | --- |
| AESI | Adverse Events of Special Interest | ORR | objective response rate |
| CR | complete response | OS | overall survival |
| CRF | case report form | PD | progressive disease |
| CTCAE | Common Terminology Criteria for Adverse Events | PFS | Progression-Free-Survival |
| DCR | disease control rate | PPS | Per-Protocol Set |
| IDMC | Independent Data Monitoring Committee | PR | partial response |
| DOR | Duration of Response | RECIST | response evaluation criteria in solid tumors |
| ECOG | Eastern Cooperative Oncology Group | SAE | serious adverse event |
| HR | Hazard Ratio | SD | stable disease |
| ITT | intend to treat | SS | Safety Analysis set |
| IRC | Independent Review Committee | SAP | Statistical Analysis Plan |
| IMDC | International Metastatic Renal Cell Carcinoma Database Consortium | TEAE | Treatment Emergent Adverse Events |

# 1.Introduction

The Statistical Analysis Plan (SAP) is a standalone document that provides more technical and operational details than the analysis points described in the trial protocol. It includes detailed procedures for the statistical analysis of primary and secondary endpoints, as well as other data. The SAP should be drafted by statistical professionals with clinical trial experience and should comprehensively and thoroughly describe the methods and presentation of statistical analyses, as well as the interpretation of expected statistical results.

The initial draft of the SAP should be prepared after the trial protocol and case report forms (CRFs) are finalized. The official document should be completed and signed before database lock.

This SAP provides the statistical analysis methods and data processing principles for the TQB3616 clinical study, including definitions of analysis populations, variables to be evaluated, and descriptions of statistical methods. All statistical analyses will be performed using SAS® Version 9.4 or higher. Any inconsistencies between the actual analysis and the SAP will be explained in the **Clinical Study Report**.

This SAP is based on the protocol for the TQB3616 in Combination with Fulvestrant Versus Placebo in Combination with Fulvestrant in Previously Treated HR-Positive, HER2-Negative Advanced Breast Cancer: A Randomized, Double-Blind, Parallel-Controlled Phase III Trial (Protocol Number: TQB3616-III-01; Version: V4.0 ; Version Date: June 10, 2022 ).

# 2.Study overview

## 2.1 Study objectives

- Primary objective:

To evaluate the progression free survival (PFS) of TQB3616 plus fulvestrant versus placebo plus fulvestrant in subjects with previously treated HR-positive, HER2-negative advanced breast cancer.

- Secondary objectives:

To evaluate the overall survival (OS), objective response rate (ORR), clinical benefit rate (CBR), duration of response (DOR) of TQB3616 plus fulvestrant versus placebo plus fulvestrant in subjects with previously treated HR-positive, HER2-negative advanced breast cancer.

To evaluate the safety of TQB3616 in combination with fulvestrant versus placebo in combination with fulvestrant in subjects with previously treated HR-positive, HER2-negative advanced breast cancer, including incidence and severity of adverse events (AEs), serious adverse events (SAEs) and abnormal laboratory values.

## 2.2 Overall trial design

This is a randomized, double-blind, parallel-controlled, multicenter study. Patients were randomized in a 2:1 ratio to receive TQB3616 plus fulvestrant (experimental group) or placebo plus fulvestrant (control group). Randomization is stratified according to:

1) visceral metastatic disease (yes vs. no)

2) menopausal status (pre-, peri- or postmenopausal)

3) sensitivity to prior endocrine therapy (yes vs. no).

Efficacy assessments are performed every 8 weeks (56 days) and for every 12 weeks (84 days) after 48 weeks. For patients with disease control (CR + PR + SD) and tolerable adverse reactions, the treatment can be continued until loss of clinical benefit, intolerable toxicity and the investigator consider it inappropriate to continue the treatment.

## 2.3 Randomization and blind method

### 2 .3.1 Randomization

This study is a multicenter, randomized, double-blind, parallel controlled trial. The randomization method is central stratified randomization, each center competed for enrollment, and the random ratio is 2:1. Subjects are randomized using a central randomization system. After the official launch of the project, the blind results are uploaded by independent statisticians. For the subjects who had signed the informed consent form and were successfully screened, the investigators are required to log in the central randomization system and input the basic information of the subjects, including the research center, the abbreviation of the subject's name, gender, date of birth, stratification factors, etc. After confirming the accuracy of the basic information and stratification factors of the subjects, the investigators or their authorized personnel underwent randomization. The central randomization system will return the randomization results for that subject, including: the randomization number and the corresponding drug number. The drug manager of the sub-center will issue the corresponding drug according to the drug number. The random number of each successfully randomized subject is uniquely and permanently identified. Patients who underwent randomization but did not receive a trial drug had their drug and drug number invalided and could not be reassigned. The monitor could log into the system to verify the basic information of the subjects and the drug use of each center, and send questions to the responsible person for the information in doubt.

Randomization is stratified according to the following three:

1) visceral metastatic disease (yes vs. no)

2) menopausal status (pre-, peri- or postmenopausal)

3) sensitivity to prior endocrine therapy (yes vs. no).

Note: Prior endocrine therapy-sensitive, defined as: disease progression after 2 years of adjuvant endocrine therapy, or disease progression after 6 months of first-line endocrine therapy. Visceral metastasis is defined as: systemic organs in the chest, abdomen and pelvis, excluding skull and musculoskeletal region.

### 2 .3.2 Blind method

This study uses a double-blind design, and the investigators, researchers involved in the evaluation of trial effects, data managers, statistical analysts, and subjects and their relatives or guardians are blinded to the treatment assignments. Blinded bases are generated with the use of SAS software by an independent statistician who is not associated with the final trial statistics and are uploaded to the central randomization system after the official start of the study. Blind bases are kept in a central randomization system during the study.

Emergency unblinding

- Conditions for emergency unblinding

In case of emergency (such as serious adverse events, serious complications, etc.), the subjects need rescue, and the rescue measures depend on knowing what treatment the patient receives, the blind can be unblinded urgently.

- Procedures for emergency unblinding

Online emergency unblinding is used. If necessary, the sub-site principal investigator may request online emergency unblinding for a specific subject through the central randomization system. Only the sub-site principal investigators have access to emergency unblinding.

- Independent emergency unblinding of pharmacovigilance

When SUSAR occurs, the independent pharmacovigilance specialist applies for the blind base to the system leader, who sends it directly to the pharmacovigilance specialist after the review by the system leader, who is responsible for keeping and maintaining the blind base.

- Records of unblinding

After the emergency unblinding, subsequent situations should be recorded in the corresponding original medical records/electronic case report form. The investigator should fill in the record form for emergency unblinding, and send the scanned copy to the sponsor for preservation.

- Treatment after unblinding

Once a subject is unblinded, the subject with this number will withdraw from the trial and will not be replaced. The unblinding subject should be followed until improvement or stable state is achieved.

## 2.4 Sample size

This study uses a randomized, double-blind, parallel-controlled, multicenter trial designand PFS is the primary endpoint.

The number of patients required for this trial was based on the primary end point of investigator-assessed PFS and was calculated with the use of a predefined stratified log-rank test. Assuming a median PFS of 6.0 months for placebo plus fulvestrant, we estimated that 186 events of progression or death would be required in the two treatment groups for the study to have 90% power to detect a hazard ratio (HR) of 0.60 with a two-sided significance level of α = 0.05. A total sample of 243 patients was required. Assuming an attrition rate of 15%, at least 287 patients (191 for the experimental group and 96 for the control group) were anticipated. The primary end point was to be analyzed at the interim analysis at approximately 70% maturity in the overall population when 131 events of progression or death had occurred and at the final analysis at 100% maturity when 186 events of progression or death had occurred. Type I errors were controlled using the Lan-DeMets spending function approximating O’Brien-Fleming boundary.

# 3. Study endpoints

## 3.1 Primary Endpoint

Progression-free survival (PFS) assessed by the investigator.

## 3.2 Secondary Endpoint

- Effectiveness indicators
- Progression-free survival (PFS) assessed by independent imaging assessment;
- Overall survival (OS);
- Objective response rate (ORR);
- Clinical benefit rate (CBR);
- Duration of response (DOR);
- Safety indicators
- Incidence and severity of adverse events (AEs), laboratory abnormalities, and serious adverse events (SAEs).

# 4. Estimated objectives

The main clinical concerns of this study are: in subjects with HR-positive, HER2-negative advanced breast cancer defined by the inclusion and exclusion criteria, the Hazard Ratio (HR) is used as the effect size at the population level to study the efficacy of drugs through investigator-assessed PFS, regardless of early termination of treatment for any reason (an endpoint event is not reached) and under the assumption of no new antitumor therapy.

## 4.1 Primary Estimated Objective

Given that in our trial, early treatment termination reflects clinical practice, we considere the treatment strategy as the concomitant event of primary estimated objective: Strategies for early treatment termination. A hypothetical strategy is used to manage concomitant events: starting a new antitumor therapy before the PFS event. The main estimated primary objectives are defined as follows:

Target population: HR+/HER2- advanced breast cancer patients defined by inclusion and exclusion criteria;

Treatment: Subject Received TQB3616 Capsules/Placebo Capsules, Fulvestrant Injection (Qingkeyi);

Target Variables: PFS assessed by the investigator, defined as from randomization to PD or death, whichever occurs first;

Concomitant event and treatment strategy:

| Concomitant event | Treatment strategy | Notes |
| --- | --- | --- |
| Initiation of new antineoplastic therapy prior to PFS event | Imaginary strategy | If no new anticancer therapy occurs, the new anticancer therapy time point will be censored |
| Early termination of treatment | Therapeutic strategy | Ignored concomitant events and PFS time is used regardless of early discontinuation of treatment |

Population summary: Hazard ratio (HR).

Note: Early termination of treatment mainly includes the following:

1. The subject still cannot tolerate the toxicity after dose adjustment;
2. The subject requires premature termination of the investigational drug and voluntary withdrawal;
3. Other reasons that the investigator believes the subject is unable to continue the study treatment (lack of clinical benefit, worsening health condition, etc.);
4. The subject experiences a pregnancy event during the study;
5. Subjects who, in the opinion of the investigator, are noncompliant with study procedures or study drug administration, require early termination of treatment.

# 5. Statistical analysis datasets

## 5.1 Analysis Set Based on Randomization

Included All randomized treated subjects with HR-positive, HER2-negative advanced breast cancer. Efficacy analyses will be performed as randomized, regardless of the actual treatment received.

This analysis set is the primary analysis set for analysis of demographic data and baseline characteristics and evaluation of different estimated objectives;

## 5.2 Safety Analysis Set

All subjects who take the study drug at least once and have safety evaluation data after medication constitute the safety population of this study. The safety population will be used for the analysis of safety data. Treatment assignment was analyzed by actual assignment.

In this trial, demographic, baseline data and estimated target analysis are based on randomized analysis set, SS is used to analyze laboratory test data and adverse event.

# 6.Special data handling

## 6.1 Handling of Outliers

In a set of parallel measurements, individual values that are significantly different from others are called outliers or extreme values. Outliers will not be handled in this study.

## 6.2 Handling of Missing Data

If a subject has missing or unevaluable tumor assessments for all visits (based on RECIST 1.1 criteria), they will be classified as ‘non-responder’ and included in the denominator but not the numerator for ORR and CBR calculations.

Missing data for OS, PFS, and DOR will be treated as censored data. The censoring rules are shown in the table below.

Table 1: Censoring Rules for Survival Analysis (Efficacy Endpoints)

| **Data Scenario** | **Calculation Date** | **Censored** |
| --- | --- | --- |
| **Progression-Free Survival (PFS), Duration of Response (DOR)** |  |  |
| Disease progression confirmed by imaging | Imaging date | No |
| Death without imaging-confirmed progression | Death date | No |
| Disease progression after a missed imaging assessment (before the next planned imaging) | Imaging date | No |
| Death after a missed imaging assessment (before the next planned imaging) | Death date | No |
| No baseline imaging assessment | Randomization date | Yes |
| No disease progression or death during the trial | Last imaging date | Yes |
| No post-baseline imaging assessment (no death before the first planned imaging) | Randomization date | Yes |
| Treatment discontinuation due to unrecorded progression | Last imaging date | Yes |
| New anti-cancer therapy initiated before progression | Last imaging date before new therapy | Yes |
| Treatment discontinuation due to toxicity or other reasons | Last imaging date | Yes |
| Disease progression or death after two or more consecutive missed imaging assessments | Last imaging date before missing data | Yes |
| **Overall Survival (OS)** |  |  |
| Death | Death date | No |
| Completion of the trial without death | Last known alive date | Yes |
| Early withdrawal from the trial without death | Last known alive date | Yes |
| No follow-up information after the first dose | Randomization date | Yes |

For PFS, OS, and DOR calculations, if the event date is missing, the following rules will be applied:

- If only the day is missing, it will be replaced with the 1st of the month;
- If both the day and month are missing, it will be replaced with January 1st;
- If the year, month, and day are all missing, no imputation will be performed.

Note: The imputed progression date should not be earlier than the most recent imaging date, and the imputed death date should not be earlier than the last known alive date.

For safety evaluations, missing data will not be imputed.

## 6.3 Other Data Handling

Unless otherwise specified, when dates are missing, the following rules will be applied for imputation:

- If the start day is missing, it will be replaced with the 1st of the month;
- If both the start day and month are missing, it will be replaced with January 1st;
- If the end day is missing, it will be replaced with the last day of the month;
- If both the end day and month are missing, it will be replaced with December 31st;
- If the year, month, and day are all missing, no imputation will be performed, and the date will remain missing.

For adverse events and concomitant medications, if the start or end dates are partially missing, the following rules will be applied:

- If the start day is missing and the year and month match the first dose date, the start date will be imputed as the first dose date; otherwise, it will be replaced with the 1st of the month;
- If both the start day and month are missing and the year matches the first dose date, the start date will be imputed as the first dose date; otherwise, it will be replaced with January 1st;
- If the end day is missing, it will be replaced with the last day of the month;
- If both the end day and month are missing, it will be replaced with December 31st;
- If the start year, month, and day are all missing, the start date will be set as the first dose date;
- If the imputed start date is after the end date, the start date will be set equal to the end date.

Dates in data listings will be presented as recorded in the CRF.

## 6.4 Baseline Definition

Unless otherwise specified, baseline is defined as the last non-missing measurement before the first dose of the study drug.

Changes from baseline are defined as: post-treatment measurement – baseline measurement.

## 6.5 Time Calculation

Time (in days) related to efficacy endpoints: (post-treatment date – randomization date) + 1, where the post-treatment date is selected according to the definition of the efficacy endpoint. Each month is calculated as 30.4375 days.

Disease duration (in days) = (randomization date – initial diagnosis date) + 1, with each month calculated as 30.4375 days. If the diagnosis date is missing, the following rules will be applied:

- If the day is missing, it will be replaced with the 15th of the month;
- If both the day and month are missing, it will be replaced with July 15th;
- If the year, month, and day are all missing, no imputation will be performed, and the date will remain missing.

PFS Conventions for Time Calculation:

- For subjects with endpoint events, PFS time is calculated as:

1. Imaging-confirmed progression date (earliest date among target lesions, non-target lesions, and new lesions) – randomization date + 1;
2. Death date – randomization date + 1.

If both 1) and 2) apply, the earliest date will be used.

- For subjects without endpoint events, PFS time is calculated as:

1. Last imaging date – randomization date + 1 (treated as censored data);
2. if no imaging was performed：1 day (treated as censored data).

If a subject withdraws without any imaging after randomization, PFS will be censored at 1 day.

DOR Time Calculation Rules:

- DOR censoring rules are the same as for PFS. DOR will only be analyzed for subjects who achieve CR or PR. For subjects with endpoint events, DOR time is calculated as:

1. Imaging-confirmed progression date (earliest date among target lesions, non-target lesions, and new lesions) – first CR or PR date + 1;
2. Death date – first CR or PR date + 1.

If both 1) and 2) apply, the earliest date will be used.

- For subjects without endpoint events, DOR time is calculated as:

1. Last imaging date – first CR or PR date + 1 (treated as censored data);
2. if no imaging was performed：1 day (treated as censored data)

OS Time Calculation Rules:

- For deceased subjects: (death date – randomization date) + 1;
- For surviving subjects: (last known alive date – randomization date) + 1 (treated as censored data).
- For subjects with no follow-up information after the first dose: 1 day (treated as censored data).

# 7. Statistical analysis

## 7.1 General Principles

Although this analysis plan includes all pre-defined analyses, additional exploratory analyses may be identified during the study. These will be described in detail in the clinical study report.

Statistical analyses will be performed using SAS® Version 9.4 (or higher). For continuous variables, descriptive statistics will include the number of subjects, mean, standard deviation, median, quartiles (Q1, Q3), minimum, and maximum. For categorical variables, descriptive statistics will include frequencies and/or percentages.

The table below shows the number of decimal places to be retained for statistical parameters:

Decimal Places for Statistical Parameters

| **Statistic** | **Decimal Places** |
| --- | --- |
| Mean, Median | One more decimal place than the raw data, up to 4 decimal places |
| Standard Deviation | Two more decimal places than the raw data, up to 4 decimal places |
| Maximum, Minimum | Same as the raw data, up to 4 decimal places |
| Percentage | 2 decimal places |
| Statistics, Confidence Intervals | 2 decimal places |
| P-value | 4 decimal places, or P<.0001 if the first 4 decimal places are zero |

### 7.1.1 Hypothesis test

The primary efficacy endpoints of this trial was Investigator-assessed Progression-free survival (PFS), and the log-rank test is used to compare the survival. The test hypothesis is as follows:

H0: The PFS distribution of the experimental and control groups is the same, i.e. S1 (t) = S2 (t);

H1: The PFS distribution of the experimental and control groups is different, i.e. S1 (t) ≠ S2 (t).

### 7.1.2 Interim Analyses

This study established an Independent Data Monitoring Committee (IDMC) to conduct an interim analysis. The IDMC comprises two independent oncology experts and one independent statistician, operating independently from the sponsor and investigators.

The interim analysis aims to evaluate the study’s efficacy based on the primary endpoint of PFS (Progression-Free Survival). When 70% of PFS events (131 cases) are observed, the efficacy analysis will be performed. If efficacy meets expectations, a premature regulatory filing may be initiated. The control of overall type I error rate will utilize the Lan-DeMets alpha spending function (O’Brien-Fleming type) (detailed specifications are provided in Section 6.1.3). The decision rules for this interim analysis are specified in the IDMC charter.

### 7.1.3 Significance level

This study will conduct an efficacy-focused interim analysis of PFS when 70% of PFS events (131 cases) are reached. If the efficacy meets predefined criteria, early regulatory submission will be initiated. The IDMC is responsible for this interim analysis.

The O’Brien-Fleming alpha spending function will be employed to ensure control of the overall type I error rate at 0.05 (two-sided). According to this method: The interim analysis significance level is set at two-sided α1=0.01477 and the final analysis significance level is two-sided α2=0.04551. Should the actual event proportion exceed 70% during interim analysis, the analysis will utilize O’Brien-Fleming type boundaries derived from the Lan-DeMets alpha spending function based on the actual number of events observed at the time of the interim analysis (see IDMC charter for details).

For secondary efficacy endpoints and safety analyses, the significance level is α=0.05 (two-sided). Results with P≤0.05 will be considered statistically significant. All confidence intervals will be given at a two-sided 95% level.

### 7.1.4 Stratification factors

This study was designed with three randomization stratification factors and these stratification factors will be included in the statistical model for analysis.

1) visceral metastatic disease (yes vs. no)

2) menopausal status (pre-, peri- or postmenopausal)

3) sensitivity to prior endocrine therapy (yes vs. no).

The principle of intention-to-treat (ITT) was followed. The stratification factors and covariates for efficacy analysis were based on the stratification factors at randomization; the actual stratification factors were used for analysis.

## 7.2 Study Population

### 7.2.1 Subject Disposition

The number and percentage of subjects screened, randomized, randomized and treated, completing the planned treatment course, and discontinuing treatment early will be summarized by center. Subjects who fail screening will be summarized by center and primary reason. Subjects who discontinue treatment after randomization will be summarized by center, treatment group, and primary reason.

The distribution of subjects across analysis sets will be summarized, including reasons for exclusion from each analysis set.

Subjects excluded from the analysis sets will be listed by center and treatment group, including protocol deviations and reasons for exclusion.

Subjects who discontinue treatment after randomization will be listed by center and treatment group.

A list of screening failure reasons will be provided. A subject distribution chart will also be provided.

### 7.2.2 Protocol Deviations

For the randomized analysis set, summarize by treatment group the number and percentage of subjects with no major protocol deviations and with at least one major protocol deviation; summarize the number and percentage of each category of major protocol deviations; list all major protocol deviations.

### 7.2.3 Demographic data and baseline characteristics

All demographic and baseline characteristics will be analyzed and listed based on the FAS. Continuous variables will be described using the number of subjects, mean, standard deviation, median, quartiles, minimum, and maximum. Categorical variables will be described using frequencies, percentages, or proportions.

Demographic data mainly included: age, age groups (i.e. < 65, ≥ 65 ), ethnicity, menopausal status, height, weight, and BMI.

Baseline characteristics mainly include: vital signs (respiration, blood pressure, body temperature, pulse), baseline disease characteristics (disease duration, ECOG Score, Presence/absence Viscera Transfer , Presence/absence Liver Transfer , Presence/absence Lung Transfer , Presence/absence Bone Transfer , no visceral metastasis (bone metastasis only, other), number of organs involved by metastasis, measurable lesion at baseline, ER Judgment results, PR Judgment results, HER2 Gene amplification assays), Past medical history ( Oncology Treatment History (whether surgery, radiotherapy, drug therapy), whether previous endocrine therapy is sensitive, whether rescue chemotherapy exists, Allergy history , past medical history), type of previous endocrine therapy, previous endocrine therapy drugs, laboratory tests (blood routine, blood biochemistry, etc.), 12- Epilepsy Electrocardiogram.

- Medical history and concomitant diseases will be classified using the latest version of the Medical Dictionary for Regulatory Activities (MedDRA) at the start of coding. They will be summarized by system organ class (SOC) and preferred term (PT), with the number and incidence of events reported for each treatment group. Medical history refers to events that occurred before randomization and did not persist, while concomitant diseases refer to events that occurred before randomization and persisted or ended after randomization. If the start or end date is missing or partially missing, the event will be considered a concomitant disease unless there is evidence that it ended before randomization.
- Prior medications will be classified using the WHODrug Global Anatomical Therapeutic Chemical (ATC) classification system. The number and incidence of prior medications will be summarized by anatomical main group and active substance. Prior medications are defined as concomitant medications that ended before the first dose of the study drug.
- Baseline imaging of target lesions will be summarized by treatment group, including the number of target lesions, sum of target lesion diameters, and number of target lesion organs. Non-target lesions will be summarized by treatment group, including the number of subjects with at least one non-target lesion and the number of non-target lesions.
- Laboratory tests and ECG will focus on key baseline values, such as those identified as important prognostic or treatment response indicators, or safety-related indicators.

## 7.3 Efficacy Analysis

Analyses of efficacy measures were based on the randomized analysis set.

### 7.3.1 Analysis of primary estimand/primary endpoint

- Progression-free survival assessed by the investigator (PFS )

Primary Analysis Method

For the randomized analysis set, Kaplan-Meier method is used to estimate the median PFS and 95% Confidence Interval (CI) based on the efficacy data evaluated by the investigators, and Kaplan-Meier curve is drawn. The baseline risk is assumed to differ across strata. Based on this assumption, the stratified Log-rank results are used for comparison between groups, and the stratified Cox proportional hazards model is used to estimate the Hazard Ratio (HR) between groups. The stratification factors are shown in 6.1.4. Also calculate 6-month, 12-month, and 18-month PFS rates.

Sensitivity Analysis 1

This sensitivity analysis is based on the assumption that the baseline risk is the same across strata. Investigator-assessed efficacy data are used for between-group comparisons with an unstratified Log-rank test, and an unstratified Cox proportional-hazards model is used to estimate HR between groups.

Sensitivity Analysis 2

Missing two or more consecutive efficacy assessments prior to an event will be censored. Analytical methods and other hypothetical homogenous estimation methods. This sensitivity analysis is designed to examine the robustness of the overall results under the assumption of no disease progression in the missing disease assessment interval.

Supplementary estimated objective 1

With investigator-assessed efficacy data, Cox proportional-hazards models with covariate adjustment are used to estimate HR between groups, with the above stratification factors included as covariates. This analysis corresponds to a different scientific question: whether treatment with TQB3616 capsules and fulvestrant injection, compared with placebo plus fulvestrant, would prolong PFS for patients with a covariate value of the population mean.

Supplementary estimated objective 2

The concomitant event ‘COVID-19 infection (or COVID-19 pandemic) causing treatment interruption >7 days’ will be considered and handled using hypothetical strategies. For investigator-assessed PFS, subjects with treatment interruption >7 days due to COVID-19 infection (or pandemic) will be censored. This analysis aims to evaluate the impact of COVID-19 infection (or pandemic) on PFS.

Supplementary estimated objective 3

For the primary estimation objective, a Per-Protocol Set (PPS) population analysis will be performed. The PPS is defined as: based on the randomized analysis set, cases that received at least one dose of TQB3616 (/placebo) and fulvestrant, met the protocol inclusion/exclusion criteria, and had no major protocol deviations; cases with both TQB3616 and fulvestrant interruptions exceeding 28 days due to COVID-19 infection/pandemic will also be excluded. The analysis method is consistent with the primary estimation method.

Investigator-assessed efficacy data will be used, and the median follow-up time for PFS in this study and its 95% CI will be estimated using the Reverse Kaplan-Meier method.

### 7.3.2 Analysis of Secondary Estimate Objectives/Secondary Endpoints

- IRC-assessed progression-free survival (PFS)

The Kaplan-Meier method was used to estimate PFS (independent radiologic assessment) with 95% CI and the Kaplan-Meier curve was plotted. The results of the stratified log-rank test were compared, and the stratified Cox proportional hazards model was used to estimate the HR. The 6-month, 12-month, and 18-month PFS rates will also be calculated.

- Overall survival (OS )

The median OS and 95% CI were estimated using the Kaplan-Meier method, and the Kaplan-Meier curve was plotted. The results of the stratified log-rank test were compared, and the stratified Cox proportional hazards model was used to estimate the HR. The 6-month, 12-month, and 18-month OS rates will also be calculated.

- Duration of response (DOR)

The median DOR and 95% CI were estimated using the Kaplan-Meier method, and Kaplan-Meier curves were plotted. Between-group comparisons were performed using the stratified log-rank test, and the between-group HR was estimated using a stratified Cox proportional hazards model. Analyses were also conducted for confirmed responses, unconfirmed responses, and assessments by both IRC and investigators.

- Objective response rate (ORR)

The ORR and 95% CI were calculated for both groups. The 95% CI was computed using the exact method based on Clopper-Pearson method. Between-group ORR comparisons were performed using the Cochran-Mantel-Haenszel test. The between-group odds ratio (OR) was also estimated using logistic regression. Analyses were conducted for confirmed responses, unconfirmed responses, and assessments by both IRC and investigators. Additionally, a supplementary analysis was performed in the population with measurable lesions, using consistent analytical methods.

- Disease control rate (DCR)

The DCR and 95% CI were calculated for both groups. The 95% CI was computed using the exact method based on Clopper-Pearson method. Between-group DCR comparisons were performed using the Cochran-Mantel-Haenszel test. The between-group odds ratio (OR) was estimated using logistic regression. Analyses were conducted for confirmed responses, unconfirmed responses, and assessments by both IRC and investigators.

- Clinical benefit rate (CBR)

The CBR and 95% CI were calculated for both groups. The 95% CI was computed using the exact method based on Clopper-Pearson method. Between-group CBR comparisons were performed using the Cochran-Mantel-Haenszel test. The between-group odds ratio (OR) was estimated using logistic regression. Analyses were conducted for assessments by both IRC and investigators.

- Time to First Response (TTR)

Descriptive analysis was used to calculate the time to first remission in both groups (TTR) .

## 7.4 Safety analysis

Safety analysis will be based on Safety Analysis Set (SS).

### 7.4.1 Drug Exposure Analysis

Drug exposure will be analyzed by actual treatment groups. For TQB3616 capsules and fulvestrant injections, the following will be calculated: exposure duration, cumulative dose, dose intensity, relative dose intensity, treatment interruption duration (due to COVID-19 pandemic, COVID-19 infection, or either), and time to first/second dose reduction (TQB3616 capsules only). For LHRH agonists, exposure duration, cumulative dose, and dose intensity will be calculated. Descriptive statistics (count, mean, standard deviation, maximum, minimum, median, quartiles) will be used; dose adjustments or treatment interruptions will be described using frequencies and percentages;

- The analysis indicators for TQB3616 and their definitions are as follows:

1. Exposure duration (days) is the sum of actual medication days; exposure duration (months) equals exposure duration (days) divided by 30.4375;
2. Cumulative administered dose (mg) equals the sum of all actual doses administered;
3. Dose intensity (mg/day) equals cumulative administered dose divided by exposure duration (days);
4. Relative dose intensity (%) = dose intensity / planned daily dose × 100%; The protocol specified a planned administration regimen of 180mg QD.
5. Time to first dose reduction (months) = (first dose reduction date - first administration date + 1) / 30.4375.
6. Time to second dose reduction (months) = (second dose reduction date - first administration date + 1) / 30.4375.

- The analytical parameters for fulvestrant and their definitions are as follows:

1. Cumulative administered dose (mg) equals the sum of actual doses administered per cycle;
2. Dose intensity (mg/administration) = cumulative administered dose / total number of drug exposures. In the first cycle, two doses will be administered on Day 1 and Day 15 respectively.
3. Relative dose intensity (%) = dose intensity / planned dose per administration × 100%. The protocol specified a planned dose of 500mg per administration.
4. Number of drug exposure cycles equals Cumulative Number of drug exposure cycles;

- The analytical measures for LHRH agonists and their definitions are as follows:

1. Number of drug exposure cycles equals Cumulative number of drug exposure cycles;
2. Cumulative administered dose (mg) equals the sum of actual doses administered per cycle;
3. Dose intensity (mg/cycle) = cumulative administered dose / total number of drug exposure cycles.

### 7.4.2 Adverse events

All adverse events (AEs) will be classified using the latest version of the Medical Dictionary for Regulatory Activities (MedDRA) at the start of coding and graded using CTCAE v5.0. If a subject experiences multiple AEs, they will be counted as one case for incidence calculations. If a subject experiences the same preferred term (PT) multiple times, they will be counted as one case for that PT. Similarly, if a subject experiences multiple AEs within the same system organ class (SOC), they will be counted as one case for that SOC.

Treatment-emergent adverse events (TEAEs) are defined as AEs that occur after the first dose and up to 28 days after the last dose.

1. Group by actual treatment, summarize the frequency, number of cases, and percentage of the following adverse events, with COVID-19 events analyzed separately in a dedicated table:

- Treatment-emergent adverse events (TEAEs)
- TEAEs related to the study drug (TQB3616/placebo or fulvestrant)
- TEAEs related to the study drug (TQB3616/ placebo)
- TEAEs related to the study drug (fulvestrant)
- Grade 3 or higher TEAEs
- Grade 3 or higher TEAEs related to the study drug (TQB3616/placebo or fulvestrant)
- Grade 3 or higher TEAEs related to the study drug (TQB3616/ placebo)
- Grade 3 or higher TEAEs related to the study drug (fulvestrant)
- Serious Adverse Event (SAE)
- SAEs related to the study (TQB3616/placebo or fulvestrant)
- SAEs related to the study (TQB3616/ placebo)
- SAEs related to the study (fulvestrant)
- Grade 3 or higher SAEs
- Grade 3 or higher SAEs related to the study drug (TQB3616 or fulvestrant/placebo or fulvestrant)
- Grade 3 or higher SAEs related to the study drug (TQB3616/placebo)
- Grade 3 or higher SAEs related to the study drug (fulvestrant)
- TEAEs leading to dose reduction (TQB3616/ placebo)
- TEAEs related to the study drug leading to dose reduction (TQB3616/ placebo)
- Grade 3 or higher TEAEs leading to dose reduction (TQB3616/placebo)
- Grade 3 or higher TEAEs related to the study drug leading to dose reduction (TQB3616/placebo)
- TEAEs leading to treatment discontinuation (TQB3616/Placebo or fulvestrant)
- TEAEs related to the study drug leading to treatment discontinuation (TQB3616/placebo or fulvestrant)
- TEAEs leading to treatment discontinuation (TQB3616/Placebo)
- TEAEs related to the study drug leading to treatment discontinuation (TQB3616/placebo)
- TEAEs leading to treatment discontinuation (fulvestrant)
- TEAEs related to the study drug leading to treatment discontinuation (fulvestrant)
- TEAEs leading to dose interruption (TQB3616/placebo or fulvestrant)
- TEAEs related to the study drug leading to dose interruption (TQB3616/placebo or fulvestrant)
- TEAEs leading to dose interruption (TQB3616/placebo)
- TEAEs related to the study drug leading to dose interruption (TQB3616/Placebo)
- TEAEs leading to dose interruption (fulvestrant)
- TEAEs related to the study drug leading to dose interruption (fulvestrant)
- Grade 3 or higher TEAEs leading to dose interruption (TQB3616 or fulvestrant/placebo or fulvestrant)
- Grade 3 or higher TEAEs related to the study drug leading to dose interruption (TQB3616 or fulvestrant/placebo or fulvestrant)
- Grade 3 or higher TEAEs leading to dose interruption (TQB3616/placebo)
- Grade 3 or higher TEAEs related to the study drug leading to dose interruption (TQB3616/placebo)
- Grade 3 or higher TEAEs leading to dose interruption (fulvestrant)
- Grade 3 or higher TEAEs related to study drug leading to dose interruption (fulvestrant)
- TEAE leading to death
- TEAEs related to study drug leading to death (TQB3616/placebo or fulvestrant)
- TEAEs related to study drug leading to death (TQB3616/placebo)
- TEAEs related to study drug leading to death (fulvestrant)

1. By System Organ Class (SOC) and Preferred Term (PT), describe the frequency, number of cases, and percentage of the above adverse events grouped by actual treatment, excluding COVID-19 events.
2. By actual treatment group, SOC, and PT classification, calculate the number of cases and incidence rate of each severity grade (per NCI CTCAE v5.0) and drug-relatedness, excluding COVID-19 events.

For repeated occurrences of the same AE, only the most severe instance is included in the analysis of case count and incidence rate by severity grade.

1. Time to first onset of TEAE analysis

Time to first TEAE onset = Date of first TEAE occurrence - First dose date + 1. Analysis will be conducted at overall, SOC, and PT levels.

Maximum TEAE duration = TEAE end date - TEAE start date + 1, with the maximum value taken for identical PTs. Maximum duration analysis will only be performed at PT level.

Only adverse reactions of special interest (related to TQB3616 or fulvestrant) will be analyzed. All-grade and grade 3-4 adverse reactions of special interest will be analyzed separately. For diarrhea and vomiting (2 special interest reactions), maximum duration of grade 2 events will also be analyzed individually.

1. Deaths, other serious adverse events, and adverse events of special interest (AESI)

Deaths include all fatalities during the study period and deaths resulting from procedures initiated during the study. Other serious adverse events refer to SAEs excluding death (including SAEs temporally associated with death or occurring prior to death).

Adverse events of special interest include: diarrhea, vomiting, decreased neutrophil count, anemia, alanine aminotransferase increased, and aspartate aminotransferase increased.

1. Provide a detailed list of subjects with adverse events

Includes: complete list of subjects with TEAEs, detailed subject list of SAEs, detailed subject list of deaths and deaths-related cases, detailed subject list of treatment discontinuations, and list of Grade 3 or higher TEAEs.

### 7.4.3 Laboratory tests

Laboratory quantitative index analysis

For continuous indicators of hematology and blood biochemistry, the number of cases, mean, standard deviation, median, quartiles, minimum and maximum at baseline and each visit will be described according to actual treatment groups, and the mean and standard deviation will be described for changes from baseline. Unscheduled visit results will not be presented.

Hematology includes the following Measures: Neutrophil count (10 ^ 9/L) ( NEUT ) , White blood cell count (10 ^ 9/L) ( WBC ) , Platelet count (10 ^ 9/L) ( PLT ), hemoglobin.

Chemistry includes the following Indicators: Alanine aminotransferase (U/L) ( ALT ), door Aspartate aminotransferase (U/L) ( AST ), total bilirubin (μmol/L) (TBIL ), creatinine (CRE ), triglycerides (TG ), potassium ( K ).

CROSS-TABLE ANALYSIS

Laboratory parameters were classified as low, normal, or high according to the normal range, and the changes from normal or normal baseline to low postbaseline and from normal or low baseline to high postbaseline were described. Postbaseline low or high was calculated using the lowest or highest postbaseline observation.

A data listing of laboratory test results will be presented for each visit by subject.

Analysis of laboratory parameters with potential clinical significance

Laboratory parameters will be measured according to the potential clinical importance (i.e. Potentially Clinically Important , PCI ) Criteria (Annex 2 ) for assessment (defined as per NCI CTCAE version 5.0 Criteria, Toxicity Grade ≥ 3) , grouped by actual treatment, description in accordance with individual PCI Number and percentage of subjects with criteria. And for the compliance PCI Standard subjects were tabulated.

Liver Function Analysis

Alanine aminotransferase (ALT), aspartate aminotransferase (AST), and total bilirubin (TBIL) will be categorized according to the following criteria and presented using cross-tabulation.

Alanine aminotransferase ( ALT ):

- > ULN - 3.0 x ULN
- > 3.0 - 5.0 x ULN
- > 5.0 - 20.0 x ULN
- > 20.0 x ULN

Glutamic-oxaloacetic transaminase ( AST ):

- > ULN - 3.0 x ULN
- > 3.0 - 5.0 x ULN
- > 5.0 - 20.0 x ULN
- > 20.0 x ULN

Total bilirubin (TBIL ):

- > ULN - 1.5 x ULN
- > 1.5 - 3.0 x ULN
- > 3.0 - 10.0 x ULN
- > 10.0 x ULN

Will be satisfied Hy's Subjects for the rule were grouped by actual treatment, and the number and percentage were described and listed.

Hy's Rule:

- ALT ≥ 3 × ULN
- AST ≥ 3 × ULN
- TBIL ≥ 2 × ULN

Meet any of the above criteria, if ALT ≥ 3 × ULN Or AST ≥ 3 × ULN Merge any of them TBIL ≥ 2 × ULN , will be specifically labeled.

### 7.4.4 Electrocardiogram

Continuous variables including heart rate, QT interval, and QTc interval will be summarized by actual treatment group using mean, standard deviation, median, quartiles, minimum, and maximum values for both measurements and changes from baseline.

ECG results will be descriptively summarized as normal, abnormal not clinically significant, or abnormal clinically significant. Shifts from baseline (normal or abnormal not clinically significant) to at least one post-dose abnormal clinically significant finding will be reported.

ECG findings at each visit will be descriptively summarized using the same three-category classification, with cross-tabulation against baseline results.

The proportion of subjects with clinically significant abnormalities among those showing any abnormal changes will be reported, with clinical significance determined by investigators.

### 7.4.5 Vital Signs

Vital signs will be summarized by actual treatment group, presenting mean, standard deviation, median, quartiles, minimum and maximum values for both measurements and changes from baseline.

### 7.4.6 ECOG Score

Subjects with increased ECOG scores relative to baseline will be summarized by count and percentage. Changes in ECOG scores before and after treatment will be presented using cross-tabulation.

### 7.4.7 Concomitant medication

Concomitant medications are defined as non-study medications that meet one of the following criteria:

1) All medications started after the first dose of the study drug or continued after the first dose of the study drug;

2) All medications started before the first dose of the study drug and continued after the first dose of the study drug.

The use of concomitant medications during the study (including changes during screening, new medications after screening, and medications during follow-up) will be summarized, along with the frequency of use for each medication.

Concomitant medications will be classified using the WHODrug Global ATC classification system and summarized by anatomical main group and active substance based on the SS population. A list of concomitant medications by treatment group will be provided.

## 7.5 Subgroup Analysis

Subgroup analyses will be performed for investigator-assessed PFS, IRC-assessed PFS, and investigator-assessed confirmed ORR. The three randomization stratification factors will be based on actual data recorded in EDC.

Subgroup factors include:

- - - Menopausal status: premenopausal or perimenopausal vs. postmenopausal
    - PR positive vs. PR negative
    - ECOG PS (0 vs. 1);
    - Presence of visceral metastasis (yes vs. no);
    - Lung metastasis (yes vs. no)
    - Liver metastasis (yes vs. no)
    - Non-visceral metastasis (bone metastasis only vs. others)
    - Baseline measurable disease (yes vs. no)
    - Age (≥ 65 vs. < 65);
    - Number of metastatic organs (≥ 3 vs. < 3);
    - Prior endocrine therapy agents (SERMs vs. AI vs. SERMs and AI)
    - Sensitivity to prior endocrine therapy (yes vs. no);
    - Prior rescue chemotherapy (yes vs. no);
    - Type of prior endocrine therapy ((only (neo) adjuvant endocrine therapy vs. only recurrent/metastatic endocrine therapy vs. (neo) adjuvant endocrine therapy and recurrent/metastatic endocrine therapy)
    - HER2 expression results (0 expression vs. low expression (1 + or 2 +))

# 8. Description of this plan

The analysis plan was drafted based on the relevant descriptions in the study protocol. In accordance with the fundamental characteristics of each endpoint and specific study requirements, statistical analysis methods for relevant evaluation metrics were proposed. Given the unpredictable nature of final clinical trial data distributions, the statistical analysis methods may require minor adjustments. Consequently, the presentation formats of statistical analysis results - including statistical tables, data listings, and figures - may also undergo certain modifications.

| Inconsistent with the protocol | Modification | Description |
| --- | --- | --- |
| The protocol does not specify the analysis of COVID-19 infection (or the COVID-19 pandemic) on PFS | Add “Supplemental Estimand 2” to account for the intercurrent event of “COVID-19 infection (or pandemic) causing treatment interruption >7 days”, which will be handled using a hypothetical strategy. For investigator-assessed PFS, subjects with treatment interruptions >7 days due to COVID-19 infection (or pandemic) will be censored | This analysis aims to evaluate the impact of COVID-19 infection (or the COVID-19 pandemic) on PFS. |
| Protocol did not address PPS analysis | Add “Supplemental Estimand 3” to include analysis of the Per-Protocol Set (PPS). The PPS is defined as: from the randomized analysis set, subjects who received at least one dose of TQB3616 (/placebo) and fulvestrant, met all eligibility criteria, and had no major protocol deviations. The analytical approach will be consistent with the primary estimand’s principal analysis method. | ICH E9(R1) states that PPS analysis may introduce bias. Therefore, the PPS analysis was not defined as a primary analysis in the protocol. To enable a more comprehensive, detailed, and scientific evaluation of drug efficacy, “Supplemental Estimand 3” has been added in the final SAP to assess treatment effects in the per-protocol population |

Attachments 1: Protocol deviation definition

Attachments 2: potentially clinically important criteria for laboratory parameters

Attached Form 2-1 . Blood routine

| Test | Level 1 | Grade 2 | Grade 3 | Grade 4 |
| --- | --- | --- | --- | --- |
| Neutrophil count decreased | < lower limit of normal – 1.5 × 10 ^9^ /L | < 1.5 – 1.0 × 10 ^9^ /L | < 1.0 – 0.5 × 10 ^9^ /L | < 0.5 × 10 ^9^ /L |
| White blood cell count decreased | < lower limit of normal – 3.0 × 10 ^9^ /L | < 3.0 – 2.0 × 10 ^9^ /L | < 2.0 – 1.0 × 10 ^9^ /L | < 1.0 × 10 ^9^ /L |
| Platelet count decreased | < lower limit of normal – 75.0 × 10 ^9^ /L | < 75.0 – 50.0 × 10 ^9^ /L | < 50.0 – 25.0 × 10 ^9^ /L | < 25.0 × 10 ^9^ /L |
| Hemoglobin decreased | < lower limit of normal – 100 g/L | < 100 - 80 g/L | < 80 g/L | - |

Note: Post-baseline CTCAE Toxicity grade above baseline.

Attached Form 2-2 Blood biochemistry

| Test | Level 1 | Grade 2 | Grade 3 | Grade 4 |
| --- | --- | --- | --- | --- |
| Alanine aminotransferase increased | Normal Baseline |  |  |  |
|  | > 3 x upper limit of normal | > 3-5 times the upper limit of normal | > 5 – 20 times the upper limit of normal | > 20 x upper limit of normal |
|  | Abnormal Baseline |  |  |  |
|  | > 1.5-3 times baseline value | > 3-5 times baseline | > 5 – 20 times baseline value | > 20 x baseline |
|  |  |  |  |  |
| Aspartate aminotransferase increased | Normal Baseline |  |  |  |
|  | > 3 x upper limit of normal | > 3-5 times the upper limit of normal | > 5 – 20 times the upper limit of normal | > 20 x upper limit of normal |
|  | Abnormal Baseline |  |  |  |
|  | > 1.5-3 times baseline value | > 3-5 times baseline | > 5 – 20 times baseline value | > 20 x baseline |
|  |  |  |  |  |
| Total bilirubin increased | Normal Baseline |  |  |  |
|  | > 1.5 x upper limit of normal | > 1.5 – 3.0 times the upper limit of normal | > 3.0 – 10 times the upper limit of normal | > 10 x upper limit of normal |
|  | Abnormal Baseline |  |  |  |
|  | > 1-1.5 times baseline | > 1.5 – 3.0 times baseline | > 3.0 – 10 times baseline | > 10 times baseline value |

Note: Post-baseline CTCAE Toxicity grade above baseline.
